# The serine protease HTRA1 targets tau fibrils and provides a proteolytic barrier against pathogenic protein conformations

**DOI:** 10.1016/j.jbc.2025.110729

**Published:** 2025-09-16

**Authors:** Birte Hagemeier, Kamilla Ripkens, Nina Schulze, Anika Bluemke, Michal Strzala, Michelle Koci, Farnusch Kaschani, Markus Kaiser, Michael Erkelenz, Sebastian Schluecker, Melisa Merdanovic, Simon Poepsel, Doris Hellerschmied, Steven G. Burston, Michael Ehrmann

**Affiliations:** 1 Center of Medical Biotechnology, Faculty of Biology, University Duisburg-Essen, Essen, Germany; 2 Research Institute of Molecular Pathology, Hasselbach Group, Vienna, Austria; 3 Department of Chemistry and Center for Nanointegration Duisburg-Essen (CENIDE), University of Duisburg-Essen, Essen, Germany; 4 Center for Molecular Medicine Cologne (CMMC), Faculty of Medicine and University Hospital, University of Cologne, Cologne, Germany; 5 Cologne Excellence Cluster for Cellular Stress Responses in Ageing-Associated Diseases (CECAD), University of Cologne, Germany; 6 School of Biochemistry, University of Bristol, Bristol, United Kingdom

**Keywords:** HTRA1, PDZ proteases, amyloid fibrils, tau, Alzheimer's disease

## Abstract

Tauopathies such as Alzheimer’s disease, frontotemporal dementia with Parkinsonism, and other neurodegenerative diseases are classified as protein folding diseases because they share amyloid fibrils as a hallmark. Typically, amyloid fibrils accumulate and spread through tissue over time. It is assumed that this process is accelerated as protein quality control becomes overwhelmed in aged tissues. However, a deep understanding of how specific protein quality control factors interfere with fibril accumulation and thereby delay disease onset is lacking. Here, we demonstrate that the widely conserved serine protease HTRA1 is activated by tau fibrils, providing quantitative, topological, and temporal insights into the proteolytic degradation of both soluble and fibrillar tau. Live cell fluorescence microscopy demonstrates the interaction of HTRA1 with tau fibrils and their proteolytic degradation in cells. Our data highlight the potential of HTRA1 to act in a cell non-autonomous defense mechanism against the intercellular spread of pathogenic protein conformations.

Amyloid fibrils are the hallmark of prominent neurodegenerative diseases. Their structures consist of tightly packed β-sheets, in which regions of identical amino acid sequences are stacked on top of each other (1). Alzheimer's disease (AD) is characterized by the presence of amyloid fibrils composed of extracellular Aβ peptides and of intracellular tau protein (microtubule associated protein tau). Tau dissociates from microtubules upon hyperphosphorylation before forming fibrillar structures (2, 3). The core of amyloid tau fibrils is formed by residues 306 to 378 (in the numbering of the 441-residue human tau isoform), corresponding to the microtubule-binding repeats R3 and R4, and some amino acids following R4, while the rest of the protein forms the so-called fuzzy coat (4). Disease progression is caused by the spread of fibrils from one brain region to connected areas, accompanied by cognitive impairment. The individual steps of propagation are initiation of aggregation in a donor neuron, externalization of a transmissible tau species, intercellular transfer, and internalization in a recipient cell, followed by seeding with endogenous tau (5). Seeding promotes fibril formation when carried out with purified proteins but also in cells where a doubling time of self-replication of small tau aggregates can be 5 h in HEK293 cells or 1 day in primary neurons (6).

From a protein quality control perspective, tau aggregates can be considered a moving target, requiring either dedicated factors in different cellular compartments or factors that co-migrate with the transmissible species. The secreted serine protease HTRA1 is a member of the high temperature requirement A (HtrA) family implicated in protein quality control (7). HTRA1 senses protein folding stress by binding to exposed C-termini of misfolded, fragmented, and mislocalized proteins *via* its C-terminal PDZ domain. Although HTRA1 is a secreted protein, it is also found in the cytosol because it can re-enter cells (8, 9, 10). HTRA1 has been implicated in severe pathologies such as age-related macular degeneration, arthritis, cancer, and familial ischemic cerebral small vessel disease (7). In the context of neurodegenerative diseases, HTRA1 degrades tau fibrils through a unique mechanism in which fibril dissociation precedes proteolysis, suggesting its role in tau pathology (10). In human AD brain extracts, levels of neurofibrillary tangles and HTRA1 are negatively correlated (11). Consistently, a proteomic study that investigated human brain samples from AD patients found a strong association of HTRA1 with detergent-insoluble tau (12). In addition, colocalization of HTRA1 and neurofibrillary tangles was demonstrated (13).

Here, we show that tau fibrils activate the HTRA1 protease. We also provide quantitative insights into the proteolytic degradation of soluble and fibrillar tau at high topological and temporal resolution. To gain further cell biological insights into the interaction of HTRA1 with tau fibrils, we used live cell fluorescence microscopy to quantify the uptake of HTRA1 into cells harboring tau fibrils, the interaction between HTRA1 and tau fibrils, and the HTRA1-dependent decrease in tau fibrils over time.

## Results

### Activation of HTRA1 by tau fibrils and identification of binding sites

**Figure 1:**
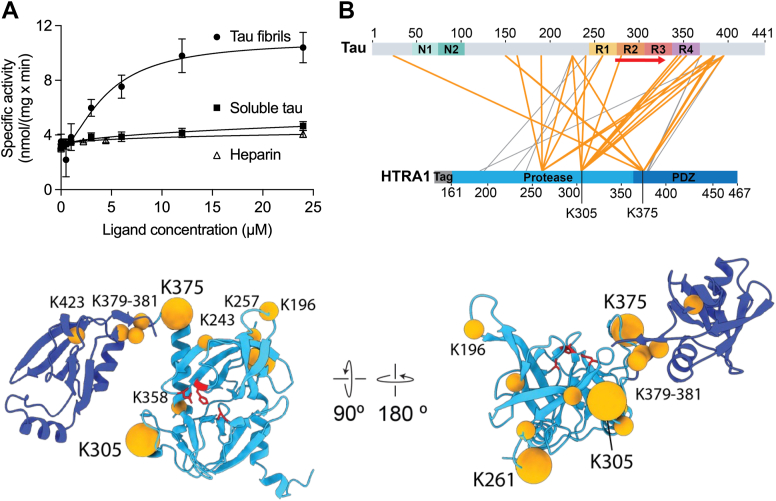
**Activation of HTRA1 and identification of substrate interaction sites.***A*, activation of HTRA1 by tau fibrils. The specific activity of purified HTRA1 in the presence of soluble and fibrillar tau was determined using the synthetic substrate VFNTLPMMGKASPV-pNA. Additionally, the effect of heparin on HTRA1 activity was analyzed, given that heparin was utilized to induce the formation of tau fibrils. HTRA1 was mixed with pNA substrate, at the tau concentrations indicated. Proteolysis of the pNA substrate was continuously measured at 405 nm and used to calculate the specific enzyme activity. n = 3. *B*, cross-linking mass spectrometry analysis of HTRA1^S328A^ binding to tau fibrils. Schematic representation of intermolecular cross-links between tau and HTRA1. Cross-links involving the most frequently cross-linked residues of HTRA1 (K261, K305, and K375) are highlighted in *orange*. N1, N2: N-terminal inserts, R1-4: microtubule-binding repeat regions of tau. *Red arrow*: β-sheet region of tau fibrils (PDB ID: 6QJH). HTRA1 monomer model including the protease (*light blue*) and PDZ domains (*dark blue*). *Orange* spheres mark the HTRA1 residues with intermolecular cross-links to tau. Most frequently cross-linked residues are highlighted by larger spheres. Catalytic residues of the active site are shown in *red* and in ball-and-stick representation. Model based on an alphafold2 prediction. Note that the PDZ domain displays flexibility relative to the protease domain and is not in a fixed position.

HTRA1 is regulated by the mechanism of ligand-induced activation. Such ligands can be peptides or folded proteins (14, 15). To test whether tau fibrils activate HTRA1, protease assays of recombinant HTRA1 were performed using the artificial colorimetric substrate VFNTLPMMGKASPV-pNA (16) in the presence of soluble or fibrillar tau. HTRA1 was up to >2.5-fold more active in the presence of tau fibrils but not soluble tau (Figs. 1*A* and S1). These data suggest that tau fibrils interact more tightly with the active site of HTRA1 than soluble tau and that the activation by fibrils supports their efficient degradation.

To investigate how HTRA1 engages with tau fibrils, we performed cross-linking mass spectrometry using the inactive HTRA1^S328A^ variant, where the catalytic Ser328 residue is exchanged by Ala, tau fibril seeds, and the cross-linking reagent PhoX (3,5-Bis(((2,5-dioxopyrrolidin-1-yl)oxy)carbonyl)phenyl)phosphonic acid) (17). Mass spectrometry identified three residues within HTRA1 that are extensively cross-linked to tau fibril seeds: K305 in the sensor loop L3, K375 located at the border of the protease and PDZ domains, and K261 located in a loop opposite the active site. Further cross-links clustered at the connecting region between protease and PDZ domains, including the N-terminal part of the PDZ domain as well as lysines at the peripheral face of the HTRA1 trimer (Fig. 1*B* and Data S1). Functionally, K305 located in the sensor loop L3 is of interest since it is a key element of the activation domain of HTRA1, which allows for the ligand-induced activation of the proteolytic activity by triggering a disorder-to-order transition of the active site (16, 18). Cross-links near the PDZ domain and on the peripheral surface of the protease domain indicate how HTRA1^S328A^ initially interacts with tau fibrils. Interestingly, the amino acids of tau found to cross-link to these regions of HTRA1 are located primarily in the C-terminal portion, flanking the β-sheet-forming fibrillar core of tau (Fig. 1*B*). No cross-links were detected closer to the active site of HTRA1, which could be explained by the binding of tau near the active site potentially excluding the cross-linker from access to reactive side chains. Lys residues that react preferentially with PhoX are unlikely cleavage sites because HTRA1 cleaves preferentially after small hydrophobic residues (16). However, tau residues K353, K385, and K395, which are cross-linked to the L3 loop of HTRA1, are in close proximity to residues L376, I392, and L408, which are cleaved early and very efficiently (see below for details).

### High-resolution analysis of HTRA1-dependent proteolytic processing of tau

**Figure 2:**
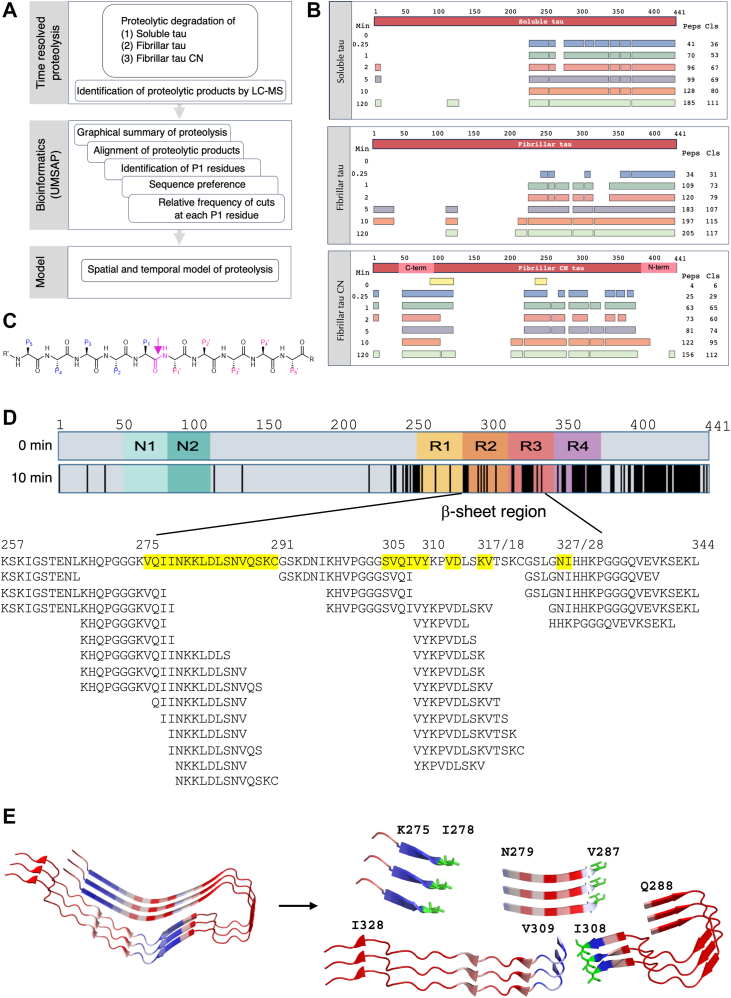
**Proteolytic degradation of soluble and fibrillar tau by HTRA1.***A*, Workflow, see text for details. *B*, time-resolved proteolysis of soluble and fibrillar forms of tau by HTRA1. Top bar: linear representation of the entire substrate protein. For each of the indicated time points (Min), peptide sequences that align without gaps are grouped into fragments, shown as bars. The number of identified peptides (Peps) and the total number of cleavage sites (Cls) are shown on the *right*. Identified peptides aligned to the primary amino acid sequence of tau are shown in Supporting Data 2 (soluble tau), 4 (fibrillar tau) and 5 (fibrillar tau CN, where parts of the N- and C-terminal regions have been swapped). *C*, model peptide and standard nomenclature. The residue of the scissile bond (*magenta*) is termed P1, which is often the major determinant of substrate specificity. Residues upstream to P1 are termed P2, P3 *etc.* Residues located downstream to P1 are termed P1′, P2', P3′ *etc.* (56). The proteolytic cleavage site is marked by an *arrow*. *D*, top bar, graphic representation of 2N4R tau. The conserved N-domains and repeat regions are highlighted. *Lower bar*, *vertical black lines* indicate P1 residues that are cleaved after 10 min of incubation with HTRA1. The β-sheet region of fibrils is highlighted; numbers indicate the amino acids present as β-sheets. Amino acid sequence alignment covering residues K257-L344, β-sheet forming residues are highlighted in *yellow*. Peptides below the primary amino acid sequence correspond to proteolytic products identified by MS. *E*, early cleavage events in fibrillar tau. Following cuts at major proteolytic sites, the fibrillar core (*left*, PDB ID: 6QJH) is initially processed into 4 fragments (*right*). Key P1 residues I278, V287 and I308 (*green*) are shown as sticks. The colour code represents the B-factors, ranging from structural rigidity (*blue*) to flexibility (*red*). See text for details.

To better understand the dynamics of tau fibril proteolysis by HTRA1, we used time-resolved mass spectrometry (MS) in combination with bioinformatic analyses to identify proteolytic products. The sequences of the identified proteolytic products are aligned along the primary amino acid sequence of the substrate, and the relative frequency of cuts at each cleavage site is calculated (Figs. 2*A*, S2, and Datas S2, S4, and S5).

Before studying the proteolysis of tau fibrils, we examined the degradation of soluble tau (19). Soluble tau was incubated with HTRA1, and samples were taken at seven time points within 120 min. MS data were analyzed using UMSAP software (18, 20) to identify proteolytic products and the number of individual cleavage sites at each time point (Fig. 2*B*). These data identified 36 sites after 15 s and 111 sites after 120 min of incubation. Surprisingly, the N-terminal half (residues 1–225) appeared to be protease resistant, as the only four cleavage sites that were detected were identified only once and at late time points (Figs. 2*B*, S2*A*, and Data S2). The downstream region comprising residues 226 to 274 contained 11 cleavage sites, six of which were cleaved only once after 120 min of incubation. In contrast, the remaining C-terminal region contained the seven most frequently detected cleavage sites and was thus most efficiently cleaved (Fig. S2*A* and Tables S1 and S2).

Each proteolytic product is the result of two cuts. One at its N-terminus, where the N-terminal residue represents the P1′ residue, and another at its C-terminus, where the C-terminal residue represents the P1 residue (Fig. 2*C*). UMSAP also calculates the relative frequency of cuts at each P1 residue for each time point (see Experimental procedures for details). These data provide quantitative information on how well HTRA1 cleaves the substrate at each position within its primary amino acid sequence and how proteolysis progresses over time (Fig. S2*A*). Thus, individual P1 residues can be grouped into classes, based on histograms (Fig. S2, *E*–*G*). The first class consisted of 7 P1 residues where the relative frequency of cuts reached ≥20 *i.e.*, 22 to 51 (V256, C291, I308, C322, L376, I392, L408). The second class consisted of 10 P1 residues with a relative frequency of cuts of 10 to 19 (L266, I277, I278, V287, S289, I328, L344, V411, L428, V432) (Fig. S2 and Table S2). Members of the third class with a relative frequency of cuts from 1 to 10 were considered poor sites at all time points, probably due to their low affinity to the active site. The fourth class consisted of residues for which no proteolytic products could be detected, either because these sites did not bind to the active site, or the resulting products were not detected by MS. These data serve as a reference for the analysis of HTRA1-dependent proteolysis of amyloid tau conformations.

### Proteolysis of fibrillar tau by HTRA1

For tau fibrils, time-resolved MS revealed 31 cleavage sites after 15 s and 117 sites after 120 min of incubation (Fig. 2*B*). Already after 1 min of incubation, the entire β-sheet-forming portion of tau was processed into 12 peptides (Data S4), after 10 min into 28 peptides (Fig. 2*D*), and after 120 min into 31 peptides (Data S4).

Based on histograms (Fig. S2, *E*–*G*), P1 sites were grouped into classes according to the maximum relative frequency of cuts performed at each site. Class 1 (≥20 cuts, *i.e.*, 25–59) comprised five residues (Table S1), and Class 2 (10–19 cuts) comprised 9 residues (Fig. S2*B* and Table S2). The P1 sites with the highest relative number of cuts at the earliest time points are candidate sites where the proteolysis of fibrils is initiated. These sites are L408 and A392, followed by L376 and L344, located downstream of the fibrillar core. Upstream of the fibrillar core, HTRA1 cleaves after V256 and subsequently after L266, which is located 9 residues upstream of the fibril core. Together, these cuts produce a fragment of K267-L344, containing the entire fibril core. Subsequently, K267-L344 is fragmented by cuts after I308 (in β-sheet 2) and I278 and V287 (in β-sheet 1) into 4 peptides containing 9, 9, 20 and 35 residues respectively, before these peptides are successively degraded into small peptides of a minimum size between 7 and 17 residues (Figs. 2, *D* and *E*, S2*B*, and Data S4). Likewise, the region downstream of the fibrillar core is degraded into small peptides by cuts at 74 sites (Fig. S2*B* and Data S4).

As in the case of soluble tau, the N-terminus of fibrillar tau was a poor substrate for HTRA1 (Figs. 2*B*, S2*B*, and Data S4). In addition, we observed other common features for the soluble and fibrillar substrates. The appearance of many proteolytic products sharing only one identical cleavage site suggests that these products are the result of one high-affinity and several low-affinity binding sites (Datas S2, S4, and S5). This model is supported by the observed tendency for longer proteolytic fragments, again sharing an identical cleavage site, to appear preferentially at later time points, suggesting that these additional cleavage sites are of even lower affinity. A comparison of similarities and differences in cleavage efficiency at specific sites suggests areas of differential accessibility in soluble and fibrillar tau (Tables S1 and S2). Differences in cleavage efficiency are mainly observed for residues in the fibril core region, which are cleaved more frequently in soluble tau compared to fibrillar tau (Table S3), as expected due to the tight packing of the fibril core.

### Proteolysis of tau derivatives with swapped N- and C-termini

To address the curiosity of the unexpected protease resistance of the N-terminal region and the pronounced protease sensitivity of the C-terminal region of tau, we swapped the C-terminal residues 391 to 441 with the N-terminal residues 42 to 98 to generate the tau CN construct. Tau CN fibrils were incubated with HTRA1, and the resulting cleavage products were analyzed as described above. 29 cleavage sites were detected after 15 s and 112 sites after 120 min of incubation (Fig. 2*B*), which is comparable to the digests of wt tau fibrils (Fig. 2*B*). Notably, the cleavage pattern of the swapped C-terminal and N-terminal parts of tau CN changed only slightly or not at all compared to wt tau fibrils (Fig. S2*C* and Data S5). These data support the notion that the N-terminal part of tau is a poor substrate for HTRA1 due to its amino acid sequence rather than its position within the polypeptide. In addition, swapping the N- and C-terminal fragments had only a minor effect on the cleavage pattern of the β-sheet core region (Fig. S2*C* and Data S5). Calculation of the amino acid distribution at the five positions before or after the scissile bond showed no major changes in the three substrates analyzed (Fig. S2*D*). However, the relative number of cuts at each cleavage site and time point was generally lower for tau CN fibrils compared to the digests of soluble and wt fibrillar tau, suggesting that tau CN fibrils were digested more slowly. Our data provide a framework to address the molecular mechanisms underlying the protease resistance of tau’s N-terminal region and to determine whether and how its C-terminus contributes to efficient fibril degradation.

### HTRA1 reduces tau seeding *in vitro*

**Figure 3:**
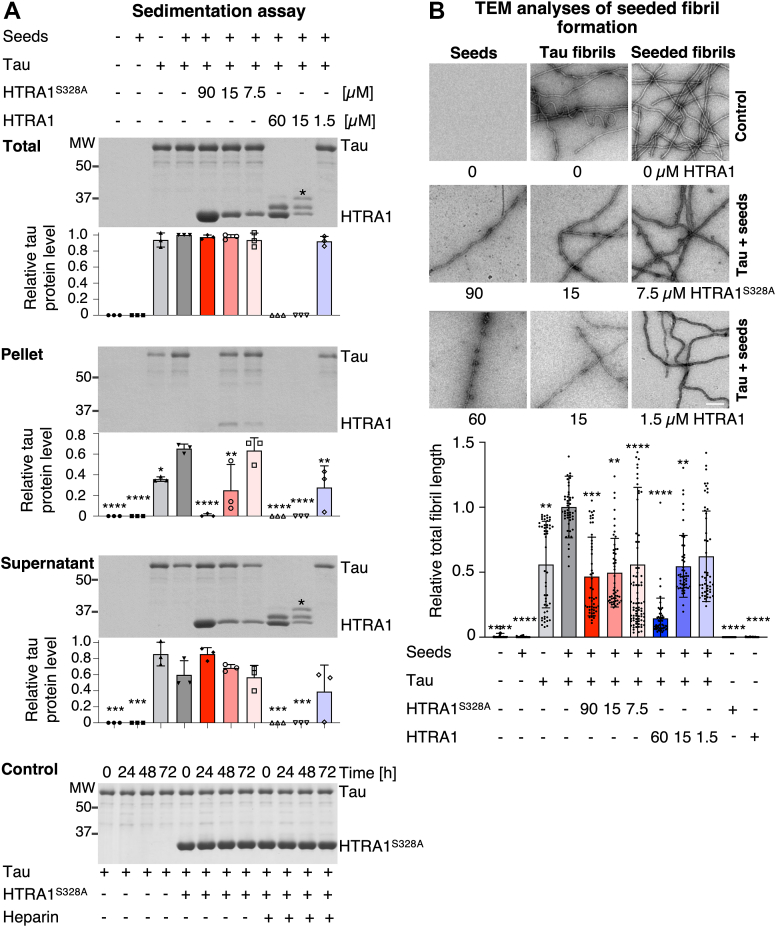
**HTRA1 reduces seeded tau aggregation independent of its proteolytic activity.** Soluble tau (30 μM) was incubated with tau seeds (0.6 μM) in aggregation buffer for 3 days at 37 °C. Proteolytically active HTRA1 (1.5, 15, 60 μM) or inactive HTRA1^S328A^ (7.5, 15, 90 μM) was applied to study the solubilization and degradation of tau fibrils. For solubilization, seeds were preincubated with HTRA1^S328A^, while HTRA1 was added directly to soluble tau and seeds. *A*, sedimentation assay. Samples were subjected to ultracentrifugation. Total, pellet, and supernatant (Sup.) fractions were subjected to SDS-PAGE and Coomassie staining. Bands marked with an *asterisk* represent proteolytic products of tau. Tau protein levels were quantified by densitometry. Values are normalized to the total amount of tau co-incubated with seeds (*dark grey bar* at total tau data) and used for the analysis of significant differences relative to untreated tau samples. Individual data points of replicates are shown. Error bars indicate standard deviation (SD); n = 3. ∗*p* < 0.05, ∗∗*p* < 0.01, ∗∗∗*p* < 0.001, ∗∗∗∗*p* < 0.0001. One-way ANOVA with Dunnett´s posttest. Control: to demonstrate that the tau, HTRA1^S328A,^ and heparin samples did not contain any protease activity, 30 μM tau protein was incubated with or without 90 μM HTRA1^S328A^ or 0.4 mg/ml heparin for 0, 24, 48, and 72 h. Aliquots were subjected to SDS PAGE and Coomassie staining. *B*, samples from (*A*) were further analyzed by transmission electron microscopy (TEM). Quantification of the total fibril length per image is based on at least 25 images (7.89 μm × 7.89 μm) per condition and replicate. Co-incubation of tau and seeds (*dark grey bar*) was used for the normalization of values and the analysis of significant differences relative to untreated tau samples. Individual data points (total fibril lengths of each image) are shown. Error bars indicate standard deviation (SD); n = 2. ∗∗*p* < 0.01, ∗∗∗*p* < 0.001, ∗∗∗∗*p* < 0.0001; Kruskal–Wallis test with Dunn's post-test. Representative TEM images; scale bar: 250 nm.

Since HTRA1 is able to digest soluble and fibrillar tau, the latter by combining fibril dissociation and proteolysis (10, 11), we hypothesized that this secreted protein quality control factor might also be involved in the defense against intercellular spreading of tau aggregation. To provide direct biochemical evidence that HTRA1 is interfering with the seeding process, we performed experiments with purified proteins. Seeded aggregation of tau was modeled *in vitro* by co-incubating soluble tau with seeds. Proteolytically inactive HTRA1^S328A^ was pre-incubated with tau seeds to induce their disassembly, while proteolytically active HTRA1 was added directly to soluble tau and seeds. Following incubation to allow seeded fibril formation, a sedimentation assay was performed to distinguish between soluble tau in the supernatant and aggregated tau in the pellet fraction. In this assay, the addition of seeds increased the amount of pelleted tau 1.8-fold compared to soluble tau alone (Fig. 3*A*). To assess the amount of fibrillar tau, the pellet fraction was subjected to negative-stain transmission electron microscopy, and the total fibril length was calculated by adding the lengths of individual fibrils as previously established (10). Upon addition of tau seeds, the total fibril length was increased 1.8-fold (Fig. 3*B*), consistent with sedimentation assays (Fig. 3*A*). HTRA1^S328A^ caused a concentration-dependent shift of tau from the pellet to the supernatant fraction (Fig. 3*A*). At the highest concentration of HTRA1^S328A^, 88% of the tau protein was found in the supernatant, while HTRA1^S328A^ was predominantly soluble. At the highest concentration of HTRA1^S328A^, the total fibril length was reduced to less than 50% when compared to the control (Fig. 3*B*). Tau polymerization was also monitored by measuring ThT fluorescence (Fig. S3). Treatment with HTRA1^S328A^ resulted in consistently low ThT fluorescence, indicating reduced nucleation of tau aggregates. Proteolytic products resulting from cleavage by active HTRA1 did not pellet upon ultracentrifugation, indicating that HTRA1-mediated degradation of tau did not increase its aggregation propensity (Fig. 3*A*). Consistently, increasing HTRA1 amounts reduced total fibril length by 86% and decreased ThT fluorescence to background levels (Fig. S3).

### HTRA1 colocalizes with tau seeds in cells

Live cell spinning disk confocal microscopy was used to quantify the colocalization of intracellular HTRA1 during the uptake of tau seeds and the uptake of HTRA1 into cells already containing tau seeds over 30 h. SH-SY5Y neuroblastoma cells were treated either with conditioned media containing secreted HTRA1^S328A^-mCherry (H) followed by DyLight 488-labeled tau seeds (S) or *vice versa* (referred to as HS and SH, respectively) (Videos S1 and S2). The number of discrete fluorescent mCherry and DyLight 488 spots was quantified over time. To minimize the likelihood of artificial colocalization caused by simultaneous uptake, complete medium exchange and washing steps were performed before adding the second protein in these sequential protein addition experiments.

**Figure 4:**
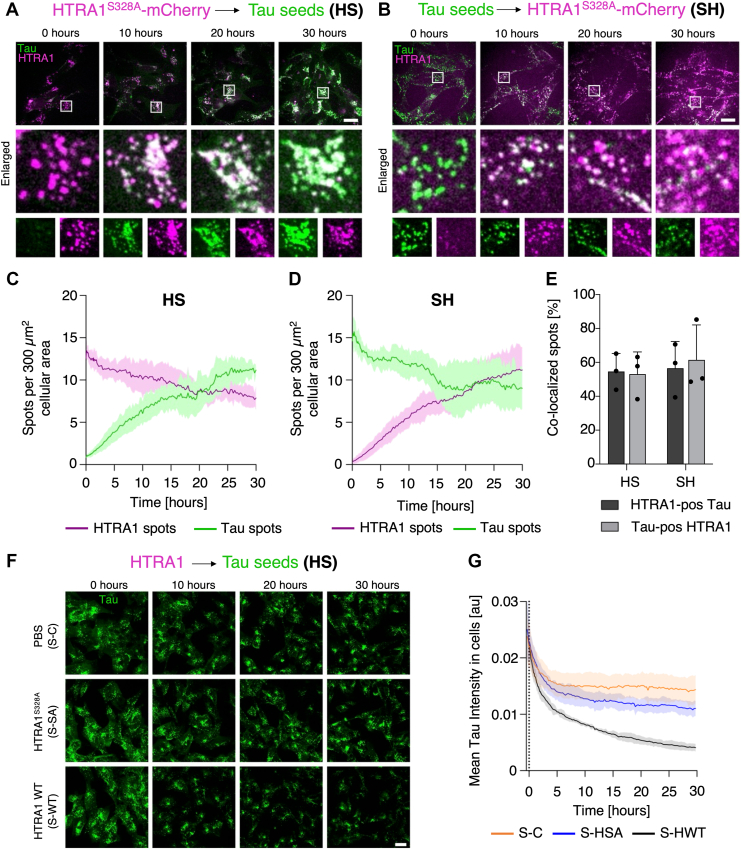
**HTRA1 colocalizes with and degrades tau aggregates in recipient cells.***A*, live cell imaging of SH-SY5Y cells treated with conditioned medium containing secreted HTRA1^S328A^-mCherry (*magenta*) 24 h before imaging. Cells were treated with DyLight 488-labeled tau seeds (*green*) immediately prior to imaging (HS treatment). Enlarged regions labeled in the top *panel* are shown as overlay (*middle panel*) and single channels (*lower panel*). *B*, live cell imaging of SH-SY5Y cells treated with DyLight 488-labeled tau seeds (*green*) 24 h before imaging. Subsequently, cells were treated with conditioned medium containing secreted HTRA1^S328A^-mCherry (*magenta*) immediately prior to imaging (SH treatment). Enlarged regions marked in the *upper panel* are shown as overlay (*middle panel*) and single channels (*lower panel*). Scale bars: 20 μm. The intensity of the *green* (tau) channel is scaled differently for sub-figures *A* and *B* (A - HS: 0–350, B – SH: 0–140), to allow better visibility of individual spots. As the treatments were conducted 20 min before the start of imaging, there were already very few spots visible at the 0 h time point. *C* and *D*, time-resolved analysis of HTRA1^S328A^-mCherry and DyLight 488-labeled tau seed spots in SH-SY5Y cells (10 random positions per condition/experiment, n = 3; in total, >10,500 individual HTRA1 and Tau spots were analyzed). Cells were either treated first with HTRA1^S328A^-mCherry followed by tau seeds (HS shown in *C*) or *vice versa* (SH shown in *D*). In each frame, the CellTracker *Deep Red* stain was used to detect the image area that was covered with cells (total cell area). Discrete fluorescent spots were detected in the mCherry and DyLight 488 channel in this region only. The number of detected discrete HTRA1^S328A^-mCherry (*magenta line*) and/or tau seed spots (*green line*) was normalized by the measured cell area (number of spots/μm^2^) and multiplied by the factor 300, which reflects the average cell area of an individual SH-SY5Y cell. Data show mean ± SD. Quantification of controls and example movies are shown in Fig. S4 and Videos S1 and S2. *E*, co-localization analysis of HTRA1^S328A^-mCherry and DyLight 488-labeled tau seed spots at time point 20 h after start of imaging. Discrete spots of HTRA1^S328A^-mCherry were detected and masked with DyLight 488-labeled tau seed spots to identify double-positive species (tau-positive HTRA1 spots) or *vice versa* (HTRA1-positive tau spots) (data are presented as mean ± SD (n = 3); dots represent mean values from individual experiments). Time-resolved analysis of co-localization data is shown in Fig. S4*F*, live cell imaging of SH-SY5Y cells treated with DyLight 488-labeled tau seeds (*green*) 24 h prior to imaging. Control cells were treated with either PBS (*top panel*, S-C), DyLight 633 HTRA1^S328A^ (*middle panel*, S-SA), or DyLight 633 HTRA1 WT (*bottom panel*, S-WT) on the microscope stage and imaging was continued for 30 h. Scale bars: 20 μm. Merged images showing the uptake of HTRA1 species and their co-localization with tau seeds are shown in Fig. S4, *I* and *K*. *G*, time-resolved analysis of intracellular tau signal intensity. Cell areas covered by SH-SY5Y cells (total cell area) were detected based on CellTracker *Orange staining*. Mean intensity of DyLight 488 in this total cell area was measured for each time point (10 random positions per condition/experiment, n = 3; in total, >50,000 individual HTRA1 and Tau spots were analyzed). Dashed line indicates time point of addition of PBS (S-C), DyLight 633 HTRA1^S328A^ (S-HSA), or DyLight 633 HTRA1WT (S-HWT). The signal decrease in the first 2 min is due to photobleaching effects caused by the higher frame rates of the imaging.

Efficient uptake was observed for extracellular tau seeds (HS treatment, Figs. 4, *A* and *C* and S4*A*) and secreted HTRA1^S328A^-mCherry (SH treatment, Figs. 4, *B* and *D* and S4*B*). For both conditions, the number of discrete intracellular spots increased over time (Fig. 4, *C* and *D*). For the subpopulation already present in the cytosol (HTRA1 in HS and Tau in SH treatment), we observed a decrease in the number of spots over time. A similar change was observed for the respective control treatments, where DMEM was applied instead of extracellular protein (Fig. S4, *C*–*F*). Despite photobleaching effects, this is most likely due to the condensation of aggregates into larger complexes.

Strikingly, a rapid co-localization was observed shortly after the internalization of the extracellularly applied proteins for both conditions, which slightly decreased over time, most likely due to the oversaturation compared to the population already present in the cytosol (Fig. S4*G*, HTRA1-positive tau spots and Fig. S4*H*, tau-positive HTRA1 spots). For the subpopulation already present in the cytosol (HTRA1-positive tau spots in SH treatment, tau-positive HTRA1 spots in HS treatment), the degree of colocalization increased steadily over time. Based on >10,500 HTRA1 and tau spots, respectively, we detected up to 60% colocalization in both conditions (Fig. 4*E*).

### HTRA1 reduces the formation of seeded tau aggregates in cells

To test whether HTRA1 can rescue acceptor cells from tau aggregates, live-cell imaging was performed with purified recombinant proteolytically active HTRA1. In these experiments, SH-SY5Y neuroblastoma cells containing DyLight 488-labeled tau seeds were imaged for 20 min before the addition of DyLight 633-HTRA1 (Fig. 4*F*). Measurements of DyLight 488 (tau) intensity in the cells over 30 h showed a marked decrease in signal following the uptake of active DyLight 633-HTRA1 (Fig. 4*G*), suggesting that HTRA1 reduces the level of tau aggregates in cells, consistent with its proteolytic activity (Videos S3 and S4). The reverse setup was also performed, where labeled tau seeds were introduced into cells preloaded with DyLight 633-HTRA1 WT (Fig. S4*I*). As in the previous experiment, the DyLight 488 (tau) signal decreased over time, but to a lesser extent (Fig. S4*J*). This could be explained by the inaccessibility of the tau seeds when they are still localized in endosomes and/or the requirement for longer incubation times to efficiently visualize tau degradation. Both experiments were also performed with proteolytically inactive HTRA1^S328A^ to see if colocalization could be observed, as previously shown with conditioned media containing HTRA1^S328A^-mCherry. We observed the uptake of DyLight 633-HTRA1^S328A^ and its colocalization with tau aggregates in all conditions (Figs. 4*I* and S4, *J*–*L*). Taken together, these results highlight the dynamic interaction between HTRA1 and tau aggregates, which, in the case of wt HTRA1, results in a reduction of aggregated tau levels.

## Discussion

As an ATP-independent enzyme, HTRA1 is uniquely suited to function intra- and extracellularly, offering advantages over previously identified factors involved in amyloid fibril dissociation or degradation. For example, while ATP-dependent heat shock chaperones and the AAA^+^ ATPase VCP can dissociate tau fibrils from various sources, including patient brains, the extracted polypeptides remain seedable, presumably because they are not degraded by a protease (21, 22, 23, 24). However, HTRA1 may still cooperate with these or other proteostasis factors that primarily dissociate fibrils. One notable example is the Calpain 2-HTRA1 complex, where the cysteine protease Calpain 2 allosterically activates HTRA1 (14). A more general protective role for HTRA1 is suggested by a recent report indicating that HTRA1 prevents the conversion of α-synuclein monomers to amyloid fibrils, dissociates α-synuclein amyloid fibrils, and interferes with seeding (25).

Time-resolved mass spectrometry analysis demonstrates that the proteolytic degradation of tau fibrils by HTRA1 is initiated in regions surrounding the β-sheet core, followed by multiple cuts within the core region. This finding suggests a mechanism by which HTRA1 targets aggregation-prone conformations, which can be explained by the induced fit mechanism of proteolysis. This model is supported by the greater activation of the proteolytic activity by tau fibrils compared to unstructured soluble tau. In general, substrates bind to the active site of proteases in an extended β-strand conformation, which has also been shown for HtrA proteases (26, 27). The linear extension of a substrate optimizes its interaction with the substrate-binding pockets and optimally exposes the main chain amide atoms to the catalytic residue of the protease. However, the tight packing and extensive intermolecular hydrogen bonding of the β-sheet structures of the fibril core make it less favorable for interaction with the active site. Therefore, proteolysis by HTRA1 is preceded by fibril dissociation before extracted individual peptides can interact productively with the active site of the protease (10). Regarding the differences and similarities between the fibrillar and the soluble substrates, we find a greater number of peptides and cleavage sites when fibrillar tau is proteolyzed. This finding can be explained by the better binding of preformed β-strands in tau fragments derived from fibrillar structures. However, the frequency of cuts is lower in fibrillar structures, which may be explained by the need to extract individual peptides from the fibrils, which is expected to slow proteolysis. In addition, the comparison of preferences for residues surrounding the scissile bond shows only minor differences between soluble and fibrillar tau, which is consistent with the substrate binding pockets of the active site of HTRA1 remaining the same.

We also show that HTRA1 interferes with seeded tau fibril formation in sedimentation assays and TEM analyses. Although the concentrations of HTRA1 used in these *in vitro* reconstitution assays are higher than those expected in tissue, such conditions are standard for purified biochemical systems to demonstrate catalytic capacity against resistant substrates. Importantly, the relevant comparison is not the bulk stoichiometry of protease *versus* tau monomers, but the interaction of trimeric HTRA1 with the dense array of cleavage sites presented by tau fibrils at a much higher concentration than the tau monomer. Given that HTRA1 avidly accumulates at fibril surfaces (10, 11, 13), the local effective concentration of HTRA1 *in vivo* is likely to be much higher than the free concentration. When combined with our live-cell imaging data showing direct HTRA1–tau fibril interaction and degradation, these findings support the physiological relevance of HTRA1-mediated fibril clearance.

In addition to HTRA1, numerous other proteases have been shown to cleave the tau protein, but the majority of the identified cleavage sites of 17 proteases were located outside of the β-sheet-forming regions, except for residue I308, which is targeted by the chymotrypsin-like activity of the 20S proteasome, and residue D314, which is targeted by caspase 2 (28). It is therefore not surprising that numerous tau fragments produced by a variety of proteases, targeting proteolytic sites located outside the microtubule-binding repeats and thus the β-sheet-forming regions, have been shown to mediate oligomer formation, aggregation, and toxicity in cells or have been identified in patient brain samples and are therefore suspected to be involved in tauopathies (29, 30).

As shown here and previously, HTRA1 is taken up from the medium to enter cells that do not produce HTRA1 themselves to perform protein quality control (10, 31). It is therefore conceivable that secreted HTRA1 can diffuse through tissue. Its uptake into acceptor cells with compromised proteostasis will lower the levels of tau fibrils, thus reducing the ability of the pathogenic tau conformation to spread. Interestingly, the production of HTRA1 by healthy non-neuronal cells that do not harbor tau fibrils enables non-autonomous proteostasis in neuronal cells. The concept of cell non-autonomous proteostasis was first established and is most widely studied in non-vertebrates, but at least some aspects seem to be conserved in mammals (32, 33). Therefore, transcellular chaperone and protease signaling may be an attractive direction for future research. In the case of HTRA1, transcellular signaling could modulate HTRA1 expression through activation of specific transcription factors or epigenetic mechanisms, as the HTRA1 promoter contains a CpG island (34).

Our data highlight the therapeutic potential of HTRA1 due to its extracellular and intracellular location and its accumulation at sites of tau and Aβ fibril deposition (13). We further suggest the potential of boosting HTRA1 production in healthy cells to safeguard cells that are compromised by protein aggregates. Consistently, HTRA1 levels are elevated in AD patient samples and tau transgenic mouse models (12, 35, 36, 37, 38). In addition, HTRA1 has previously been shown to degrade Aβ oligomers and APOE4, the latter being a risk factor for late-onset AD (13, 39). As observed with tau, HTRA1 levels were elevated in Aβ-associated mouse models (40, 41). In addition, a large proteomic study of 610 brain tissues from individuals with clinical diagnoses of no cognitive impairment, mild cognitive impairment, and AD dementia found elevated HTRA1 levels in *APOE4* carriers (42). Our data underscore that the identification and characterization of evolutionarily conserved protein quality control factors such as HTRA1 warrant further efforts to translate the mechanistic insights obtained from basic research into therapeutic strategies.

## Experimental procedures

### Plasmids

Bacterial expression plasmids for the purification of 4R2N tau, as well as Strep-tagged HTRA1 and HTRA1^S328A^ lacking the N-terminal mac domain, were published previously (10). Plasmids containing an mCherry sequence were generated using published constructs (43).

### Purification of recombinant HTRA1 and tau

Recombinant 4R2N tau (441-residue isoform) as well as Strep-tagged HTRA1 and HTRA1^S328A^ (residues 158–480) were purified as described (10).

### Heparin-induced fibril formation of recombinant tau, generation of tau seeds, and effects of HTRA1

Fibril formation was done as described (10). Tau seeds were prepared by sonication of preformed heparin-induced recombinant fibrils using an ultrasonic homogenizer (Bandelin) (3 x 20 s, volumes varied between 50 and 150 μl and samples were cooled on ice between sonications). Seeds can be snap-frozen in liquid nitrogen and stored at −80 ^°^C. To study the effects of HTRA1, HTRA1^S328A^ was pre-incubated with seeds for 3 h, while HTRA1 was directly added to tau and seeds at concentrations indicated. As controls, tau, seeds, HTRA1 and HTRA1^S328A^ were incubated alone. Fibril formation was analyzed after 3 days of incubation by sedimentation assays as described (10). Protein levels of tau were determined by densitometry analysis using Image J (44). For transmission electron microscopy of fibrils, samples were diluted, subjected to a formvar-coated copper grid and incubated for 60 s. After removal of excess liquid, the grids were incubated with a staining solution of 0.75% uranyl formate, 6 mM NaOH, and dried at room temperature. The samples were analyzed with a JEOL JEM 1400 PLUS at 120 kV. At least 25 images (7.89 μm × 7.89 μm) were taken per condition. Quantification of the total fibril length per image was performed using the ridge detection plugin of Image J (44). The kinetics of fibril formation were investigated by measuring ThT fluorescence after 4, 24, 48, and 72 h of incubation.

### HTRA1 activity assay

The specific activity of recombinant HTRA1 in the presence of tau species was measured using a synthetic HTRA1 substrate consisting of para-nitroanilin (pNA) coupled to the C-terminus of the peptide VFNTLPMMGKASPV (16). HTRA1 (1 μM) was mixed with the indicated concentration of tau species in a final volume of 100 μl in 100 mM HEPES 100 mM NaCl pH 7.5. After incubation for 1 min at 37 °C and 700 rpm, 500 μM of the pNA substrate was added. Substrate cleavage was monitored continuously for 2 h at a wavelength of 405 nm using a SpectraMax iD5 spectrophotometer. The specific enzyme activities of HTRA1 were derived from at least three independent measurements and calculated using the following formula: $Specific\;enzyme\;activity=\frac{{\Delta A}_{405}\times V}{m\times \epsilon \times t}$

ΔA_405_: change of absorption at λ = 405 nm.

V: reaction volume (ml)

m: amount of protease (mg)

ε: molar extinction coefficient of pNA (M^−1^ × cm^−1^)

t: time (min)

### Crosslinking mass spectrometry

5 μM tau fibril seeds and 5 μM HTRA1S328 A were preincubated in 100 mM HEPES, 100 mM NaCl, pH 7.5, for 15 min at 37°C to allow binding. Subsequently, 2 mM (final concentration) of the cross-linking agent PhoX was added. After 15 min, the reaction was stopped by quenching with Tris-HCl. Samples were trypsinized and prepared for mass spectrometry. For peptide identification, the RAW files were loaded into Proteome Discoverer (version 2.5.0.400, Thermo Scientific). All MS/MS spectra were searched using MSAmanda v2.0.0.16129 (45). The RAW files were first searched against the databases ID1416.fasta (2 sequences; 782 residues), tags_v11.fasta (28 sequences; 2153 residues), uniprot_reference_E-coli_k12_2023-09 to 19.fasta (4362 sequences; 1 354 446 residues) and PD_Contaminants_TAGs_v20_tagsremoved.fasta, using the following search parameters: Peptide mass tolerance was set to ±10 ppm and the fragment mass tolerance to ±10 ppm. The maximum number of missed cleavages was set to 2. The result was filtered to 1% FDR at the protein level using the Percolator algorithm integrated in Thermo Proteome Discoverer. In the second step, the RAW files were searched against the created sub-databases termed ID1416.fasta (Tau and HTRA1 sequences; 782 residues), using the following search parameters: iodoacetamide derivative on cysteine was set as a fixed modification, oxidation on methionine, phosphorylation on serine, threonine, and tyrosine, deamidation on asparagine and glutamine, PhoX on lysine, pyro-glu from q on peptide N-terminal glutamine, and acetylation on protein N-terminus were set as variable modifications.

Monoisotopic masses were searched within unrestricted protein masses for tryptic specificity. The peptide mass tolerance was set to ±10 ppm and the fragment mass tolerance to ±10 ppm. The maximum number of missed cleavages was set to 2. The result was filtered to 1% FDR at the protein level using Percolator algorithm integrated in Thermo Proteome Discoverer. Additional high-quality filtering by setting a minimum MS Amanda Score of 150 on PSM level was applied. Protein areas were quantified using IMP-apQuant (46) by summing unique and razor peptides and applying intensity-based absolute quantification (47) with subsequent normalization based on the MaxLFQ algorithm (48). Proteins were filtered to be identified by at least 2 PSMs in at least one sample.

### Time-resolved proteolysis and analysis by mass spectrometry

For proteolysis, 10 μM of soluble or fibrillar tau protein was mixed with 2 μM of HTRA1 in 100 mM HEPES 100 mM NaCl pH 7.5 and incubated at 37 °C and 350 rpm. Samples (20 μl) were taken at the indicated time points and added directly to 120 μl ice-cold acetone for overnight precipitation at −80 °C. Precipitated proteins were removed by centrifugation (15,000 rpm, 1 h, 4 °C), and the peptide-containing supernatant was lyophilized at 30 °C in a SpeedVac concentrator. Dried samples were stored at −80 °C.

### LC/MS/MS

Experiments were performed on an Orbitrap Elite or Fusion Lumos mass spectrometer (Thermo Fischer Scientific) that was coupled to an Evosep One liquid chromatography (LC) system (Evosep Biosystems, Odense, Denmark). Analysis on the Evosep One was performed on a commercially available EV-1064 Analytical Column – 60 & 100 samples/day (Length (LC) 8 cm; ID 100 μm; OD 360 mm; emitter EV-1086 Stainless steel emitter). The LC system was equipped with two mobile phases: solvent A (0.1% formic acid, FA, in water) and solvent B (0.1% FA in acetonitrile). All solvents were of ultra-high-performance liquid chromatography grade (Honeywell). For analysis on the Evosep One, samples were first loaded onto Evotips according to the manufacturer's guidelines. For peptide separation, we used the 60 samples per day gradient which has an effective gradient of 21 min.

The mass spectrometers were operated using Xcalibur software (Elite: v2.2 SP1.48 or Lumos: v4.5.445.18). The mass spectrometers were set in the positive ion mode. Precursor ion scanning (MS1) was performed in the Orbitrap analyzer (Fourier Transform Mass Spectrometry with the internal lock mass option turned on (lock mass was 445.120025 m/z, polysiloxane) (49). MS2 Product ion spectra were recorded only from ions with a charge greater than +1 and in a data dependent fashion in the Ion Trap Mass Spectrometry. All relevant MS settings (resolution, scan range, AGC, ion acquisition time, charge state isolation window, fragmentation type and details, cycle time, number of scans performed, and various other settings) for the individual experiments can be found in Data S6.

### Peptide and protein identification using MaxQuant

RAW spectra were submitted to an Andromeda search (50) in MaxQuant (version 2.0.2.0 or 2.0.3.0) using the default settings (51). Label-free quantification (LFQ) (48) and match between runs was enabled. Normalization in MaxQuant was disabled. MS/MS spectra data for Project ACE_0711 were searched against the custom database ACE_0699_UP000000625_83333.fasta (4450 entries). For project ACE_0809 MS/MS spectra data were searched against the custom database ACE_0809_SOI_v01.fasta (5 entries, containing sequences of interest) and the contaminant database as implemented in MaxQuant. Andromeda searches allowed oxidation of methionine residues (16 Da) and acetylation of the protein N-terminus (42 Da). No static modifications were specified. Enzyme specificity was set to “unspecific”. The instrument type for Andromeda searches was set to Orbitrap and the precursor mass tolerance was set to ±20 ppm (first search) and ±4.5 ppm (main search). The MS/MS match tolerance was set to ±20 ppm. The peptide spectrum match FDR and the protein FDR were set to 0.01 (based on target-decoy approach). The minimum peptide length was 7 amino acids. Unique and razor peptides were allowed for protein quantification. In addition to unmodified peptides, modified peptides with dynamic modifications were allowed for quantification. The minimum score for modified peptides was set to 40.

### UMSAP

MS-data were evaluated with the Targeted Proteolysis module of UMSAP 2.2.1 (20). MS-data of the tau alone samples served as reference for all UMSAP calculations. The significance level was set to 0.05 and the minimum score value to 50. A log2 transformation was applied to the data before the analysis. The amino acid distribution around the cleavage sites included five residues in each direction. The amino acid sequence used for this calculation is shown in Data S2.

### Calculation of the relative frequency of cuts by UMSAP

The calculation of the cut frequency is done in 2 steps. First, UMSAP groups all MS-detected peptides that share the same P1-P1′ bond and the average intensities are calculated for each peptide in each experiment. Average intensity ratios are then calculated for each peptide, taking as a reference the first average intensity >0 along the time points for each peptide. The relative frequency of cuts for a P1 site at a given time point is then calculated by summing the average intensity ratios of all peptides sharing the same P1 site.

If the peptide was not detected at a time point or the intensity values are not significantly different from the control experiments, the average intensity for that time point is set to zero. A numerical example is given in Data S7.

### Cell lines and culture media

SH-SY5Y cells were purchased from American Type Culture Collection (CRL-3216) and tested for *mycoplasma* before use. SH-SY5Y cells were cultivated in a 1:1 mixture of DMEM and Ham's F-12 Medium. Both media were supplemented with 10% fetal calf serum and 1% penicillin/streptomycin unless described otherwise. For live cell imaging experiments, medium lacking phenol red was applied. The generation of Flp-In T-REx expression cells was performed as described by Thermo Fisher Scientific. For experiments performed with Flp-In T-Rex cells tet system approved serum (Takara Bio) was used.

### Protein labeling

Recombinant HTRA1^S328A^ was labeled with the amine-reactive DyLight 633 NHS Ester, while fibrils made of recombinant 4R2N tau were labeled with DyLight 488 NHS Ester (used for the treatment of SH-SY5Y cells). The labeling reaction was performed as described (10).

### Live cell imaging of SH-SY5Y cells treated with recombinant tau and HTRA1

For localization of internalized HTRA1 in cells containing tau seeds (Figs. 4, *A*–*E* and S4, *A*–*H*), 8 × 10^4^ *Mycoplasma*-free SH-SY5Y (ATCC CRL-2266) cells, which were pretreated with CellTracker Deep Red (Thermofisher, C34565), were plated onto μ-slide 8-well plates (IBIDI). On the following day, 300 nM DyLight 488 labeled tau seeds or DPBS were added to cells. After 24 h, cells were washed and treated with 300 μl conditioned medium containing 1% serum and HTRA1^S328A^-mCherry or secreted mCherry, respectively, followed by live cell microscopy. Tau seeding in the presence of HTRA1 was investigated by treating cells with 300 μl conditioned medium containing 1% serum and HTRA1^S328A^-mCherry or secreted mCherry. After 24 h, fresh DMEM imaging medium with 1% FCS and 300 nM DyLight 488 labeled tau seeds or DPBS were added to cells. Note that morphological changes of the cells and cytotoxic effects were observed at longer imaging times, most likely due to starvation in media containing 1% FCS.

Live cell analysis of purified, recombinant and proteolytically active or inactive HTRA1 and recombinant tau (Figs. 4, *F* and *G* and S4, *I*–*L*) was performed as described above, with some modifications such as the use of ”Cell Tracker Orange" (Thermofisher, C34551). For HTRA1 internalization, cells were pretreated with 150 nM labeled tau seeds. After 24 h, cells were treated with medium containing 50 μg/ml labeled HTRA1 or HTRA1S328 A, respectively. For internalization of tau seeds, cells were first treated with medium containing 50 μg/ml labeled HTRA1 or HTRA1S328A, respectively. After 24 h, fresh imaging medium with 150 nM labeled tau seeds or PBS were added to cells. In each case, cell positions were determined before the addition of either HTRA1 or tau seeds, and live cell microscopy was performed immediately after the addition of either HTRA1 or tau seeds.

Live-cell timelapse confocal spinning disk microscopy was performed using an inverted Nikon Eclipse TiE microscope system (Nikon Europe B.V.), equipped with a CSU-X1 spinning disk unit (Yokogawa). Images were acquired using a CFI Plan Apo 40×/0.95 NA dry objective (Nikon).

For experiments involving secreted HTRA1^S328A^-mCherry, imaging utilized an Andor REVOLUTION 500 AOTF Laser Combiner with diode laser lines cw 640 nm (100 mW) for CellTracker Deep Red, 561 nm (50 mW) for mCherry, and 488 nm (50 mW) for DyLight 488. In experiments with recombinant HTRA1^S328A^ and WT, excitation was provided by an Andor 4-line Integrated Laser Engine (ILE) with diode laser lines used for excitation diode laser cw 637 nm (140 mW) for DyLight 633, 561 nm (50 mW) for CellTracker Orange, and 488 nm (50 mW) for DyLight 488 was used. Detection was performed using either an iXon3 897 ection EMCCD (for secreted HTRA1) or a ZL41 Cell 4.2 sCMOS camera (for recombinant HTRA1) (Andor Technology). Emission filters included single-band pass Cy5 (BP 685/40 nm), RFP-1 (617/73 nm, for secreted HTRA1) or RFP-2 (BP 593/40 nm, for recombinant HTRA1), and dual-band pass filter EGFP/Cy5 (BP 538/50 nm + BP 685/45 nm). Image Acquisition was controlled using Andor IQ software (Andor Technology).

Images were acquired at 10 random positions per condition, with a time-lapse interval of 5 min for the first 2 h and 15 min for the subsequent 28 h. Nikons hardware-based autofocus system PFS (Nikon Europe B. V.) was used to maintain focus stability and prevent drift. All live-cell imaging was conducted at 37 °C with 5% CO_2_ in imaging medium.

### Image analysis

Automated image analysis was performed with CellProfiler (version 4.2.6) (52). For analysis of live-cell imaging data represented in Figures 4, *A*–*E* and S4, *A*–*H*, cells were detected using the Run Cellpose plugin for CellProfiler using the cyto2 model (expected object size 100 px, cell probability threshold −3 and flow threshold 0.8) with Omnipose for mask reconstruction (53, 54) on the rescaled (full intensity range) and smoothed (median filter artifact diameter 2) CellTracker Deep Red image. The morphological challenges of SH-SY5Y cells, such as their partially overlapping spindle shape, can make it difficult to reliably define individual cells and cell boundaries. Therefore, we used the entire area covered by cells instead of quantifying on a per-cell basis. Thus, we identified regions covered by cells using the CellTracker Deep Red whole cell stain and combined them to form a single entity (total cell area) and detected discrete fluorescent spots within this region. To account for variations in cellular coverage over time within the image area, we normalized the number of spots by the measured cell area (number of spots/μm^2^) and multiplied this factor by 300, which reflects the average cell area of an individual SH-SY5Y cell. To detect discrete fluorescent Tau seed spots that co-localize with HTRA1S328A-mCherry, the segmented Tau spots were masked for HTRA1 detected spots. Only spots with a minimal overlap of 40% were considered double-positive (HTRA1-positive Tau spots). The same criteria were applied for the opposite combination (Tau-positive HTRA1 spots). To enhance vesicular structures of both Tau seeds and HTRA1 spots, difference of Gaussians (DoG) was applied to the respective channels (Gaussian filters of size 2 and 8). Discrete spots of Tau and/or HTRA1 signal were detected and related to the respective total cell area. The MaskObjects module was used to detect colocalized spots if their overlap was at least 40%. Data extraction, analysis and graph plotting were conducted using Microsoft Office Excel and GraphPad Prism 10.

For the data shown in Figures 4, *F* and *G* and S4, *I*–*L*, the same pipeline was used with minor adjustments for the different dyes and the modified microscope setup. Cells were detected using the Run Cellpose plugin with the cyto2 model (expected object size 120 px, cell probability threshold −0.5 and flow threshold 0.8) with Omnipose for mask reconstruction (62, 63) on the rescaled (full intensity range) and smoothed (median filter artifact diameter 4) CellTracker Orange image. Intensity measurements were performed over the entire cell area (merged cells). Discrete Tau and HTRA1 spots were detected in the DyLight488 and DyLight633 channels, respectively, after applying a DoG (Gaussian filters of size 1 and 4).

### Data availability

All data are contained within the manuscript, except the mass spectrometry data have been deposited to the ProteomeXchange Consortium *via* the PRIDE (55) partner repository (https://www.ebi.ac.uk/pride/archive/) with the dataset identifier PXD052323.

## Supporting information

This article contains supporting information.

## Conflict of interest

The authors declare that they do not have any conflicts of interest with the content of this article.
