# Supplementary material for: The serine protease HTRA1 targets tau fibrils and provides a proteolytic barrier against pathogenic protein conformations
Source: J Biol Chem. 2025 Sep 16;301(10):110729. doi: 10.1016/j.jbc.2025.110729 (PMC12547462; doi:10.1016/j.jbc.2025.110729)
Supplement: Supporting information [file mmc1.pdf]

The serine protease HTRA1 targets Tau fibrils and provides a proteolytic barrier against pathogenic protein conformations

Birte Hagemeier, Kamilla Ripkens, Nina Schulze, Anika Bluemke, Michal Strzala, Michelle Koci, Farnusch Kaschani, Markus Kaiser, Michael Erkelenz, Sebastian Schluecker, Melisa Merdanovic, Simon Poepsel, Doris Hellerschmied, Steven G.

Burston and Michael Ehrmann

Supporting Information

Supporting Figures S1-S4

Supporting data 1
Crosslinking mass spec data HTRA1 and tau fibrils

Supporting data 2
Peptides identified in soluble tau digested by HTRA1

Supporting data 3
Multiple sequence alignment of Tau proteins

Supporting data 4
Peptides identified in tau fibrils digested by HTRA1

Supporting data 5
Peptides identified in tau CN fibrils digested by HTRA1

Supporting data 6
Mass spectrometry information

Supporting data 7
Example for how UMSAP calculates the relative frequency of cuts

Supporting Tables S1-S3

Table S1. P1 residues in tau with the highest relative number of cuts

Table S2. P1 residues in tau with the second highest relative number of cuts

Table S3. P1 residues that are differentially cleaved in soluble and fibrillar tau at the 120 min time point

Supporting Videos

Movie S1, Cyto tau fibrils (green), extracell HTRA1S328A (red)

Movie S2, Cyto HTRA1S328A (red), extracell tau fibrils (green)

Movie S3 Tau + HTRA1

Movie S4 PBS-Control only Tau

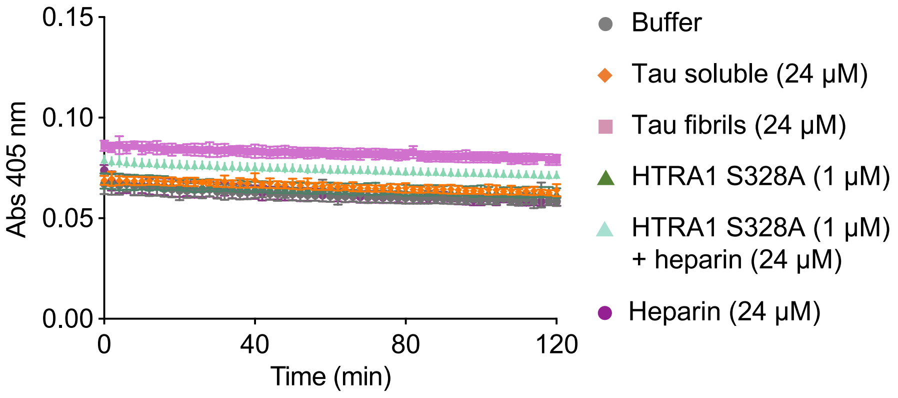

Fig. S1. Progress curves of control reactions relevant to Fig 1A. 500 µM of the VFNTLPMMGKASPV-pNA substrate was mixed with: buffer; 24 µM soluble tau; 24 µM tau fibrils; 1 µM (monomer-equivalent) inactive active site HTRA1 S328A variant with or without 24 µM heparin; and 24 µM of heparin. The respective reactions were continuously measured at 405 nm over a 2 h period. No increase in absorbance at 405 nm was observed, confirming the absence of proteolytic activity in the samples. Error bars indicate the standard deviation(n=3).

Fig. S2A Proteolysis of soluble tau by HTRA1

	1	2	3	4	5	6	7	8	9	10	11	12	13	14	15	16	17	18	19	20	21	22	23	24	25	26	27	28	29	30	31	32	33	34	35	36	37	38	39	40	41	42	43	44	45	46	47	48	49	50	Residue no.
	M	A	E	P	R	Q	E	F	E	V	M	E	D	H	A	G	T	Y	G	L	G	D	R	K	D	Q	G	G	Y	T	M	H	Q	D	Q	E	G	D	T	D	A	G	L	K	E	S	P	L	Q	T	Sequence
Min	*	*	:	*	:	*	*	*	:	.		*	*	:	.													.	*				*	*	:	*	*	*		:		*	*	*	*	*	*		*		Conservation
0.25	0									0	0																																									Rel. freq. cuts
1	0									0	0																																									
2	1									1	0																																									
5	1									0	1																																									
10	0									0	0																																									
120	2									1	1																																									

	51	52	53	54	55	56	57	58	59	60	61	62	63	64	65	66	67	68	69	70	71	72	73	74	75	76	77	78	79	80	81	82	83	84	85	86	87	88	89	90	91	92	93	94	95	96	97	98	99	100	Residue no.
	P	T	E	D	G	S	E	E	P	G	S	E	T	S	D	A	K	S	T	P	T	A	E	D	V	T	A	P	L	V	D	E	G	A	P	G	K	Q	A	A	A	Q	P	H	T	E	I	P	E	G	Sequence
Min	*	:	:	*	*	:	*	*		*	*	*	*	*	*	*	*	*	*	*	*	*	*																											Conservation
0.25																																																		Rel. freq. cuts
1																																																		
2																																																		
5																																																		
10																																																		
120																																																		

	101	102	103	104	105	106	107	108	109	110	111	112	113	114	115	116	117	118	119	120	121	122	123	124	125	126	127	128	129	130	131	132	133	134	135	136	137	138	139	140	141	142	143	144	145	146	147	148	149	150	Residue no.
	T	T	A	E	E	A	G	I	G	D	T	P	S	L	E	D	E	A	A	G	H	V	T	Q	A	R	M	V	S	K	S	K	D	G	T	G	S	D	D	K	K	A	K	G	A	D	G	K	T	K	Sequence
Min			*	*	*	*	*	*	*	*	*		.		*	*	:	*	*	*	*	*	*	*	*			.			.	*	*		*	*		*	:					.	.	.			:	*	Conservation
0.25						0																			0																										Rel. freq. cuts
1						0																			0																										
2						0																			0																										
5						0																			0																										
10						0																			0																										
120						1																			1																										

	151	152	153	154	155	156	157	158	159	160	161	162	163	164	165	166	167	168	169	170	171	172	173	174	175	176	177	178	179	180	181	182	183	184	185	186	187	188	189	190	191	192	193	194	195	196	197	198	199	200	Residue no.
	I	A	T	P	R	G	A	A	P	P	G	Q	K	G	Q	A	N	A	T	R	I	P	A	K	T	P	P	A	P	K	T	P	P	S	S	G	E	P	P	K	S	G	D	R	S	G	Y	S	S	P	Sequence
Min	*	*	*	*	*	*	*	*		*		*	*	*		:	*	*	*	*	*	*	*	*	*		*	:	*	*	*		*			*	*			*	*	*	:	*	*	*	*	*	*	*	Conservation
0.25																																																		Rel. freq. cuts
1																																																		
2																																																		
5																																																		
10																																																		
120																																																		

	201	202	203	204	205	206	207	208	209	210	211	212	213	214	215	216	217	218	219	220	221	222	223	224	225	226	227	228	229	230	231	232	233	234	235	236	237	238	239	240	241	242	243	244	245	246	247	248	249	250	Residue no.
	G	S	P	G	T	P	G	S	R	S	R	T	P	S	L	P	T	P	P	T	R	E	P	K	K	V	A	V	V	R	T	P	P	K	S	P	S	S	A	K	S	R	L	Q	T	A	P	V	P	M	Sequence
Min	*	*	*	*	*	*	*	*	*	*	*	*	*	*	*	*	*	*	*	:	*	*	*	*	*	*	*	*	*	*	*	*	*	*	*	*	*	:	:	*	*	*	*	*	:	*	*		*	*	Conservation
0.25																									1		0	0							0		0		0		0	1							Rel. freq. cuts
1																									1		0	0							0		0		0		0	3							
2																									3		0	0							0		0		0		0	4							
5																									2		0	0							0		0		0		0	3							
10																									3		0	1							0		0		0		0	4							
120																									5		1	3							1		1		1		1	7							

	251	252	253	254	255	256	257	258	259	260	261	262	263	264	265	266	267	268	269	270	271	272	273	274	275	276	277	278	279	280	281	282	283	284	285	286	287	288	289	290	291	292	293	294	295	296	297	298	299	300	Residue no.
	P	D	L	K	N	V	K	S	K	I	G	S	T	E	N	L	K	H	Q	P	G	G	G	K	V	Q	I	I	N	K	K	L	D	L	S	N	V	Q	S	K	C	G	S	K	D	N	I	K	H	V	Sequence
Min	*	*	*	*	*	*	:	*	*	*	*	*	*	*	*	*	*	*	*	*	*	*	*	*	*	*	*	*	*	*	*	*	*	*	*	*	*	*	*	*	*	*	*	*	*	*	*	*	*	*	Conservation
0.25						3						0			1									0		1	1		1		1		0	0		0		0		4				0						Rel. freq. cuts
1						5						0			1									0		2	1		1		3		0	0		3		0		4				0						
2						6						0			1									0		5	3		3		4		0	0		5		3		9				0						
5						6						0			5									1		5	5		2		4		1	1		7		0		10				0						
10						8						0			5									2		8	6		3		4		1	1		7		4		15				1						
120						22						1			11									6		13	12		7		6		2	2		13		10		24				2						

	301	302	303	304	305	306	307	308	309	310	311	312	313	314	315	316	317	318	319	320	321	322	323	324	325	326	327	328	329	330	331	332	333	334	335	336	337	338	339	340	341	342	343	344	345	346	347	348	349	350	Residue no.
	P	G	G	G	S	V	Q	I	V	Y	K	P	V	D	L	S	K	V	T	S	K	C	G	S	L	G	N	I	H	H	K	P	G	G	G	Q	V	E	V	K	S	E	K	L	D	F	K	D	R	V	Sequence
Min	*	*	*	*	*	*	:	*	*	*	*	*	*	*	*	*	*	*	*	*	*	*	*	*	*	*	*	*	*	*	*	*	*	*	*	*	*	*	*	*	*	*	*	*	*	*	*	*	*	*	Conservation
0.25		0				0	0	4	0	1	0		0	0	0	0	0	1	0		0	3	0	0	0	0	0	0	0	0				0	0		0		0		0	0	0	2	0					0	Rel. freq. cuts
1		1				0	0	8	1	0	0		2	0	0	1	0	2	1		0	5	0	0	0	0	0	2	0	0				0	0		0		0		0	0	0	2	0					1	
2		1				0	0	17	4	0	0		4	0	0	2	0	3	2		1	11	0	0	1	0	0	5	0	0				0	0		0		2		0	0	0	6	0					0	
5		2				0	0	17	4	0	0		4	0	0	2	0	5	2		1	10	0	0	1	0	0	5	1	0				0	0		0		1		0	0	0	3	0					2	
10		2				0	0	21	5	0	1		5	0	0	2	0	5	2		1	14	1	0	1	0	0	6	1	0				0	0		0		2		1	0	0	5	0					2	
120		4				2	1	42	9	2	3		8	1	1	3	1	9	3		2	32	1	2	5	1	1	13	1	1				1	1		1		3		2	1	1	15	1					2	

	351	352	353	354	355	356	357	358	359	360	361	362	363	364	365	366	367	368	369	370	371	372	373	374	375	376	377	378	379	380	381	382	383	384	385	386	387	388	389	390	391	392	393	394	395	396	397	398	399	400	Residue no.
	Q	S	K	I	G	S	L	D	N	I	T	H	V	P	G	G	G	N	K	K	I	E	T	H	K	L	T	F	R	E	N	A	K	A	K	T	D	H	G	A	E	I	V	Y	K	S	P	V	V	S	Sequence
Min	*	*	*	*	*	*	*	*	*	*	*	*	*	*	*	*	*	:	*	*	*	*	*	*	*	*	*	*	*	*	*	*	*	*	*	*	*	*	*	*	*	*	*	*	*	*	*	*	*	*	Conservation
0.25							0			2	0		0	0	0	0										5	0											0	0		0		12	1	1	1			0	0		Rel. freq. cuts
1							0			4	0		0	0	0	0										6	0											0	0		0		24	1	1	1			0	0		
2							0			4	0		0	0	2	0										9	0											1	0		0		39	3	1	0			2	0		
5							0			5	0		0	0	0	0										8	0											0	0		0		44	2	1	2			1	1		
10							1			5	0		1	0	1	0										12	1											1	1		0		47	3	1	1			3	1		
120							3			6	1		2	1	3	1										27	2											2	2		1		51	4	1	2			6	1		

	401	402	403	404	405	406	407	408	409	410	411	412	413	414	415	416	417	418	419	420	421	422	423	424	425	426	427	428	429	430	431	432	433	434	435	436	437	438	439	440	441										Residue no.
	G	D	T	S	P	R	H	L	S	N	V	S	S	T	G	S	I	D	M	V	D	S	P	Q	L	A	T	L	A	D	E	V	S	A	S	L	A	K	Q	G	L										Sequence
Min	*	*	*	*	*	*	*	*	*	*	*	*	*	*	*	*	*	*	*	*	*	*	*	*	*	*	*	*	*	*	*	*	*	*	*	*	*	*	*	*	*										Conservation
0.25	1	0	0	0	0	0	0	7	1	1	1	1	0	0	1	0		0	1	0	1	0	0	0	3	2	1	3		0	1	2		1					0	0											Rel. freq. cuts
1	1	1	1	1	0	1	1	15	2	2	4	2	0	1	1	0		1	2	2	2	1	2	2	4	3	1	4		0	0	4		2					0	0											
2	1	2	2	2	2	2	2	28	2	2	10	2	1	5	2	1		1	3	3	3	2	3	2	4	8	4	10		2	2	8		1					1	1											
5	1	1	1	2	1	2	1	22	2	2	8	2	1	2	2	1		2	3	3	3	2	4	4	5	6	2	5		1	1	6		2					0	0											
10	1	2	2	2	1	2	2	28	2	2	12	2	1	4	2	2		2	3	4	3	2	4	3	5	7	4	7		2	2	8		3					1	0											
120	2	2	2	2	0	3	2	29	3	3	16	2	1	9	3	2		2	3	5	5	3	5	4	6	9	2	13		3	2	12		3					1	1											

Fig. S2B Proteolysis of fibrillar tau by HTRA1

	1	2	3	4	5	6	7	8	9	10	11	12	13	14	15	16	17	18	19	20	21	22	23	24	25	26	27	28	29	30	31	32	33	34	35	36	37	38	39	40	41	42	43	44	45	46	47	48	49	50	Residue no.
	M	A	E	P	R	Q	E	F	E	V	M	E	D	H	A	G	T	Y	G	L	G	D	R	K	D	Q	G	G	Y	T	M	H	Q	D	Q	E	G	D	T	D	A	G	L	K	E	S	P	L	Q	T	Sequence
Min	*	*	:	*	:	*	*	*	:	.		*	*	:	.													.	*				*	*	:	*	*	*		:		*	*	*	*	*	*		*		Conservation
0.25	0																			0												0																			Rel. freq. cuts
1	0																			0												0																			
2	0																			0												0																			
5	1																			0												1																			
10	2																			1												1																			
120	0																			0												0																			

	51	52	53	54	55	56	57	58	59	60	61	62	63	64	65	66	67	68	69	70	71	72	73	74	75	76	77	78	79	80	81	82	83	84	85	86	87	88	89	90	91	92	93	94	95	96	97	98	99	100	Residue no.
	P	T	E	D	G	S	E	E	P	G	S	E	T	S	D	A	K	S	T	P	T	A	E	D	V	T	A	P	L	V	D	E	G	A	P	G	K	Q	A	A	A	Q	P	H	T	E	I	P	E	G	Sequence
Min	*	:	:	*	*	:	*	*		*	*	*	*	*	*	*	*	*	*	*	*	*	*																												Conservation
0.25																																																			Rel. freq. cuts
1																																																			
2																																																			
5																																																			
10																																																			
120																																																			

	101	102	103	104	105	106	107	108	109	110	111	112	113	114	115	116	117	118	119	120	121	122	123	124	125	126	127	128	129	130	131	132	133	134	135	136	137	138	139	140	141	142	143	144	145	146	147	148	149	150	Residue no.
	T	T	A	E	E	A	G	I	G	D	T	P	S	L	E	D	E	A	A	G	H	V	T	Q	A	R	M	V	S	K	S	K	D	G	T	G	S	D	D	K	K	A	K	G	A	D	G	K	T	K	Sequence
Min			*	*	*	*	*	*	*	*	*		.		*	*	:	*	*	*	*	*	*	*	*			.			.	*	*		*	*		*	:					.	.	.			:	*	Conservation
0.25						0						0													0																										Rel. freq. cuts
1						0						0													0																										
2						0						0													0																										
5						1						0													1																										
10						2						0													2																										
120						2						1													3																										

	151	152	153	154	155	156	157	158	159	160	161	162	163	164	165	166	167	168	169	170	171	172	173	174	175	176	177	178	179	180	181	182	183	184	185	186	187	188	189	190	191	192	193	194	195	196	197	198	199	200	Residue no.
	I	A	T	P	R	G	A	A	P	P	G	Q	K	G	Q	A	N	A	T	R	I	P	A	K	T	P	P	A	P	K	T	P	P	S	S	G	E	P	P	K	S	G	D	R	S	G	Y	S	S	P	Sequence
Min	*	*	*	*	*	*	*	*		*		*	*	*		:	*	*	*	*	*	*	*	*	*		*	:	*	*	*		*			*	*			*	*	*	:	*	*	*	*	*	*	*	Conservation
0.25																																																			Rel. freq. cuts
1																																																			
2																																																			
5																																																			
10																																																			
120																																																			

	201	202	203	204	205	206	207	208	209	210	211	212	213	214	215	216	217	218	219	220	221	222	223	224	225	226	227	228	229	230	231	232	233	234	235	236	237	238	239	240	241	242	243	244	245	246	247	248	249	250	Residue no.
	G	S	P	G	T	P	G	S	R	S	R	T	P	S	L	P	T	P	P	T	R	E	P	K	K	V	A	V	V	R	T	P	P	K	S	P	S	S	A	K	S	R	L	Q	T	A	P	V	P	M	Sequence
Min	*	*	*	*	*	*	*	*	*	*	*	*	*	*	*	*	*	*	*	:	*	*	*	*	*	*	*	*	*	*	*	*	*	*	*	*	*	:	:	*	*	*	*	*	:	*	*		*	*	Conservation
0.25							0				0															0	0	0	0					0		0	0		0	0	0	0	0	1	0	0	0				Rel. freq. cuts
1							0				0															2	0	0	1					0		0	0		1	0	0	0	0	2	1	0	0				
2							0				0															2	0	0	1					0		0	1		1	0	1	1	1	3	2	0	0				
5							0				0															6	0	1	2					1		1	3		2	0	4	1	1	5	2	0	0				
10							0				1															8	0	1	3					2		1	3		3	0	5	1	2	6	2	0	1				
120							1				2															15	1	2	3					2		2	4		6	1	4	2	3	8	4	1	2				

	251	252	253	254	255	256	257	258	259	260	261	262	263	264	265	266	267	268	269	270	271	272	273	274	275	276	277	278	279	280	281	282	283	284	285	286	287	288	289	290	291	292	293	294	295	296	297	298	299	300	Residue no.
	P	D	L	K	N	V	K	S	K	I	G	S	T	E	N	L	K	H	Q	P	G	G	G	K	V	Q	I	I	N	K	K	L	D	L	S	N	V	Q	S	K	C	G	S	K	D	N	I	K	H	V	Sequence
																						G	G	K	V	Q	I	I	N	K	K	L	D	L	S	N	V	Q	S	K	C	G	S	K	D	N	I	K	H	V	B-factors
																									b	b	b	b	b	b	b	b	b	b	b	b	b	b	b	b	b										Fib. β sheet
Min	*	*	*	*	*	*	:	*	*	*	*	*	*	*	*	*	*	*	*	*	*	*	*	*	*	*	*	*	*	*	*	*	*	*	*	*	*	*	*	*	*	*	*	*	*	*	*	*	*	*	Conservation
0.25						2							0			1									0	0	0	0							0		0		0		0						0				Rel. freq. cuts
1						7							0			5									0	0	2	2							0		2		0		1						1				
2						13							0			5									0	0	2	2							0		2		0		1						0				
5						23							0			11									1	1	4	6							1		7		0		1						3				
10						30							0			14									1	1	6	8							2		9		2		3						4				
120						40							4			15									2	2	7	11							2		9		4		2						2				

	301	302	303	304	305	306	307	308	309	310	311	312	313	314	315	316	317	318	319	320	321	322	323	324	325	326	327	328	329	330	331	332	333	334	335	336	337	338	339	340	341	342	343	344	345	346	347	348	349	350	Residue no.
	P	G	G	G	S	V	Q	I	V	Y	K	P	V	D	L	S	K	V	T	S	K	C	G	S	L	G	N	I	H	H	K	P	G	G	G	Q	V	E	V	K	S	E	K	L	D	F	K	D	R	V	Sequence
	P	G	G	G	S	V	Q	I	V	Y	K	P	V	D	L	S	K	V	T	S	K	C	G	S	L	G	N	I	H	H																					B-factors
					b	b	b	b	b	b			b	b			b	b									b	b																							Fib. β sheet
Min	*	*	*	*	*	*	:	*	*	*	*	*	*	*	*	*	*	*	*	*	*	*	*	*	*	*	*	*	*	*	*	*	*	*	*	*	*	*	*	*	*	*	*	*	*	*	*	*	*	*	Conservation
0.25								1	0						0	0	0	1	0	0	0	0			0			0											0			0		0					0	0	Rel. freq. cuts
1								7	1						0	0	1	2	1	0	1	1			0			0											0			0		1					0	1	
2								7	1						0	0	1	3	1	0	1	2			0			0											0			0		1					0	1	
5								15	2						1	1	2	4	1	1	2	4			0			1											1			1		11					0	3	
10								17	2						1	1	2	4	2	2	2	5			1			1											2			2		18					1	4	
120								17	2						1	1	2	4	2	2	2	6			1			1											3			1		18					0	5	

	351	352	353	354	355	356	357	358	359	360	361	362	363	364	365	366	367	368	369	370	371	372	373	374	375	376	377	378	379	380	381	382	383	384	385	386	387	388	389	390	391	392	393	394	395	396	397	398	399	400	Residue no.
	Q	S	K	I	G	S	L	D	N	I	T	H	V	P	G	G	G	N	K	K	I	E	T	H	K	L	T	F	R	E	N	A	K	A	K	T	D	H	G	A	E	I	V	Y	K	S	P	V	V	S	Sequence
Min	*	*	*	*	*	*	*	*	*	*	*	*	*	*	*	*	*	:	*	*	*	*	*	*	*	*	*	*	*	*	*	*	*	*	*	*	*	*	*	*	*	*	*	*	*	*	*	*	*	*	Conservation
0.25	0	0	0	0	0	0	0	0	0	1	0	0	0	0	0	0	0	0						0	0	5	0					0		1			0	1		0	0	10	1	0	0	0		0	0	0	Rel. freq. cuts
1	0	0	0	0	0	0	1	0	0	4	0	0	1	0	1	1	0	0						0	0	28	1					0		1			1	3		0	1	34	4	1	1	0		4	2	3	
2	1	0	0	0	0	0	2	0	0	7	0	0	1	0	2	1	0	1						0	0	22	1					0		1			1	3		0	1	38	5	1	1	0		6	1	4	
5	2	0	1	1	1	0	4	0	1	11	2	1	1	1	3	0	0	3						1	1	50	2					0		1			1	3		1	2	50	7	2	1	1		11	5	5	
10	3	1	1	1	1	1	6	0	1	14	2	1	1	1	3	2	1	1						0	2	62	4					1		2			1	2		0	2	51	8	3	2	2		12	7	7	
120	4	1	0	1	1	2	6	1	1	14	2	2	3	1	3	2	0	2						1	1	59	3					0		1			1	2		1	1	49	7	3	2	1		12	6	8	

	401	402	403	404	405	406	407	408	409	410	411	412	413	414	415	416	417	418	419	420	421	422	423	424	425	426	427	428	429	430	431	432	433	434	435	436	437	438	439	440	441	Residue no.
	G	D	T	S	P	R	H	L	S	N	V	S	S	T	G	S	I	D	M	V	D	S	P	Q	L	A	T	L	A	D	E	V	S	A	S	L	A	K	Q	G	L	Sequence
Min	*	*	*	*	*	*	*	*	*	*	*	*	*	*	*	*	*	*	*	*	*	*	*	*	*	*	*	*	*	*	*	*	*	*	*	*	*	*	*	*	*	Conservation
0.25	1	1	1	1	1	0	1	11	0	1	5	0	0	0	0	0		0	1	0	1	0	1	1	2	2	0	3	0	0	0	2	1	1	0			0		1		Rel. freq. cuts
1	2	2	2	3	3	1	4	24	1	3	13	1	1	3	1	1		1	2	2	2	1	2	4	4	5	2	10	0	1	0	4	1	2	1			1		1		
2	2	2	2	2	2	1	3	30	1	2	18	1	1	5	1	0		3	4	4	3	1	3	4	5	7	3	13	2	2	1	8	1	3	1			1		1		
5	3	3	3	4	4	2	4	37	2	5	24	2	1	9	1	2		5	4	7	3	2	4	6	7	9	4	15	2	2	1	13	2	4	3			1		1		
10	4	4	3	5	3	2	5	40	3	6	26	3	0	9	1	2		5	5	7	3	3	4	7	7	11	4	17	1	2	2	15	2	5	3			1		1		
120	4	3	4	3	3	2	5	40	3	4	25	2	1	9	2	0		3	5	8	3	3	4	5	6	11	3	15	1	2	1	14	2	5	3			1		1		

Fig. S2C Proteolysis of fibrillar tau CN by HTRA1.

wt tau	1	2	3	4	5	6	7	8	9	10	11	12	13	14	15	16	17	18	19	20	21	22	23	24	25	26	27	28	29	30	31	32	33	34	35	36	37	38	39	40	41	42	43	44	45	46	47	48	49	50	Residue no.
	M	A	E	P	R	Q	E	F	E	V	M	E	D	H	A	G	T	Y	G	L	G	D	R	K	D	Q	G	G	Y	T	M	H	Q	D	Q	E	G	D	T	D	A	E	I	V	Y	K	S	P	V	V	Sequence
Min	1	2	3	4	5	6	7	8	9	10	11	12	13	14	15	16	17	18	19	20	21	22	23	24	25	26	27	28	29	30	31	32	33	34	35	36	37	38	39	40	41	391	392	393	394	395	396	397	398	399	Residue no. wt tau
0	0									0	0																																0	0		0			0	0	Rel. freq. cuts
0.25	1									1	0																																4	0		0			0	0	
1	1									1	0																																8	1		0			1	1	
10	1									1	0																																6	0		0			3	1	
2	0									0	0																																11	2		0			2	1	
5	0									0	0																																14	3		1			4	3	
120	1									0	1																																15	3		1			6	3	

wt tau	51	52	53	54	55	56	57	58	59	60	61	62	63	64	65	66	67	68	69	70	71	72	73	74	75	76	77	78	79	80	81	82	83	84	85	86	87	88	89	90	91	92	93	94	95	96	97	98	99	100	Residue no.
	S	G	D	T	S	P	R	H	L	S	N	V	S	S	T	G	S	I	D	M	V	D	S	P	Q	L	A	T	L	A	D	E	V	S	A	S	L	A	K	Q	G	L	E	G	T	T	A	E	E	A	Sequence
Min	400	401	402	403	404	405	406	407	408	409	410	411	412	413	414	415	416	417	418	419	420	421	422	423	424	425	426	427	428	429	430	431	432	433	434	435	436	437	438	439	440	441	99	100	101	102	103	104	105	106	Residue no. wt tau
0	0			0			0	0	0	0	0	0	0		0	0					0				0	0	0	0	0	0	0		3		0	0		0		0		1			0					1	Rel. freq. cuts
0.25	0			0			0	1	4	1	0	3	0		0	0					0				0	0	0	0	3	0	0		6		0	0		0		0		3			0					1	
1	0			0			1	2	8	1	1	3	1		0	0					1				0	1	1	1	5	0	0		2		0	0		0		1		8			0					0	
10	1			1			1	1	20	0	0	9	0		3	0					3				1	2	7	2	10	3	1		9		1	2		1		3		13			1					1	
2	0			1			1	2	15	1	1	8	1		1	0					2				1	2	3	1	7	1	0		7		0	0		1		1		10			1					0	
5	1			0			1	2	23	1	1	13	1		2	1					4				1	2	7	2	9	3	1		8		1	2		1		2		14			1					1	
120	1			1			4	2	25	1	1	14	1		4	2					4				0	1	8	2	14	2	0		13		0	2		1		1		7			2					4	

	101	102	103	104	105	106	107	108	109	110	111	112	113	114	115	116	117	118	119	120	121	122	123	124	125	126	127	128	129	130	131	132	133	134	135	136	137	138	139	140	141	142	143	144	145	146	147	148	149	150	Residue no.
	G	I	G	D	T	P	S	L	E	D	E	A	A	G	H	V	T	Q	A	R	M	V	S	K	S	K	D	G	T	G	S	D	D	K	K	A	K	G	A	D	G	K	T	K	I	A	T	P	R	G	Sequence
Min	107	108	109	110	111	112	113	114	115	116	117	118	119	120	121	122	123	124	125	126	127	128	129	130	131	132	133	134	135	136	137	138	139	140	141	142	143	144	145	146	147	148	149	150	151	152	153	154	155	156	Residue no. wt tau
0				0															1			0																													Rel. freq. cuts
0.25				0															2			0																													
1				0															1			0																													
10				0															0			0																													
2				0															1			0																													
5				0															0			0																													
120				1															2			1																													

	151	152	153	154	155	156	157	158	159	160	161	162	163	164	165	166	167	168	169	170	171	172	173	174	175	176	177	178	179	180	181	182	183	184	185	186	187	188	189	190	191	192	193	194	195	196	197	198	199	200	Residue no.
	A	A	P	G	Q	K	G	Q	A	N	A	T	R	I	P	A	K	T	P	P	A	P	K	T	P	P	S	S	G	E	P	P	K	S	G	D	R	S	G	Y	S	S	P	G	S	P	G	T	P	Sequence
Min	157	158	159	160	161	162	163	164	165	166	167	168	169	170	171	172	173	174	175	176	177	178	179	180	181	182	183	184	185	186	187	188	189	190	191	192	193	194	195	196	197	198	199	200	201	202	203	204	205	206	Residue no. wt tau
0																																																			Rel. freq. cuts
0.25																																																			
1																																																			
2																																																			
5																																																			
10																																																			
120																																																			

	201	202	203	204	205	206	207	208	209	210	211	212	213	214	215	216	217	218	219	220	221	222	223	224	225	226	227	228	229	230	231	232	233	234	235	236	237	238	239	240	241	242	243	244	245	246	247	248	249	250	Residue no.
	G	S	R	S	R	T	P	S	L	P	T	P	P	T	R	E	P	K	K	V	A	V	V	R	T	P	P	K	S	P	S	S	A	K	S	R	L	Q	T	A	P	V	P	M	P	D	L	K	N	V	Sequence
Min	207	208	209	210	211	212	213	214	215	216	217	218	219	220	221	222	223	224	225	226	227	228	229	230	231	232	233	234	235	236	237	238	239	240	241	242	243	244	245	246	247	248	249	250	251	252	253	254	255	256	Residue no. wt tau
0																				0	0	0	0	0		0		0		0	0		0	0	0	1	0	0	0	0	0	0					0			0	Rel. freq. cuts
0.25																				1	0	0	0	0		0		0		0	1		0	0	0	2	0	0	0	0	0	0					0			2	
1																				2	1	0	1	0		0		0		0	1		0	1	0	3	0	1	0	0	0	0					0			9	
2																				1	0	0	0	0		0		0		0	1		0	1	1	2	0	2	2	1	1	1					0			13	
5																				3	1	0	0	0		0		0		0	2		0	1	1	4	0	2	1	0	0	0					0			10	
10																				6	1	1	2	0		0		0		0	3		0	1	2	4	0	4	2	1	0	0					0			20	
120																				13	2	2	6	1		1		1		1	6		2	2	4	4	2	8	4	1	1	1					1			33	

	251	252	253	254	255	256	257	258	259	260	261	262	263	264	265	266	267	268	269	270	271	272	273	274	275	276	277	278	279	280	281	282	283	284	285	286	287	288	289	290	291	292	293	294	295	296	297	298	299	300	Residue no.
	K	S	K	I	G	S	T	E	N	L	K	H	Q	P	G	G	G	K	V	Q	I	I	N	K	K	L	D	L	S	N	V	Q	S	K	C	G	S	K	D	N	I	K	H	V	P	G	G	G	S	V	Sequence
	257	258	259	260	261	262	263	264	265	266	267	268	269	270	271	272	273	274	275	276	277	278	279	280	281	282	283	284	285	286	287	288	289	290	291	292	293	294	295	296	297	298	299	300	301	302	303	304	305	306	Residue no. wt tau
Min																G	G	K	V	Q	I	I	N	K	K	L	D	L	S	N	V	Q	S	K	C	G	S	K	D	N	I	K	H	V	P	G	G	G	S	V	B-factors
0	0				1		0			0	0								0	0	0	0	0	0				0			0		0		0	0					0										Rel. freq. cuts
0.25	0				2		0			0	0								0	1	0	0	0	0				0			1		0		1	0					0										
1	1				2		0			4	0								0	1	2	1	0	0				0			3		0		1	1					0										
2	1				0		0			3	0								0	1	2	0	0	1				0			3		0		0	0					1										
5	1				2		1			5	0								0	1	3	1	0	0				0			4		0		1	2					1										
10	1				2		0			8	0								0	3	4	1	1	1				0			8		1		1	2					2										
120	2				0		1			12	1								1	3	6	2	1	3				1			10		2		1	2					2										

	301	302	303	304	305	306	307	308	309	310	311	312	313	314	315	316	317	318	319	320	321	322	323	324	325	326	327	328	329	330	331	332	333	334	335	336	337	338	339	340	341	342	343	344	345	346	347	348	349	350	Residue no.
	Q	I	V	Y	K	P	V	D	L	S	K	V	T	S	K	C	G	S	L	G	N	I	H	H	K	P	G	G	G	Q	V	E	V	K	S	E	K	L	D	F	K	D	R	V	Q	S	K	I	G	S	Sequence
	307	308	309	310	311	312	313	314	315	316	317	318	319	320	321	322	323	324	325	326	327	328	329	330	331	332	333	334	335	336	337	338	339	340	341	342	343	344	345	346	347	348	349	350	351	352	353	354	355	356	Residue no. wt tau
Min	Q	I	V	Y	K	P	V	D	L	S	K	V	T	S	K	C	G	S	L	G	N	I	H	H																											B-factors
0	0	0	0	0					0	0		0	0	0	0	0	0	0	0											0		0	0				0	0					0	0	0	0	0		0		Rel. freq. cuts
0.25	1	3	0	0					0	0		2	0	1	0	0	0	0	0											0		0	0				0	1					0	0	0	0	0		0		
1	1	7	0	0					1	0		3	1	1	1	2	0	0	0											1		0	1				0	2					0	1	0	0	0		0		
2	1	5	0	0					1	1		2	0	1	0	0	0	0	0											0		0	0				0	1					0	2	1	0	0		0		
5	2	10	1	0					1	0		4	1	1	2	3	0	0	0											0		0	2				0	5					0	1	0	1	0		0		
10	3	14	2	0					1	1		5	1	2	2	6	1	0	0											1		1	2				0	9					0	3	1	1	1		1		
120	2	15	3	1					2	0		7	1	1	2	6	0	1	1											1		0	2				1	14					1	4	3	1	3		0		

	351	352	353	354	355	356	357	358	359	360	361	362	363	364	365	366	367	368	369	370	371	372	373	374	375	376	377	378	379	380	381	382	383	384	385	386	387	388	389	390	391	392	393	394	395	396	397	398	399	400	Residue no.
	L	D	N	I	T	H	V	P	G	G	G	N	K	K	I	E	T	H	K	L	T	F	R	E	N	A	K	A	K	T	D	H	G	A	G	L	K	E	S	P	L	Q	T	P	T	E	D	G	S	E	Sequence
Min	357	358	359	360	361	362	363	364	365	366	367	368	369	370	371	372	373	374	375	376	377	378	379	380	381	382	383	384	385	386	387	388	389	390	42	43	44	45	46	47	48	49	50	51	52	53	54	55	56	57	Residue no. wt tau
0	0	0		0	0	0	0	0	0	0						0	0	0	0	0	0										0			0			0														Rel. freq. cuts
0.25	0	0		2	0	0	0	0	0	0						0	0	1	0	1	0										1			0			0														
1	2	0		8	0	1	1	1	1	1						2	0	2	1	8	1										1			1			0														
2	0	0		4	0	0	1	1	1	0						0	0	0	0	3	0										0			0			0														
5	2	0		12	0	2	2	2	2	1						1	1	2	2	13	2										1			1			0														
10	4	0		15	1	2	3	3	3	3						5	1	3	3	20	3										1			1			1														
120	5	1		17	2	2	5	3	3	3						5	1	3	2	20	2										1			1			1														

	401	402	403	404	405	406	407	408	409	410	411	412	413	414	415	416	417	418	419	420	421	422	423	424	425	426	427	428	429	430	431	432	433	434	435	436	437	438	439	440	441										Residue no.
	E	P	G	S	E	T	S	D	A	K	S	T	P	T	A	E	D	V	T	A	P	L	V	D	E	G	A	P	G	K	Q	A	A	A	Q	P	H	T	E	I	P										Sequence
Min	58	59	60	61	62	63	64	65	66	67	68	69	70	71	72	73	74	75	76	77	78	79	80	81	82	83	84	85	86	87	88	89	90	91	92	93	94	95	96	97	98										Residue no. wt tau
0					0																										0																				Rel. freq. cuts
0.25					0																										0																				
1					0																										0																				
2					0																										0																				
5					0																										0																				
10					1																										0																				
120					0																										1																				

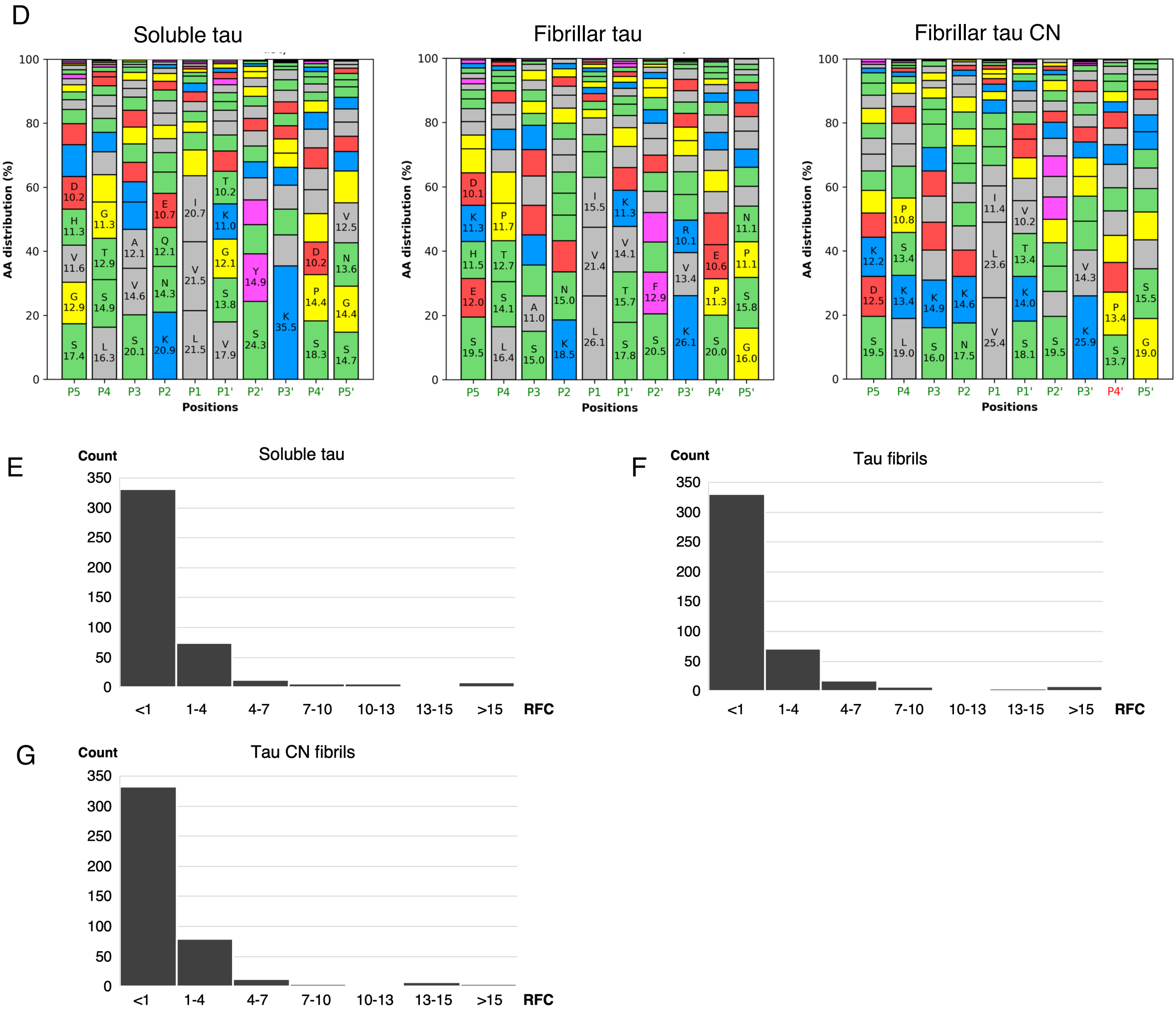

Fig. S2. Proteolysis of soluble, fibrillar and fibrillar tau CN by HTRA1.
Proteolysis of soluble, fibrillar and fibrillar tau CN by HTRA1 was done as described in Fig. 2A. Samples were taken at the timepoints indicated (Min) and proteolytic products of tau were identified by LC-MS. Structural elements i.e. helices and loops (2ndary struct.), B-factors as detected in the cryo EM structure of fibrillar tau (pdb:6QJH) are indicated. Amino acid sequence conservation (Conservation) is derived from a multiple sequence alignment (Supplemental data 2); * = identical, : = conserved residues. P1 residues identified in n=4 experiments as significantly enriched compared to controls are represented by numbers indicating the relative frequency of cuts (Rel. freq. cuts) at each time point. No number indicates that no cleavage was detected at any of the time points investigated.
(A) Proteolysis of soluble tau by HTRA1.

(B) Proteolysis of fibrillar tau by HTRA1.

(C) Proteolysis of fibrillar tau CN by HTRA1. In fibrillar tau CN, parts of the N- and C-termini have been switched i.e. the C-terminal residues 391-441 were swapped with the N-terminal residues 42-98 (highlighted in grey).

(D) Relative amino acid distribution at P1-P5 and P1'-P5' sites of tau variants used in this study. X-axis: red letter, no protease selectivity toward specific amino acids at this position; green letter, protease selectivity toward certain amino acids at this position. Amino acid residue color code: Hydrophobic, grey; hydrophilic, green; positively charged, red; negatively charged, blue; Gly and Pro, yellow.

(E-G) Histograms of relative cleavage efficiencies. To classify cleavage sites, the relative cleavage efficiencies after each residue was calculated by UMSAP using data of the 120 min timepoints. The histograms were generated by Xcel using width of interval: 3, underflow bin: 0.99; overflow bin: 20 as parameters. RFC, relative frequency of cuts.

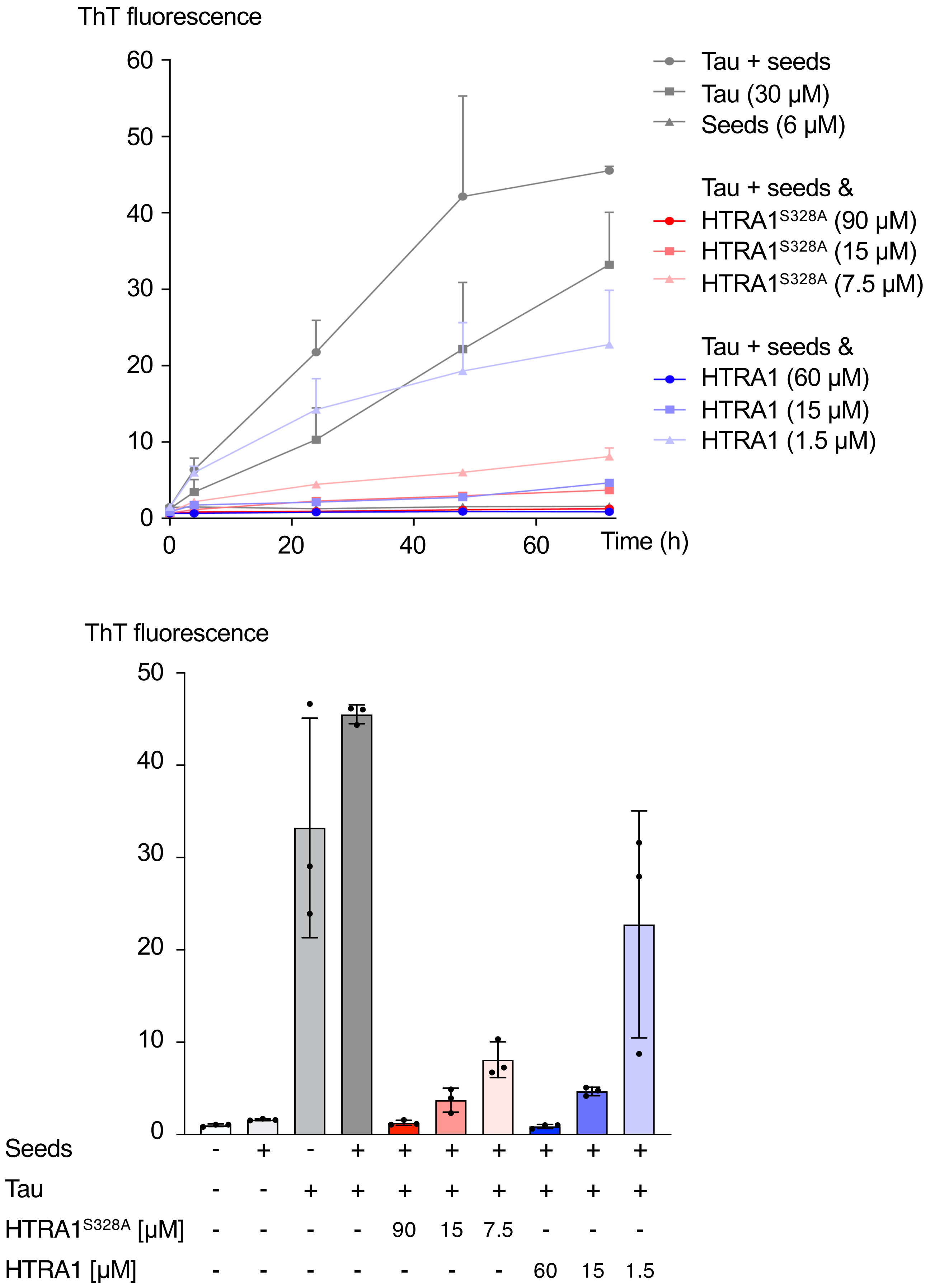

Fig. S3. Quantification of tau seeding based on ThT fluorescence. Top, in vitro seeding of tau aggregation was performed as described in Fig. 1. ThT fluorescence was measured at 480 nm at the time points indicated. Measured ThT signals were normalized to the corresponding buffer controls. Bottom, for better comparison, endpoint values (72 h) were plotted as bar diagrams. Individual data points are shown, Error bars indicate standard deviation (SD); n = 3.

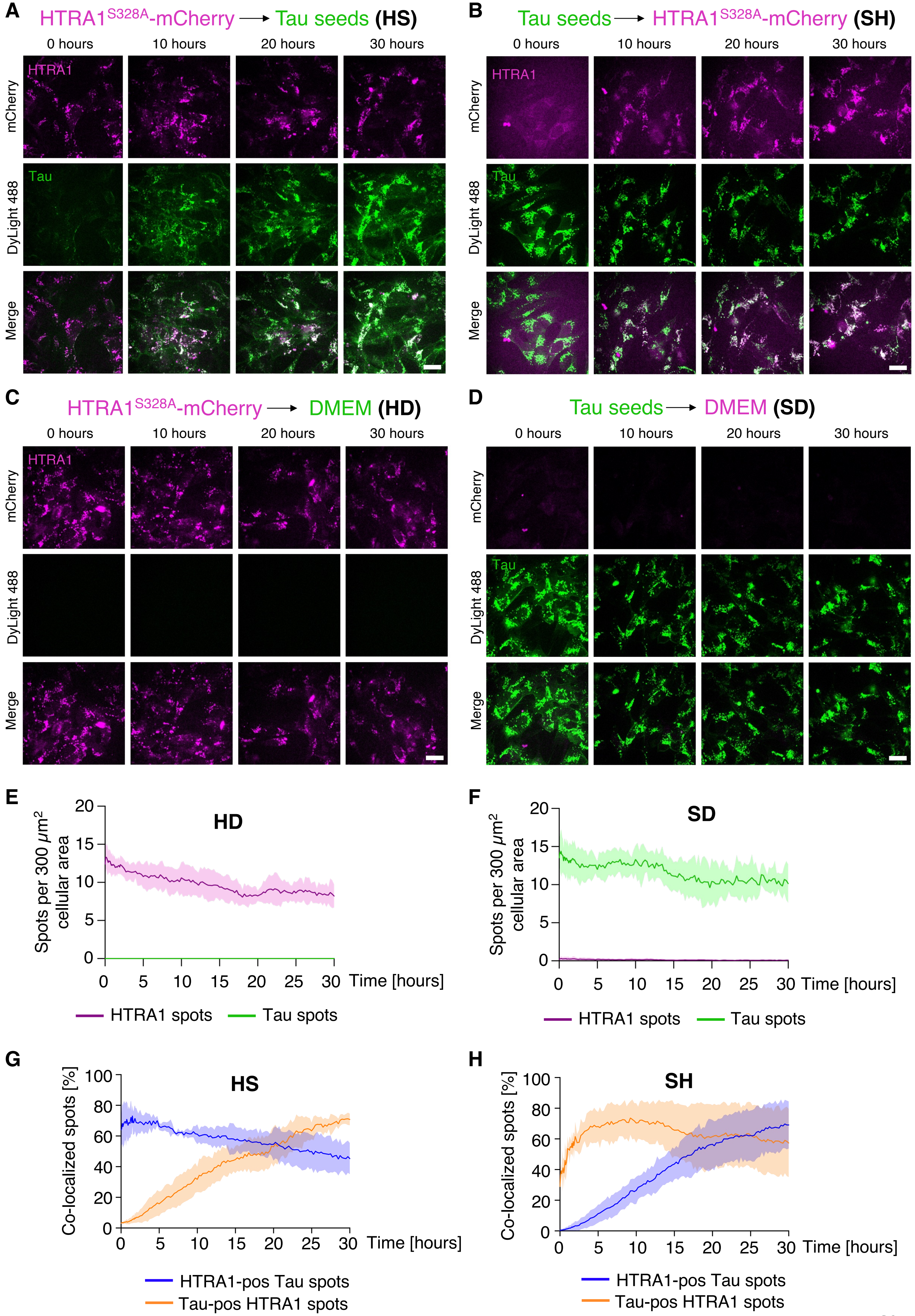
A
HTRA1S328A-mCherry → Tau seeds (HS)
B
Tau seeds → HTRA1S328A-mCherry (SH)
0 hours
10 hours
20 hours
30 hours
mCherry
DyLight 488
Merge
HTRA1
Tau
C
HTRA1S328A-mCherry → DMEM (HD)
D
Tau seeds → DMEM (SD)
E
HD
Spots per 300 μm2 cellular area
Time [hours]
HTRA1 spots
Tau spots
F
SD
G
HS
Co-localized spots [%]
HTRA1-pos Tau spots
Tau-pos HTRA1 spots
H
SH

I

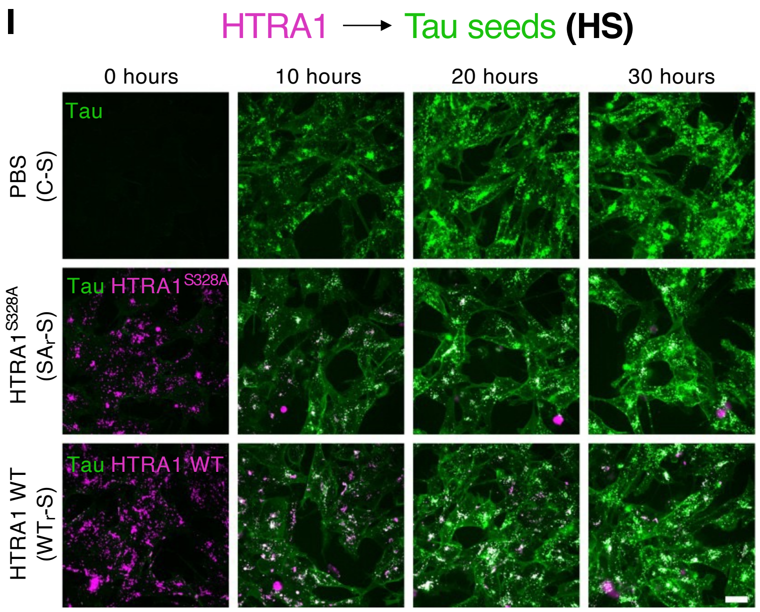

HTRA1 → Tau seeds (HS)
0 hours
10 hours
20 hours
30 hours
PBS (C-S)
HTRA1^{S328A} (SA$_r$-S)
HTRA1 WT (WT$_r$-S)
Tau
Tau HTRA1^{S328A}
Tau HTRA1 WT

J

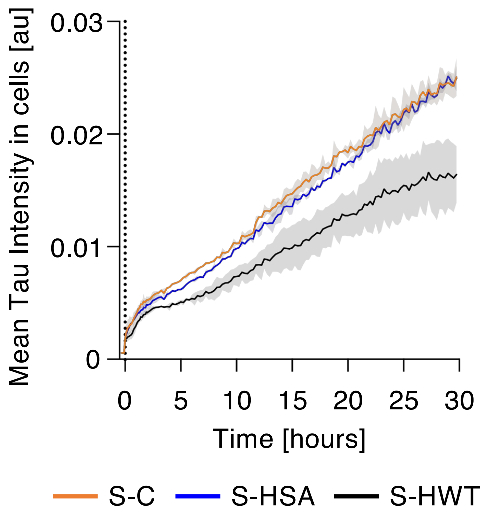

Mean Tau Intensity in cells [au]
0.03
0.02
0.01
0
0
5
10
15
20
25
30
Time [hours]
S-C
S-HSA
S-HWT

K

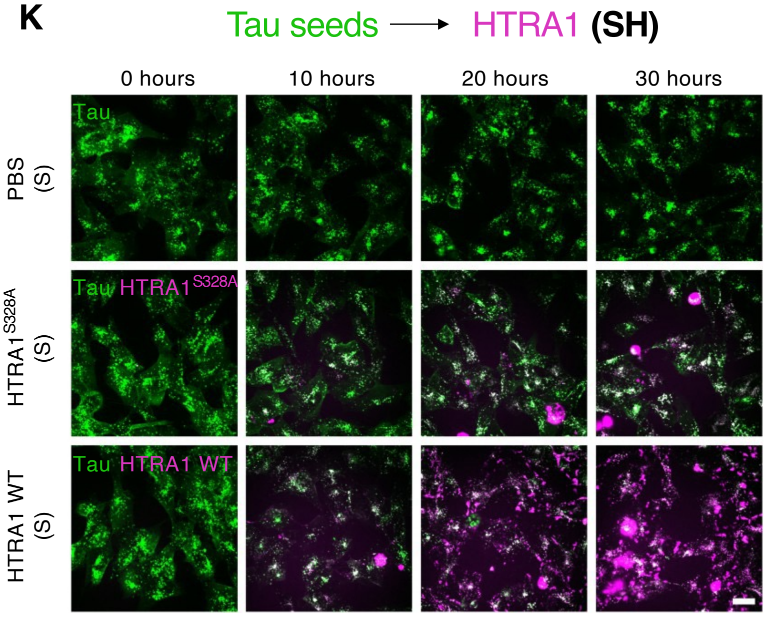

Tau seeds → HTRA1 (SH)
0 hours
10 hours
20 hours
30 hours
PBS (S)
HTRA1^{S328A} (S)
HTRA1 WT (S)
Tau
Tau HTRA1^{S328A}
Tau HTRA1 WT

L

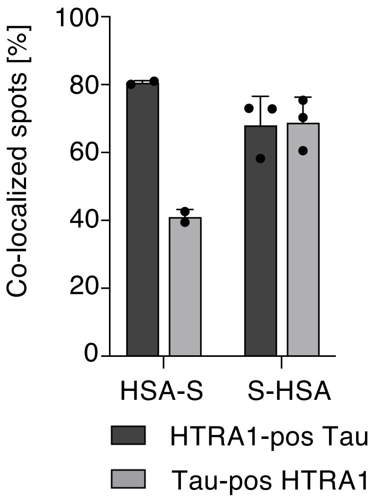

Co-localized spots [%]
100
80
60
40
20
0
HSA-S
S-HSA
HTRA1-pos Tau
Tau-pos HTRA1

Fig. S4. Live cell analysis of SH-SY5Y cells treated with secreted HTRA1^{S328A}-mCherry and/or fluorescently labeled tau seeds. (A-D) Live cell imaging of SH-SY5Y cells. Scale bars: 20 µm.
(A) HS treatment: Cells were treated with conditioned medium containing secreted HTRA1S328A-mCherry (magenta) 24 h before imaging and DyLight 488-labeled tau seeds (green) immediately prior to imaging.

(B) SH treatment: Cells were treated with DyLight 488-labeled tau seeds (green) 24 h before imaging and conditioned medium containing secreted HTRA1S328A-mCherry (magenta) immediately prior to imaging.

(C) HD treatment: Cells were treated with conditioned medium containing secreted HTRA1S328A-mCherry (magenta) 24 h before imaging and DMEM immediately prior to imaging.

(D) SD treatment: Cells were treated with DyLight 488-labeled tau seeds (green) 24 h before imaging and DMEM immediately prior to imaging.

(E–F) Time-resolved analysis of HTRA1S328A-mCherry and DyLight 488-labeled tau seed spots in SH-SY5Y control treatment cells (10 random positions per condition/experiment, n=3; in total, more than 10,500 individual HTRA1 and Tau spots were detected and subsequently analyzed). Cells were either treated first with HTRA1S328A-mCherry followed by DMEM (HD shown in E) or DyLight 488-labeled tau seeds followed by DMEM (SD shown in F). In each frame, the CellTracker Deep Red stain was used to detect the image area that was covered with cells (total cell area). Discrete fluorescent spots were detected in the mCherry and DyLight 488 channel in this region only. The number of detected discrete HTRA1S328A-mCherry (magenta line) and/or tau seed spots (green line) was normalized by the measured cell area (number of spots/µm2) and multiplied by the factor 300, which reflects the average cell area of an individual SH-SY5Y cell. Data show mean ± SD.

(G-H) Time-resolved co-localization analysis of HTRA1S328A-mCherry and DyLight 488-labeled tau seed spots (corresponding data from Fig. 6E). Discrete spots of HTRA1S328A-mCherry were detected and masked with DyLight 488-labeled tau seed spots to identify double-positive species (Tau-positive HTRA1 spots) or vice versa (HTRA1-positive Tau spots). Data show mean ± SD.

(I-L) Live cell analysis of SH-SY5Y cells treated with recombinant DyLight 633 labeled HTRA1S328A or HTRA1 WT and/or fluorescently labeled tau seeds.

(I) Cells were treated with PBS as a control (C-S), DyLight 633 labeled HTRA1S328A (HSA-S) or DyLight 633 HTRA1 WT (HWT-S) 24 h before imaging (magenta). DyLight 488 labeled tau seeds (green) were added at the microscope and imaging continued for 30 h. Scale bars: 20 µm.

(J) Time-resolved analysis of tau signal intensity in cells. Cell areas covered with SH-SY5Y cells (total cell area) were detected based on CellTracker Orange stain. Mean intensity of DyLight 488 in this total cell area was measured for each time point (10 random positions per condition/experiment, n=2; in total, >50,000 individual HTRA1 and Tau spots were analyzed). Dashed line shows time-point of PBS (S-C), DyLight 633 HTRA1S328A (S-HSA) or DyLight 633 HTRA1 WT (S-HWT) on-stage addition.

(K) Cells were treated with DyLight 488 labeled tau seeds (green) 24 h before imaging. PBS (S-C) or DyLight 633 labeled HTRA1S328A (S-HSA) or WT (S-HWT) (magenta) were added at the microscope and imaging continued for 30 hours. Scale bars: 20 µm.

(L) Co-localization analysis of recombinant DyLight 633 labeled HTRA1S328A and DyLight 488-labeled tau seed spots at time point 20 h after start of imaging. Discrete spots of HTRA1S328A were detected and masked with DyLight 488 labeled tau seed spots to identify double-positive species (Tau-positive HTRA1 spots) or vice versa (HTRA1-positive Tau spots). Data are presented as mean ± SD (n = 3); dots represent mean values from individual experiments. Cells were either treated first with HTRA1S328A followed by tau seeds (HS) or vice versa (SH). The lower colocalization of tau positive HTRA1 signals in HSA-S is due to a slower uptake of HTRA1.

Supporting data 1. List of all cross-linked peptides identified.

See methods for details. The data are provided as an extra Xcel file. The data were filtered using a false discovery rate (FDR) of 1%, Match score and match counts are shown.
LinkedRes1 and LinkedRes2 indicate the position of the cross-linked residues identified.

Supporting data 2. Peptides identified in soluble tau digested by HTRA1

2N4R tau

MAEPRQEFEVMEDHAGTYGLGDRKDQGGYTMHQDQEGDTDAGLKESPLQTPTEDGSEEPG
SETSDAKSTPTAEDVTAPLVDEGAPGKQAAAQPHTEIP EGTTAEEAGIGDTPSLEDEAAG
HVTQARMVSKSKDGTGSDDKKAKGADGKTKIATPRGAAPPGQKGQANATRIPAKTPPAPK
TPPSSGEPPKSGDRSGYSSPGSPGTPGSRSRTPSLPTPPTREPKKVAVVRTPPKSPSSAK
SRLQTAPVPMPDLKNVKSKIGSTENLKHQPGGGKVQIINKKLDLSNVQSKCGSKDNIKHV
PGGGSVQIVYKPVDLSKVTSKCGSLGNIHHKPGGGQVEVKSEKLDFKDRVQSKIGSLDNI
THVPGGGNKKIETHKLTFRENAKAKTDHGA EIVYKSPVVSGDTSPRHLSNVSSTGSIDMV
DSPQLATLADEVSASLAKQGL

Soluble Tau 15 sec

```
  1 MAEPRQEFEVMEDHAGTYGLGDRKDQGGYTMHQDQEGDTDAGLKESPLQTPTEDGSEEPG

  1 SETSDAKSTPTAEDVTAPLVDEGAPGKQAAAQPHTEIPEGTTAEEAGIGDTPSLEDEAAG

  1 HVTQARMVSKSKDGTGSDDKKAKGADGKTKIATPRGAAPPGQKGQANATRIPAKTPPAPK

  1 TPPSSGEPPKSGDRSGYSSPGSPGTPGSRSRTPSLPTPPTREPKKVAVVRTPPKSPSSAK
  2                                               AVVRTPPKSPSSAK

  1 SRLQTAPVPMPDLKNVKSKIGSTENLKHQPGGGKVQIINKKLDLSNVQSKCGSKDNIKHV
  2 SRLQTAPVPMPDLKNV
  3     TAPVPMPDLKNV
  4                 KSKIGSTENL
  5                                      INKKLDLSNVQSKC
  6                                       NKKLDLSNVQSKC
  7                                         KLDLSNVQSKC
  8                                           DLSNVQSKCGSKDNIKHV
  9                                                    GSKDNIKHV

  1 PGGGSVQIVYKPVDLSKVTSKCGSLGNIHHKPGGGQVEVKSEKLDFKDRVQSKIGSLDNI
  8 PGGGSVQI
  9 PGGGSVQI
 10         VYKPVDLSKV
 11         VYKPVDLSKVTSKC
 12           KPVDLSKVTSKC
 13                       GSLGNIHHKPGGGQVEVKSEKL
 14                                             DFKDRVQSKIGSLDNI

  1 THVPGGGNKKIETHKLTFRENAKAKTDHGAEIVYKSPVVSGDTSPRHLSNVSSTGSIDMV
 15 THVPGGGNKKIETHKL
 16                 TFRENAKAKTDHGAEI
 17                 TFRENAKAKTDHGAEIV
 18                 TFRENAKAKTDHGAEIVY
 19                 TFRENAKAKTDHGAEIVYK
 20                                 VYKSPVVSG
 21                                 VYKSPVVSGDTSPRHL
 22                                 VYKSPVVSGDTSPRHLS
 23                                 VYKSPVVSGDTSPRHLSN
 24                                 VYKSPVVSGDTSPRHLSNV
 25                                 VYKSPVVSGDTSPRHLSNVS
 26                                 VYKSPVVSGDTSPRHLSNVSSTG
 27                                 VYKSPVVSGDTSPRHLSNVSSTGSIDM
 28                                 VYKSPVVSGDTSPRHLSNVSSTGSIDMV
 29                                 VYKSPVVSGDTSPRHLSNVSSTGSIDMV
 30                                 VYKSPVVSGDTSPRHLSNVSSTGSIDMV
 31                                                 SNVSSTGSIDMV
 32                                                 SNVSSTGSIDMV
 33                                                 SNVSSTGSIDMV
 34                                                 SNVSSTGSIDMV
 35                                                 SNVSSTGSIDMV
 36                                                 SNVSSTGSIDMV

  1 DSPQLATLADEVSASLAKQGL
 28 D
 29 DSPQL
 30 DSPQLATL
 31 DSPQL
 32 DSPQLA
 33 DSPQLATL
 34 DSPQLATLADEV
 35 DSPQLATLADEVSA
```

36 DSPQLATLADEVSASLAKQGL
37 ATLADEVSASLAKQGL
38 TLADEVSASLAKQGL
39 LADEVSASLAKQGL
40 ADEVSASLAKQGL
41 VSASLAKQGL
42 SASLAKQGL

Soluble Tau 1 min

```
  1 MAEPRQEFEVMEDHAGTYGLGDRKDQGGYTMHQDQEGDTDAGLKESPLQTPTEDGSEEPG

  1 SETSDAKSTPTAEDVTAPLVDEGAPGKQAAAQPHTEIPEGTTAEEAGIGDTPSLEDEAAG

  1 HVTQARMVSKSKDGTGSDDKKAKGADGKTKIATPRGAAPPGQKGQANATRIPAKTPPAPK

  1 TPPSSGEPPKSGDRSGYSSPGSPGTPGSRSRTPSLPTPPTREPKKVAVVRTPPKSPSSAK
  2                                               AVVRTPPKSPSSAK

  1 SRLQTAPVPMPDLKNVKSKIGSTENLKHQPGGGKVQIINKKLDLSNVQSKCGSKDNIKHV
  2 SRLQTAPVPMPDLKNV
  3     TAPVPMPDLKNV
  4                 KSKIGSTENL
  5                                      INKKLDLSNV
  6                                      INKKLDLSNVQSKC
  7                                       NKKLDLSNV
  8                                         KLDLSNVQSKC
  9                                           DLSNVQSKC
 10                                           DLSNVQSKCGSKDNIKHV
 11                                                QSKCGSKDNIKHV
 12                                                    GSKDNIKHV

  1 PGGGSVQIVYKPVDLSKVTSKCGSLGNIHHKPGGGQVEVKSEKLDFKDRVQSKIGSLDNI
 10 PGGGSVQI
 11 PGGGSVQI
 12 PGGGSVQI
 13   GGSVQIVYKPVDLSKVTSKC
 14         VYKPVDLS
 15         VYKPVDLSKV
 16         VYKPVDLSKVT
 17         VYKPVDLSKVTSKC
 18          YKPVDLSKVTSKC
 19              DLSKVTSKC
 20              DLSKVTSKCGSLGNI
 21                   TSKCGSLGNI
 22                       GSLGNIHHKPGGGQVEVKSEKL
 23                                             DFKDRVQSKIGSLDNI
 24                                                   QSKIGSLDNI

  1 THVPGGGNKKIETHKLTFRENAKAKTDHGAEIVYKSPVVSGDTSPRHLSNVSSTGSIDMV
 25 THVPGGGNKKIETHKL
 26                 TFRENAKAKTDHGAEI
 27                 TFRENAKAKTDHGAEIV
 28                 TFRENAKAKTDHGAEIVY
 29                 TFRENAKAKTDHGAEIVYK
 30                                 VYKSPVVSG
 31                                 VYKSPVVSGD
 32                                 VYKSPVVSGDT
 33                                 VYKSPVVSGDTS
 34                                 VYKSPVVSGDTSPR
 35                                 VYKSPVVSGDTSPRH
 36                                 VYKSPVVSGDTSPRHL
 37                                 VYKSPVVSGDTSPRHLS
 38                                 VYKSPVVSGDTSPRHLSN
 39                                 VYKSPVVSGDTSPRHLSNV
 40                                 VYKSPVVSGDTSPRHLSNVS
 41                                 VYKSPVVSGDTSPRHLSNVSST
 42                                 VYKSPVVSGDTSPRHLSNVSSTG
 43                                 VYKSPVVSGDTSPRHLSNVSSTGSID
 44                                 VYKSPVVSGDTSPRHLSNVSSTGSIDM
 45                                 VYKSPVVSGDTSPRHLSNVSSTGSIDMV
```

```
46                                 VYKSPVVSGDTSPRHLSNVSSTGSIDMV
47                                 VYKSPVVSGDTSPRHLSNVSSTGSIDMV
48                                 VYKSPVVSGDTSPRHLSNVSSTGSIDMV
49                                 VYKSPVVSGDTSPRHLSNVSSTGSIDMV
50                                 VYKSPVVSGDTSPRHLSNVSSTGSIDMV
51                                                 SNVSSTGSIDM
52                                                 SNVSSTGSIDMV
53                                                 SNVSSTGSIDMV
54                                                 SNVSSTGSIDMV
55                                                 SNVSSTGSIDMV
56                                                 SNVSSTGSIDMV
57                                                 SNVSSTGSIDMV
58                                                 SNVSSTGSIDMV
59                                                 SNVSSTGSIDMV
60                                                 SNVSSTGSIDMV
61                                                 SNVSSTGSIDMV
62                                                 SNVSSTGSIDMV
63                                                    SSTGSIDMV
64                                                    SSTGSIDMV
65                                                    SSTGSIDMV

 1 DSPQLATLADEVSASLAKQGL
46 D
47 DSP
48 DSPQL
49 DSPQLA
50 DSPQLATL
53 D
54 DS
55 DSP
56 DSPQ
57 DSPQL
58 DSPQLA
59 DSPQLATL
60 DSPQLATLADEV
61 DSPQLATLADEVSA
62 DSPQLATLADEVSASLAKQGL
63 DSPQL
64 DSPQLATL
65 DSPQLATLADEV
66     LATLADEVSASLAKQGL
67      ATLADEVSASLAKQGL
68       TLADEVSASLAKQGL
69        LADEVSASLAKQGL
70         ADEVSASLAKQGL
71             SASLAKQGL
```

Soluble Tau 2 min

```
 1 MAEPRQEFEVMEDHAGTYGLGDRKDQGGYTMHQDQEGDTDAGLKESPLQTPTEDGSEEPG
 2  AEPRQEFEV

 1 SETSDAKSTPTAEDVTAPLVDEGAPGKQAAAQPHTEIPEGTTAEEAGIGDTPSLEDEAAG

 1 HVTQARMVSKSKDGTGSDDKKAKGADGKTKIATPRGAAPPGQKGQANATRIPAKTPPAPK

 1 TPPSSGEPPKSGDRSGYSSPGSPGTPGSRSRTPSLPTPPTREPKKVAVVRTPPKSPSSAK
 3                                               AVVRTPPKSPSSAK
 4                                               AVVRTPPKSPSSAK

 1 SRLQTAPVPMPDLKNVKSKIGSTENLKHQPGGGKVQIINKKLDLSNVQSKCGSKDNIKHV
 3 SRLQ
 4 SRLQTAPVPMPDLKNV
 5     TAPVPMPDLKNV
 6                 KSKIGSTENL
 7                                      INKKLDLSNV
 8                                      INKKLDLSNVQS
 9                                      INKKLDLSNVQSKC
10                                       NKKLDLSNV
11                                       NKKLDLSNVQSKC
12                                         KLDLSNVQS
13                                         KLDLSNVQSKC
14                                           DLSNVQSKC
15                                           DLSNVQSKCGSKDNIKHV
16                                                QSKCGSKDNIKHV
17                                                  KCGSKDNIKHV
18                                                    GSKDNIKHV
19                                                    GSKDNIKHV

 1 PGGGSVQIVYKPVDLSKVTSKCGSLGNIHHKPGGGQVEVKSEKLDFKDRVQSKIGSLDNI
15 PGGGSVQI
16 PGGGSVQI
17 PGGGSVQI
18 PGGGSVQI
19 PGGGSVQIVYKPVDLSKVTSKC
20   GGSVQIVYKPVDLSKVTSKC
21         VYKPVDLS
22         VYKPVDLSKV
23         VYKPVDLSKVT
24         VYKPVDLSKVTSK
25         VYKPVDLSKVTSKC
26         VYKPVDLSKVTSKCGSLGNI
27          YKPVDLSKV
28          YKPVDLSKVTSKC
29              DLSKVTSKC
30              DLSKVTSKCGSLGNI
31                       GSLGNIHHKPGGGQVEV
32                       GSLGNIHHKPGGGQVEVKSEKL
33                          GNIHHKPGGGQVEVKSEKL
34                             HHKPGGGQVEVKSEKL
35                                             DFKDRVQSKIGSLDNI

 1 THVPGGGNKKIETHKLTFRENAKAKTDHGAEIVYKSPVVSGDTSPRHLSNVSSTGSIDMV
36 THVPGGGNKKIETHKL
37      GGNKKIETHKL
38                 TFRENAKAKTD
39                 TFRENAKAKTDHGAEI
40                 TFRENAKAKTDHGAEIV
41                 TFRENAKAKTDHGAEIVY
42                                 VYKSPVVSG
```

```
43                                 VYKSPVVSGD
44                                 VYKSPVVSGDT
45                                 VYKSPVVSGDTS
46                                 VYKSPVVSGDTSP
47                                 VYKSPVVSGDTSPR
48                                 VYKSPVVSGDTSPRH
49                                 VYKSPVVSGDTSPRHL
50                                 VYKSPVVSGDTSPRHLS
51                                 VYKSPVVSGDTSPRHLSN
52                                 VYKSPVVSGDTSPRHLSNV
53                                 VYKSPVVSGDTSPRHLSNVS
54                                 VYKSPVVSGDTSPRHLSNVSS
55                                 VYKSPVVSGDTSPRHLSNVSST
56                                 VYKSPVVSGDTSPRHLSNVSSTG
57                                 VYKSPVVSGDTSPRHLSNVSSTGS
58                                 VYKSPVVSGDTSPRHLSNVSSTGSID
59                                 VYKSPVVSGDTSPRHLSNVSSTGSIDM
60                                 VYKSPVVSGDTSPRHLSNVSSTGSIDMV
61                                 VYKSPVVSGDTSPRHLSNVSSTGSIDMV
62                                 VYKSPVVSGDTSPRHLSNVSSTGSIDMV
63                                 VYKSPVVSGDTSPRHLSNVSSTGSIDMV
64                                 VYKSPVVSGDTSPRHLSNVSSTGSIDMV
65                                 VYKSPVVSGDTSPRHLSNVSSTGSIDMV
66                                  YKSPVVSGDTSPRHL
67                                       VSGDTSPRHL
68                                                 SNVSSTGSIDM
69                                                 SNVSSTGSIDMV
70                                                 SNVSSTGSIDMV
71                                                 SNVSSTGSIDMV
72                                                 SNVSSTGSIDMV
73                                                 SNVSSTGSIDMV
74                                                 SNVSSTGSIDMV
75                                                 SNVSSTGSIDMV
76                                                 SNVSSTGSIDMV
77                                                 SNVSSTGSIDMV
78                                                 SNVSSTGSIDMV
79                                                 SNVSSTGSIDMV
80                                                 SNVSSTGSIDMV
81                                                 SNVSSTGSIDMV
82                                                 SNVSSTGSIDMV
83                                                    SSTGSIDMV
84                                                    SSTGSIDMV
85                                                    SSTGSIDMV
86                                                    SSTGSIDMV
87                                                    SSTGSIDMV
88                                                       GSIDMV
89                                                       GSIDMV

 1 DSPQLATLADEVSASLAKQGL
60 D
61 DSP
62 DSPQ
63 DSPQL
64 DSPQLA
65 DSPQLATL
70 D
71 DS
72 DSP
73 DSPQ
74 DSPQL
75 DSPQLA
76 DSPQLAT
77 DSPQLATL
```

```
78 DSPQLATLAD
79 DSPQLATLADE
80 DSPQLATLADEV
81 DSPQLATLADEVSA
82 DSPQLATLADEVSASLAKQGL
83 DSPQL
84 DSPQLA
85 DSPQLATL
86 DSPQLATLADEV
87 DSPQLATLADEVSASLAKQGL
88 DSPQLA
89 DSPQLATL
90 DSPQLATLADEV
91       TLADEVSASLAKQGL
92        LADEVSASLAKQGL
93         ADEVSASLAKQ
94         ADEVSASLAKQG
95         ADEVSASLAKQGL
96           EVSASLAKQGL
97             SASLAKQGL
```

Soluble Tau 5 min

```
 1 MAEPRQEFEVMEDHAGTYGLGDRKDQGGYTMHQDQEGDTDAGLKESPLQTPTEDGSEEPG
 2  AEPRQEFEVM

 1 SETSDAKSTPTAEDVTAPLVDEGAPGKQAAAQPHTEIPEGTTAEEAGIGDTPSLEDEAAG

 1 HVTQARMVSKSKDGTGSDDKKAKGADGKTKIATPRGAAPPGQKGQANATRIPAKTPPAPK

 1 TPPSSGEPPKSGDRSGYSSPGSPGTPGSRSRTPSLPTPPTREPKKVAVVRTPPKSPSSAK
 3                                               AVVRTPPKSPSSAK

 1 SRLQTAPVPMPDLKNVKSKIGSTENLKHQPGGGKVQIINKKLDLSNVQSKCGSKDNIKHV
 3 SRLQTAPVPMPDLKNV
 4     TAPVPMPDLKNV
 5                 KSKIGSTENL
 6                 KSKIGSTENLKHQPGGGKVQII
 7                           KHQPGGGKVQI
 8                           KHQPGGGKVQII
 9                           KHQPGGGKVQIINKKLDLSNV
10                           KHQPGGGKVQIINKKLDLSNVQSKC
11                                    QIINKKLDLSNV
12                                      INKKLDLSNV
13                                      INKKLDLSNVQSKC
14                                       NKKLDLSNV
15                                       NKKLDLSNVQSKC
16                                         KLDLSNVQSKC
17                                           DLSNVQSKC
18                                           DLSNVQSKCGSKDNIKHV
19                                             SNVQSKCGSKDNIKHV
20                                              NVQSKCGSKDNIKHV
21                                                QSKCGSKDNIKHV
22                                                    GSKDNIKHV

 1 PGGGSVQIVYKPVDLSKVTSKCGSLGNIHHKPGGGQVEVKSEKLDFKDRVQSKIGSLDNI
18 PGGGSVQI
19 PGGGSVQI
20 PGGGSVQI
21 PGGGSVQI
22 PGGGSVQI
23   GGSVQIVYKPVDLSKVTSKC
24         VYKPVDLS
25         VYKPVDLSKV
26         VYKPVDLSKVT
27         VYKPVDLSKVTSK
28         VYKPVDLSKVTSKC
29         VYKPVDLSKVTSKCGSL
30         VYKPVDLSKVTSKCGSLGNIH
31          YKPVDLSKV
32          YKPVDLSKVTSKC
33          YKPVDLSKVTSKCGSLGNI
34              DLSKVTSKC
35              DLSKVTSKCGSLGNI
36                   TSKCGSLGNI
37                       GSLGNIHHKPGGGQVEV
38                       GSLGNIHHKPGGGQVEVKSEKL
39                                             DFKDRVQSKIGSLDNI
40                                                   QSKIGSLDNI

 1 THVPGGGNKKIETHKLTFRENAKAKTDHGAEIVYKSPVVSGDTSPRHLSNVSSTGSIDMV
41 THVPGGGNKKIETHKL
42                 TFRENAKAKTDHGAEI
43                 TFRENAKAKTDHGAEIV
```

```
44                 TFRENAKAKTDHGAEIVY
45                 TFRENAKAKTDHGAEIVYK
46                 TFRENAKAKTDHGAEIVYKSPVVSGDTSPRHL
47                                 VYKSPVVSG
48                                 VYKSPVVSGD
49                                 VYKSPVVSGDT
50                                 VYKSPVVSGDTS
51                                 VYKSPVVSGDTSP
52                                 VYKSPVVSGDTSPR
53                                 VYKSPVVSGDTSPRH
54                                 VYKSPVVSGDTSPRHL
55                                 VYKSPVVSGDTSPRHLS
56                                 VYKSPVVSGDTSPRHLSN
57                                 VYKSPVVSGDTSPRHLSNV
58                                 VYKSPVVSGDTSPRHLSNVS
59                                 VYKSPVVSGDTSPRHLSNVSS
60                                 VYKSPVVSGDTSPRHLSNVSST
61                                 VYKSPVVSGDTSPRHLSNVSSTG
62                                 VYKSPVVSGDTSPRHLSNVSSTGS
63                                 VYKSPVVSGDTSPRHLSNVSSTGSID
64                                 VYKSPVVSGDTSPRHLSNVSSTGSIDM
65                                 VYKSPVVSGDTSPRHLSNVSSTGSIDMV
66                                 VYKSPVVSGDTSPRHLSNVSSTGSIDMV
67                                 VYKSPVVSGDTSPRHLSNVSSTGSIDMV
68                                 VYKSPVVSGDTSPRHLSNVSSTGSIDMV
69                                 VYKSPVVSGDTSPRHLSNVSSTGSIDMV
70                                 VYKSPVVSGDTSPRHLSNVSSTGSIDMV
71                                 VYKSPVVSGDTSPRHLSNVSSTGSIDMV
72                                  YKSPVVSGDTSPRHL
73                                       VSGDTSPRHL
74                                        SGDTSPRHL
75                                                 SNVSSTGSIDM
76                                                 SNVSSTGSIDMV
77                                                 SNVSSTGSIDMV
78                                                 SNVSSTGSIDMV
79                                                 SNVSSTGSIDMV
80                                                 SNVSSTGSIDMV
81                                                 SNVSSTGSIDMV
82                                                 SNVSSTGSIDMV
83                                                 SNVSSTGSIDMV
84                                                 SNVSSTGSIDMV
85                                                 SNVSSTGSIDMV
86                                                 SNVSSTGSIDMV
87                                                 SNVSSTGSIDMV
88                                                    SSTGSIDMV
89                                                    SSTGSIDMV
90                                                    SSTGSIDMV
91                                                    SSTGSIDMV
92                                                    SSTGSIDMV

 1 DSPQLATLADEVSASLAKQGL
66 D
67 DSP
68 DSPQ
69 DSPQL
70 DSPQLA
71 DSPQLATL
77 D
78 DS
79 DSP
80 DSPQ
81 DSPQL
82 DSPQLA
```

83 DSPQLAT
84 DSPQLATL
85 DSPQLATLADEV
86 DSPQLATLADEVSA
87 DSPQLATLADEVSASLAKQGL
88 DSPQL
89 DSPQLA
90 DSPQLATL
91 DSPQLATLADEV
92 DSPQLATLADEVSASLAKQGL
93 LATLADEVSASLAKQGL
94 ATLADEVSASLAKQGL
95 TLADEVSASLAKQGL
96 LADEVSASLAKQGL
97 ADEVSASLAKQGL
98 EVSASLAKQGL
99 VSASLAKQGL
100 SASLAKQGL

Soluble Tau 10 min

```
  1 MAEPRQEFEVMEDHAGTYGLGDRKDQGGYTMHQDQEGDTDAGLKESPLQTPTEDGSEEPG

  1 SETSDAKSTPTAEDVTAPLVDEGAPGKQAAAQPHTEIPEGTTAEEAGIGDTPSLEDEAAG

  1 HVTQARMVSKSKDGTGSDDKKAKGADGKTKIATPRGAAPPGQKGQANATRIPAKTPPAPK

  1 TPPSSGEPPKSGDRSGYSSPGSPGTPGSRSRTPSLPTPPTREPKKVAVVRTPPKSPSSAK
  2                                               AVVRTPPKSPSSAK
  3                                               AVVRTPPKSPSSAK
  4                                                  RTPPKSPSSAK

  1 SRLQTAPVPMPDLKNVKSKIGSTENLKHQPGGGKVQIINKKLDLSNVQSKCGSKDNIKHV
  2 SRLQ
  3 SRLQTAPVPMPDLKNV
  4 SRLQTAPVPMPDLKNV
  5     TAPVPMPDLKNV
  6                 KSKIGSTENL
  7                 KSKIGSTENLKHQPGGGKVQI
  8                           KHQPGGGKVQI
  9                           KHQPGGGKVQII
 10                           KHQPGGGKVQIINKKLDLSNV
 11                           KHQPGGGKVQIINKKLDLSNVQSKC
 12                                    QIINKKLDLSNV
 13                                    QIINKKLDLSNVQSKC
 14                                      INKKLDLSNV
 15                                      INKKLDLSNVQS
 16                                      INKKLDLSNVQSKC
 17                                       NKKLDLSNV
 18                                       NKKLDLSNVQS
 19                                       NKKLDLSNVQSKC
 20                                         KLDLSNVQS
 21                                         KLDLSNVQSKC
 22                                           DLSNVQSKC
 23                                           DLSNVQSKCGSKDNIKHV
 24                                             SNVQSKCGSKDNIKHV
 25                                              NVQSKCGSKDNIKHV
 26                                                QSKCGSKDNIKHV
 27                                                  KCGSKDNIKHV
 28                                                    GSKDNIKHV
 29                                                    GSKDNIKHV
 30                                                    GSKDNIKHV
 31                                                    GSKDNIKHV
 32                                                        NIKHV

  1 PGGGSVQIVYKPVDLSKVTSKCGSLGNIHHKPGGGQVEVKSEKLDFKDRVQSKIGSLDNI
 23 PGGGSVQI
 24 PGGGSVQI
 25 PGGGSVQI
 26 PGGGSVQI
 27 PGGGSVQI
 28 PGGGSVQI
 29 PGGGSVQIV
 30 PGGGSVQIVYKPV
 31 PGGGSVQIVYKPVDLSKVTSKC
 32 PGGGSVQI
 33   GGSVQIVYKPVDLSKVTSKC
 34         VYKPVDLS
 35         VYKPVDLSKV
 36         VYKPVDLSKVT
 37         VYKPVDLSKVTSK
 38         VYKPVDLSKVTSKC
```

```
39         VYKPVDLSKVTSKCG
40         VYKPVDLSKVTSKCGSL
41         VYKPVDLSKVTSKCGSLGNI
42         VYKPVDLSKVTSKCGSLGNIH
43          YKPVDLSKV
44          YKPVDLSKVTSKC
45            PVDLSKVTSKC
46              DLSKVTSKC
47              DLSKVTSKCGSLGNI
48                   TSKCGSLGNI
49                       GSLGNIHHKPGGGQVEV
50                       GSLGNIHHKPGGGQVEVKS
51                       GSLGNIHHKPGGGQVEVKSEKL
52                             HHKPGGGQVEVKSEKL
53                                             DFKDRVQSKIGSL
54                                             DFKDRVQSKIGSLDNI
55                                                   QSKIGSLDNI

 1 THVPGGGNKKIETHKLTFRENAKAKTDHGAEIVYKSPVVSGDTSPRHLSNVSSTGSIDMV
56 THVPGGGNKKIETHKL
57    PGGGNKKIETHKL
58      GGNKKIETHKL
59                 TFRENAKAKTD
60                 TFRENAKAKTDH
61                 TFRENAKAKTDHGAEI
62                 TFRENAKAKTDHGAEIV
63                 TFRENAKAKTDHGAEIVY
64                 TFRENAKAKTDHGAEIVYK
65                 TFRENAKAKTDHGAEIVYKSPVVSGDTSPRHL
66                  FRENAKAKTDHGAEI
67                                 VYKSPVVSG
68                                 VYKSPVVSGD
69                                 VYKSPVVSGDT
70                                 VYKSPVVSGDTS
71                                 VYKSPVVSGDTSP
72                                 VYKSPVVSGDTSPR
73                                 VYKSPVVSGDTSPRH
74                                 VYKSPVVSGDTSPRHL
75                                 VYKSPVVSGDTSPRHLS
76                                 VYKSPVVSGDTSPRHLSN
77                                 VYKSPVVSGDTSPRHLSNV
78                                 VYKSPVVSGDTSPRHLSNVS
79                                 VYKSPVVSGDTSPRHLSNVSS
80                                 VYKSPVVSGDTSPRHLSNVSST
81                                 VYKSPVVSGDTSPRHLSNVSSTG
82                                 VYKSPVVSGDTSPRHLSNVSSTGS
83                                 VYKSPVVSGDTSPRHLSNVSSTGSID
84                                 VYKSPVVSGDTSPRHLSNVSSTGSIDM
85                                 VYKSPVVSGDTSPRHLSNVSSTGSIDMV
86                                 VYKSPVVSGDTSPRHLSNVSSTGSIDMV
87                                 VYKSPVVSGDTSPRHLSNVSSTGSIDMV
88                                 VYKSPVVSGDTSPRHLSNVSSTGSIDMV
89                                 VYKSPVVSGDTSPRHLSNVSSTGSIDMV
90                                 VYKSPVVSGDTSPRHLSNVSSTGSIDMV
91                                 VYKSPVVSGDTSPRHLSNVSSTGSIDMV
92                                 VYKSPVVSGDTSPRHLSNVSSTGSIDMV
93                                  YKSPVVSGDTSPRHL
94                                       VSGDTSPRHL
95                                       VSGDTSPRHLSNV
96                                        SGDTSPRHL
97                                                 SNVSSTGSIDM
98                                                 SNVSSTGSIDMV
99                                                 SNVSSTGSIDMV
```

```
100                                                  SNVSSTGSIDMV
101                                                  SNVSSTGSIDMV
102                                                  SNVSSTGSIDMV
103                                                  SNVSSTGSIDMV
104                                                  SNVSSTGSIDMV
105                                                  SNVSSTGSIDMV
106                                                  SNVSSTGSIDMV
107                                                  SNVSSTGSIDMV
108                                                  SNVSSTGSIDMV
109                                                  SNVSSTGSIDMV
110                                                  SNVSSTGSIDMV
111                                                  SNVSSTGSIDMV
112                                                     SSTGSIDMV
113                                                     SSTGSIDMV
114                                                     SSTGSIDMV
115                                                     SSTGSIDMV
116                                                     SSTGSIDMV
117                                                     SSTGSIDMV
118                                                        GSIDMV
119                                                        GSIDMV

  1 DSPQLATLADEVSASLAKQGL
 86 D
 87 DSP
 88 DSPQ
 89 DSPQL
 90 DSPQLA
 91 DSPQLAT
 92 DSPQLATL
 99 D
100 DS
101 DSP
102 DSPQ
103 DSPQL
104 DSPQLA
105 DSPQLAT
106 DSPQLATL
107 DSPQLATLAD
108 DSPQLATLADE
109 DSPQLATLADEV
110 DSPQLATLADEVSA
111 DSPQLATLADEVSASLAKQGL
112 DSPQL
113 DSPQLA
114 DSPQLATL
115 DSPQLATLADEV
116 DSPQLATLADEVSA
117 DSPQLATLADEVSASLAKQGL
118 DSPQLATL
119 DSPQLATLADEV
120 DSPQLATLADEV
121     LATLADEVSASLAKQGL
122      ATLADEVSASLAKQGL
123       TLADEVSASLAKQGL
124        LADEVSASLAKQGL
125         ADEVSASLAKQ
126         ADEVSASLAKQGL
127           EVSASLAKQGL
128            VSASLAKQGL
129             SASLAKQGL
```

Soluble Tau 120 min

```
 1 MAEPRQEFEVMEDHAGTYGLGDRKDQGGYTMHQDQEGDTDAGLKESPLQTPTEDGSEEPG
 2  AEPRQEFEV
 3  AEPRQEFEVM

 1 SETSDAKSTPTAEDVTAPLVDEGAPGKQAAAQPHTEIPEGTTAEEAGIGDTPSLEDEAAG
 4                                               GIGDTPSLEDEAAG

 1 HVTQARMVSKSKDGTGSDDKKAKGADGKTKIATPRGAAPPGQKGQANATRIPAKTPPAPK
 4 HVTQA

 1 TPPSSGEPPKSGDRSGYSSPGSPGTPGSRSRTPSLPTPPTREPKKVAVVRTPPKSPSSAK
 5                                               AVVRTPPKSPSSA
 6                                               AVVRTPPKSPSSAK
 7                                               AVVRTPPKSPSSAK
 8                                                 VRTPPKSPSSAK
 9                                                  RTPPKSPSSAK
10                                                  RTPPKSPSSAK
11                                                          SAK

 1 SRLQTAPVPMPDLKNVKSKIGSTENLKHQPGGGKVQIINKKLDLSNVQSKCGSKDNIKHV
 6 SRLQ
 7 SRLQTAPVPMPDLKNV
 8 SRLQTAPVPMPDLKNV
 9 SRLQ
10 SRLQTAPVPMPDLKNV
11 SRLQTAPVPMPDLKNV
12  RLQTAPVPMPDLKNV
13    QTAPVPMPDLKNV
14     TAPVPMPDLKNV
15                 KSKIGSTENL
16                 KSKIGSTENLKHQPGGGKV
17                 KSKIGSTENLKHQPGGGKVQI
18                 KSKIGSTENLKHQPGGGKVQII
19                 KSKIGSTENLKHQPGGGKVQIINK
20                 KSKIGSTENLKHQPGGGKVQIINKKL
21                 KSKIGSTENLKHQPGGGKVQIINKKLDLSNV
22                 KSKIGSTENLKHQPGGGKVQIINKKLDLSNVQS
23                        ENLKHQPGGGKVQIINKKLDLSNV
24                           KHQPGGGKVQI
25                           KHQPGGGKVQII
26                           KHQPGGGKVQIINK
27                           KHQPGGGKVQIINKKLDLSNV
28                           KHQPGGGKVQIINKKLDLSNVQS
29                           KHQPGGGKVQIINKKLDLSNVQSKC
30                                    QIINKKLDLSNV
31                                    QIINKKLDLSNVQSKC
32                                      INKKLDLSNV
33                                      INKKLDLSNVQS
34                                      INKKLDLSNVQSKC
35                                      INKKLDLSNVQSKCGSKDNIKHV
36                                       NKKLDLSNV
37                                       NKKLDLSNVQS
38                                       NKKLDLSNVQSKC
39                                       NKKLDLSNVQSKCGSKDNIKHV
40                                         KLDLSNVQS
41                                         KLDLSNVQSKC
42                                         KLDLSNVQSKCGSKDNIKHV
43                                           DLSNVQSKC
44                                           DLSNVQSKCGSKDNIKHV
45                                             SNVQSKCGSKDNIKHV
46                                              NVQSKCGSKDNIKHV
```

```
47                                                QSKCGSKDNIKHV
48                                                  KCGSKDNIKHV
49                                                    GSKDNIKHV
50                                                    GSKDNIKHV
51                                                    GSKDNIKHV
52                                                    GSKDNIKHV
53                                                    GSKDNIKHV
54                                                        NIKHV

 1 PGGGSVQIVYKPVDLSKVTSKCGSLGNIHHKPGGGQVEVKSEKLDFKDRVQSKIGSLDNI
35 PGGGSVQI
39 PGGGSVQI
42 PGGGSVQI
44 PGGGSVQI
45 PGGGSVQI
46 PGGGSVQI
47 PGGGSVQI
48 PGGGSVQI
49 PGGGSVQI
50 PGGGSVQIV
51 PGGGSVQIVYKPV
52 PGGGSVQIVYKPVDLSKV
53 PGGGSVQIVYKPVDLSKVTSKC
54 PGGGSVQI
55   GGSVQIVYKPVDLSKV
56   GGSVQIVYKPVDLSKVTSKC
57       QIVYKPVDLSKV
58       QIVYKPVDLSKVTSKC
59        IVYKPVDLSKVTSKC
60         VYKPVDLS
61         VYKPVDLSK
62         VYKPVDLSKV
63         VYKPVDLSKVT
64         VYKPVDLSKVTSK
65         VYKPVDLSKVTSKC
66         VYKPVDLSKVTSKCG
67         VYKPVDLSKVTSKCGS
68         VYKPVDLSKVTSKCGSL
69         VYKPVDLSKVTSKCGSLG
70         VYKPVDLSKVTSKCGSLGN
71         VYKPVDLSKVTSKCGSLGNI
72         VYKPVDLSKVTSKCGSLGNIH
73         VYKPVDLSKVTSKCGSLGNIHH
74         VYKPVDLSKVTSKCGSLGNIHHKPGGGQVEVKSEKL
75          YKPVDLSKV
76          YKPVDLSKVTSKC
77          YKPVDLSKVTSKCGSLGNI
78           KPVDLSKVTSKC
79            PVDLSKVTSKC
80              DLSKVTSKC
81              DLSKVTSKCGSLGNI
82               LSKVTSKC
83                SKVTSKCGSLGNI
84                   TSKCGSLGNI
85                       GSLGNIHHKPGG
86                       GSLGNIHHKPGGG
87                       GSLGNIHHKPGGGQVEV
88                       GSLGNIHHKPGGGQVEVKS
89                       GSLGNIHHKPGGGQVEVKSE
90                       GSLGNIHHKPGGGQVEVKSEK
91                       GSLGNIHHKPGGGQVEVKSEKL
92                       GSLGNIHHKPGGGQVEVKSEKLD
93                         LGNIHHKPGGGQVEVKSEKL
```

```
  94                          GNIHHKPGGGQVEVKSEKL
  95                             HHKPGGGQVEVKSEKL
  96                                      EVKSEKL
  97                                             DFKDRVQSKIGSL
  98                                             DFKDRVQSKIGSLDNI
  99                                             DFKDRVQSKIGSLDNI
 100                                             DFKDRVQSKIGSLDNI
 101                                                   QSKIGSLDNI
 102                                                   QSKIGSLDNI
 103                                                          DNI

   1 THVPGGGNKKIETHKLTFRENAKAKTDHGAEIVYKSPVVSGDTSPRHLSNVSSTGSIDMV
  99 T
 100 THVPGGGNKKIETHKL
 102 THVPGGGNKKIETHKL
 103 THVPGGGNKKIETHKL
 104 THVPGGGNKKIETHKL
 105    PGGGNKKIETHKL
 106     GGGNKKIETHKL
 107      GGNKKIETHKL
 108       GNKKIETHKL
 109                 TFRENAKAKTD
 110                 TFRENAKAKTDH
 111                 TFRENAKAKTDHGA
 112                 TFRENAKAKTDHGAEI
 113                 TFRENAKAKTDHGAEIV
 114                 TFRENAKAKTDHGAEIVY
 115                 TFRENAKAKTDHGAEIVYK
 116                 TFRENAKAKTDHGAEIVYKSPVVSG
 117                 TFRENAKAKTDHGAEIVYKSPVVSGDTSPRHL
 118                 TFRENAKAKTDHGAEIVYKSPVVSGDTSPRHLSNV
 119                  FRENAKAKTDHGAEI
 120                                 VYKSPVVSG
 121                                 VYKSPVVSGD
 122                                 VYKSPVVSGDT
 123                                 VYKSPVVSGDTS
 124                                 VYKSPVVSGDTSPR
 125                                 VYKSPVVSGDTSPRH
 126                                 VYKSPVVSGDTSPRHL
 127                                 VYKSPVVSGDTSPRHLS
 128                                 VYKSPVVSGDTSPRHLSN
 129                                 VYKSPVVSGDTSPRHLSNV
 130                                 VYKSPVVSGDTSPRHLSNVS
 131                                 VYKSPVVSGDTSPRHLSNVSS
 132                                 VYKSPVVSGDTSPRHLSNVSST
 133                                 VYKSPVVSGDTSPRHLSNVSSTG
 134                                 VYKSPVVSGDTSPRHLSNVSSTGS
 135                                 VYKSPVVSGDTSPRHLSNVSSTGSID
 136                                 VYKSPVVSGDTSPRHLSNVSSTGSIDM
 137                                 VYKSPVVSGDTSPRHLSNVSSTGSIDMV
 138                                 VYKSPVVSGDTSPRHLSNVSSTGSIDMV
 139                                 VYKSPVVSGDTSPRHLSNVSSTGSIDMV
 140                                 VYKSPVVSGDTSPRHLSNVSSTGSIDMV
 141                                 VYKSPVVSGDTSPRHLSNVSSTGSIDMV
 142                                 VYKSPVVSGDTSPRHLSNVSSTGSIDMV
 143                                 VYKSPVVSGDTSPRHLSNVSSTGSIDMV
 144                                 VYKSPVVSGDTSPRHLSNVSSTGSIDMV
 145                                  YKSPVVSGDTSPRHL
 146                                  YKSPVVSGDTSPRHLSNVSSTGSIDMV
 147                                       VSGDTSPRHL
 148                                       VSGDTSPRHLSNV
 149                                       VSGDTSPRHLSNVSSTGSIDMV
 150                                       VSGDTSPRHLSNVSSTGSIDMV
```

```
151                                        SGDTSPRHL
152                                                 SNVSSTGSIDM
153                                                 SNVSSTGSIDMV
154                                                 SNVSSTGSIDMV
155                                                 SNVSSTGSIDMV
156                                                 SNVSSTGSIDMV
157                                                 SNVSSTGSIDMV
158                                                 SNVSSTGSIDMV
159                                                 SNVSSTGSIDMV
160                                                 SNVSSTGSIDMV
161                                                 SNVSSTGSIDMV
162                                                 SNVSSTGSIDMV
163                                                 SNVSSTGSIDMV
164                                                 SNVSSTGSIDMV
165                                                 SNVSSTGSIDMV
166                                                    SSTGSIDMV
167                                                    SSTGSIDMV
168                                                    SSTGSIDMV
169                                                    SSTGSIDMV
170                                                    SSTGSIDMV
171                                                    SSTGSIDMV
172                                                       GSIDMV
173                                                       GSIDMV
174                                                       GSIDMV

  1 DSPQLATLADEVSASLAKQGL
138 D
139 DS
140 DSP
141 DSPQ
142 DSPQL
143 DSPQLA
144 DSPQLATL
146 DSPQLATL
149 DSPQLATL
150 DSPQLATLADEV
154 D
155 DS
156 DSP
157 DSPQ
158 DSPQL
159 DSPQLA
160 DSPQLATL
161 DSPQLATLAD
162 DSPQLATLADE
163 DSPQLATLADEV
164 DSPQLATLADEVSA
165 DSPQLATLADEVSASLAKQGL
166 DSPQL
167 DSPQLA
168 DSPQLATL
169 DSPQLATLADEV
170 DSPQLATLADEVSA
171 DSPQLATLADEVSASLAKQGL
172 DSPQLA
173 DSPQLATL
174 DSPQLATLADEV
175 DSPQLATLADEV
176  SPQLATLADEVSASLAKQGL
177     LATLADEVSASLAKQGL
178      ATLADEVSASLAKQGL
179       TLADEVSASLAKQGL
180        LADEVSASLAKQGL
```

181	ADEVSASLAKQ
182	ADEVSASLAKQG
183	ADEVSASLAKQGL
184	EVSASLAKQGL
185	VSASLAKQGL
186	SASLAKQGL

Supporting data 3. CLUSTAL O(1.2.4) multiple sequence alignment 2N4R tau

```
sp|P10637-2|MOUSE              MADPRQEFDTMEDHAG--------------DYTLLQDQEGDMDHGLKESPPQPPADDGAE     46
sp|P19332|RAT                  MAEPRQEFDTMEDQAG--------------DYTMLQDQEGDMDHGLKESPPQPPADDGSE     46
tr|F6UXK3|CALJA                MAEPRQEFNVMEDHTGTYGLED-------------QDQEGDTDTGLKESPLQTPAEDGSE     47
sp|O02828|CAPHI                MAEPRQEFDVMEDHA-------------QGDY-TLQDHEGDMEPGLKESPLQTPADDGSE     46
sp|P29172|BOVIN                MAEPRQEFDVMEDHA-------------QGDY-TLQDQEGDMDPGLKESPLQTPADDGSE     46
sp|P10636-8|HUMAN              MAEPRQEFEVMEDHAGTYGLGDRKD---QGGYTMHQDQEGDTDAGLKESPLQTPTEDGSE     57
tr|A0A2K6S2L5|SAIBB            MAEPRQEFDVMEDHAGTYGLEDQ-------------DQEGDTDTGLKESPLQTPAEDGSE     47
tr|A0A8P0SZT9|CANLF            MAEPRQEFTVMEDHAGTYG----KDLPSQGGYTLLQDHEGDVDHGLKESPLQTPADDGSE     56
tr|A0A7N5KFP8|AILME            MAEPRQEFTATEDHAGTYGMEERKDLPSQGGYTLLQDHEGDMDHGLKESPLQTPADDGSE     60
                               **:***** . **::                    *:*** : ****** * *::**:*

sp|P10637-2|MOUSE              EPGSETSDAKSTPTAEDVTAPLVDERAPDKQAAAQPHTEIPEGITAEEAGIGDTPNQEDQ     106
sp|P19332|RAT                  EPGSETSDAKSTPTAEDVTAPLVEERAPDKQATAQSHTEIPEGTTAEEAGIGDTPNMEDQ     106
tr|F6UXK3|CALJA                EPGSESSDAKSTPTVEDVTAPLVDERAPGKQAAAQPHTEIPEGTTAEEAGIGDTPTPEDQ     107
sp|O02828|CAPHI                EPGSETSDAKSTPTAE-----------------------------AEEAGIGDTSNLEDQ     77
sp|P29172|BOVIN                EPGSETSDAKSTPTAEDATAPLVDEGAPGEQAAAQAPAEIPEGTAAEEAGIGDTSNLEDQ     106
sp|P10636-8|HUMAN              EPGSETSDAKSTPTAEDVTAPLVDEGAPGKQAAAQPHTEIPEGTTAEEAGIGDTPSLEDE     117
tr|A0A2K6S2L5|SAIBB            EPGSETSDAKSTPTAEDVTAPLVDERAPGEQAAAQPHTEIPEGTTAEEAGIGDTPTPEDQ     107
tr|A0A8P0SZT9|CANLF            EPGSETSDAKSTPTAEDVTAPLVDEGTPGEQAAAQPPMEIPEGATAEEAGIGDTPNLEDQ     116
tr|A0A7N5KFP8|AILME            EPGSETSDAKSTPTAEDVTAPLVDEGAPGEQAAAQPHTEIPEGATAEEAGIGDTPSLEDQ     120
                               *****:********.*                              ********* . **:

sp|P10637-2|MOUSE              AAGHVTQARVA--SKDRTGNDEKKAKGADGKTGAKIATPRGAASPAQKGTSNATRIPAKT     164
sp|P19332|RAT                  AAGHVTQARVAGVSKDRTGNDEKKAKGADGKTGAKIATPRGAATPGQKGTSNATRIPAKT     166
tr|F6UXK3|CALJA                AAGHVTQARMVSKSKDGTGGDDKKAKGADGK--TKIATPRGTAPPGQKGQANATRIPAKT     165
sp|O02828|CAPHI                AAGHVTQARMVSKGKDGTGPDDKKAKGADGKPGTKIATPRGAAPPGQKGQANATRIPAKT     137
sp|P29172|BOVIN                AAGHVTQARMVSKGKDGTGPDDKKTKGADGKPGTKIATPRGAAPPGQKGQANATRIPAKT     166
sp|P10636-8|HUMAN              AAGHVTQARMVSKSKDGTGSDDKKAKGADGKT--KIATPRGAAPPGQKGQANATRIPAKT     175
tr|A0A2K6S2L5|SAIBB            AAGHVTQARMVSKSKDGTGGDDKKAKGADGKT--KIATPRGTAPPGQKGQANATRIPAKT     165
tr|A0A8P0SZT9|CANLF            AAGHVTQARMVSKGKDGTGTDDKKAKGADGKTGTKIATPRGATPSGQKGQANATRIPAKT     176
tr|A0A7N5KFP8|AILME            AAGHVTQARMVSKGKDGTGPEDKKAKGADGKTGTKIATPRGAAPPGQKGQANATRIPAKT     180
                               *********:.  .** ** ::**:******   *******::  .*** :*********

sp|P10637-2|MOUSE              TPSPKTPPG----------------SGEPPKSGERSGYSSPGSPGTPGSRSRTPSLPTPP     208
sp|P19332|RAT                  TPSPKTPPG----------------SGEPPKSGERSGYSSPGSPGTPGSRSRTPSLPTPP     210
tr|F6UXK3|CALJA                PPAPKTPPS----------------SGETPKSGDRSGYSSPGSPGTPGSRSRTPSLPTPP     209
sp|O02828|CAPHI                TPTPKTSPG----------------TGESGKSGDRSGYSSPGSPGTPGSRSRTPSLPTPP     181
sp|P29172|BOVIN                TPTPKTSPATMQVQKKPPPAGAKSERGESGKSGDRSGYSSPGSPGTPGSRSRTPSLPTPP     226
sp|P10636-8|HUMAN              PPAPKTPPS----------------SGEPPKSGDRSGYSSPGSPGTPGSRSRTPSLPTPP     219
tr|A0A2K6S2L5|SAIBB            PPTPKTPPG----------------SGETPKSGDRSGYSSPGSPGTPGSRSRTPSLPTPP     209
tr|A0A8P0SZT9|CANLF            TPSPKTPPG-----------------GESGKSGDRSGYSSPGSPGTPGSRSRTPSLPTPP     219
tr|A0A7N5KFP8|AILME            TPSPKTPPG------------------ESGKSGDRSGYSSPGSPGTPGSRSRTPSLPTPP     222
                                *:*** *.                 *  ***:**************************

sp|P10637-2|MOUSE              TREPKKVAVVRTPPKSPSASKSRLQTAPVPMPDLKNVRSKIGSTENLKHQPGGGKVQIIN     268
sp|P19332|RAT                  TREPKKVAVVRTPPKSPSASKSRLQTAPVPMPDLKNVRSKIGSTENLKHQPGGGKVQIIN     270
tr|F6UXK3|CALJA                TREPKKVAVVRTPPKSPSSTKSRLQTAPVPMPDLKNVKSKIGSTENLKHQPGGGKVQIIN     269
sp|O02828|CAPHI                TREPKKVAVVRTPPKSPSAAKSRLQAAPGPMPDLKNVKSKIGSTENLKHQPGGGKVQIIN     241
sp|P29172|BOVIN                TREPKKVAVVRTPPKSPSAAKSRLQAAPGPMPDLKNVKSKIGSTENLKHQPGGGKVQIIN     286
sp|P10636-8|HUMAN              TREPKKVAVVRTPPKSPSSAKSRLQTAPVPMPDLKNVKSKIGSTENLKHQPGGGKVQIIN     279
tr|A0A2K6S2L5|SAIBB            TREPKKVAVVRTPPKSPSSAKSRLQTAPVPMPDLKNVKSKIGSTENLKHQPGGGKVQIIN     269
tr|A0A8P0SZT9|CANLF            TREPKKVAVVRTPPKSPSAAKSRLQTAPVPMPDLKNVRSKIGSTENLKHQPGGGKVQIIN     279
tr|A0A7N5KFP8|AILME            TREPKKVAVVRTPPKSPSSAKSRLQTAPVPMPDLKNVRSKIGSTENLKHQPGGGKVQIIN     282
                               ******************::*****:** ********:**********************

sp|P10637-2|MOUSE              KKLDLSNVQSKCGSKDNIKHVPGGGSVQIVYKPVDLSKVTSKCGSLGNIHHKPGGGQVEV     328
sp|P19332|RAT                  KKLDLSNVQSKCGSKDNIKHVPGGGSVHIVYKPVDLSKVTSKCGSLGNIHHKPGGGQVEV     330
tr|F6UXK3|CALJA                KKLDLSNVQSKCGSKDNIKHVPGGGSVQIVYKPVDLSKVTSKCGSLGNIHHKPGGGQVEV     329
sp|O02828|CAPHI                KKLDLSNVQSKCGSKDNIKHVPGGGSVQIVYKPVDLSKVTSKCGSLGNIHHKPGGGQVEV     301
sp|P29172|BOVIN                KKLDLSNVQSKCGSKDNIKHVPGGGSVQIVYKPVDLSKVTSKCGSLGNIHHKPGGGQVEV     346
sp|P10636-8|HUMAN              KKLDLSNVQSKCGSKDNIKHVPGGGSVQIVYKPVDLSKVTSKCGSLGNIHHKPGGGQVEV     339
tr|A0A2K6S2L5|SAIBB            KKLDLSNVQSKCGSKDNIKHVPGGGSVQIVYKPVDLSKVTSKCGSLGNIHHKPGGGQVEV     329
tr|A0A8P0SZT9|CANLF            KKLDLSNVQSKCGSKDNIKHVPGGGSVQIVYKPVDLSKVTSKCGSLGNIHHKPGGGQVEV     339
tr|A0A7N5KFP8|AILME            KKLDLSNVQSKCGSKDNIKHVPGGGSVQIVYKPVDLSKVTSKCG----------GGQVEV     332
                               ***************************:****************          ******

sp|P10637-2|MOUSE              KSEKLDFKDRVQSKIGSLDNITHVPGGGNKKIETHKLTFRENAKAKTDHGAEIVYKSPVV     388
sp|P19332|RAT                  KSEKLDFKDRVQSKIGSLDNITHVPGGGNKKIETHKLTFRENAKAKTDHGAEIVYKSPVV     390
tr|F6UXK3|CALJA                KSEKLDFKDRVQSKIGSLDNITHVPGGGNKKIETHKLTFRENAKAKTDHGAEIVYKSPVV     389
sp|O02828|CAPHI                KSEKLDFKDRVQSKIGSLDNITHVPGGGNKKIETHKLTFRENAKAKTDHGAEIVYKSPVV     361
sp|P29172|BOVIN                KSEKLDFKDRVQSKIGSLDNITHVPGGGNKKIETHKLTFRENAKAKTDHGAEIVYKSPVV     406
sp|P10636-8|HUMAN              KSEKLDFKDRVQSKIGSLDNITHVPGGGNKKIETHKLTFRENAKAKTDHGAEIVYKSPVV     399
tr|A0A2K6S2L5|SAIBB            KSEKLDFKDRVQSKIGSLDNITHVPGGGNKKIETHKLTFRENAKAKTDHGAEIVYKSPVV     389
tr|A0A8P0SZT9|CANLF            KSEKLDFKDRVQSKIGSLDNITHVPGGGNKKIETHKLTFRENAKAKTDHGAEIVYKSPVV     399
tr|A0A7N5KFP8|AILME            KSEKLDFKDRVQSKIGSLDNITHVPGGGNKKIETHKLTFRENAKAKTDHGAEIVYKSPVV     392
                               ************************************************************

sp|P10637-2|MOUSE              SGDTSPRHLSNVSSTGSIDMVDSPQLATLADEVSASLAKQGL      430
sp|P19332|RAT                  SGDTSPRHLSNVSSTGSIDMVDSPQLATLADEVSASLAKQGL      432
tr|F6UXK3|CALJA                SGDTSPRHLSNVSSTGSIDMVDSPQLATLADEVSASLAKQGL      431
sp|O02828|CAPHI                SGDTSPRHLSNVSSTGSIDMVDSPQLATLADEVSASLAKQGL      403
sp|P29172|BOVIN                SGDTSPRHLSNVSSTGSIDMVDSPQLATLADEVSASLAKQGL      448
sp|P10636-8|HUMAN              SGDTSPRHLSNVSSTGSIDMVDSPQLATLADEVSASLAKQGL      441
tr|A0A2K6S2L5|SAIBB            SGDTSPRHLSNVSSTGSIDMADSAQLATLADEVSASLAKQGL      431
tr|A0A8P0SZT9|CANLF            SGDTSPRHLSNVSSTGSIDMVDSPQLATLADEVSASLAKQGL      441
tr|A0A7N5KFP8|AILME            SGDTSPRHLSNVSSTGSIDMVDSPQLATLADEVSASLAKQGL      434
                               ********************.** *****************
```

Supporting data 4. Peptides identified in tau fibrils digested by HTRA1

Tau fibrils (β-sheets highlighted in yellow) 15 sec

```
 1 MAEPRQEFEVMEDHAGTYGLGDRKDQGGYTMHQDQEGDTDAGLKESPLQTPTEDGSEEPG

 1 SETSDAKSTPTAEDVTAPLVDEGAPGKQAAAQPHTEIPEGTTAEEAGIGDTPSLEDEAAG

 1 HVTQARMVSKSKDGTGSDDKKAKGADGKTKIATPRGAAPPGQKGQANATRIPAKTPPAPK

 1 TPPSSGEPPKSGDRSGYSSPGSPGTPGSRSRTPSLPTPPTREPKKVAVVRTPPKSPSSAK

 1 SRLQTAPVPMPDLKNVKSKIGSTENLKHQPGGGKVQIINKKLDLSNVQSKCGSKDNIKHV
 2     TAPVPMPDLKNV
 3                 KSKIGSTENL

 1 PGGGSVQIVYKPVDLSKVTSKCGSLGNIHHKPGGGQVEVKSEKLDFKDRVQSKIGSLDNI
 4         VYKPVDLSKV

 1 THVPGGGNKKIETHKLTFRENAKAKTDHGAEIVYKSPVVSGDTSPRHLSNVSSTGSIDMV
 5 THVPGGGNKKIETHKL
 6                 TFRENAKA
 7                 TFRENAKAKTDH
 8                 TFRENAKAKTDHGAEI
 9                 TFRENAKAKTDHGAEIV
10                                 VYKSPVVSG
11                                 VYKSPVVSGD
12                                 VYKSPVVSGDT
13                                 VYKSPVVSGDTS
14                                 VYKSPVVSGDTSP
15                                 VYKSPVVSGDTSPRH
16                                 VYKSPVVSGDTSPRHL
17                                 VYKSPVVSGDTSPRHLSN
18                                 VYKSPVVSGDTSPRHLSNV
19                                                 SNVSSTGSIDM
20                                                 SNVSSTGSIDMV
21                                                 SNVSSTGSIDMV
22                                                 SNVSSTGSIDMV
23                                                 SNVSSTGSIDMV
24                                                 SNVSSTGSIDMV
25                                                 SNVSSTGSIDMV
26                                                 SNVSSTGSIDMV
27                                                 SNVSSTGSIDMV
28                                                 SNVSSTGSIDMV
29                                                    SSTGSIDMV
30                                                    SSTGSIDMV
31                                                    SSTGSIDMV
32                                                    SSTGSIDMV

 1 DSPQLATLADEVSASLAKQGL
20 D
21 DSP
22 DSPQ
23 DSPQL
24 DSPQLA
25 DSPQLATL
26 DSPQLATLADEV
27 DSPQLATLADEVSA
28 DSPQLATLADEVSASLAKQGL
29 DSPQL
30 DSPQLA
31 DSPQLATL
```

```
32 DSPQLATLADEVSASLAKQGL
33         ADEVSASLAKQG
34             SASLAKQGL
35              ASLAKQGL
```

Tau fibrils 1 min

```
 1 MAEPRQEFEVMEDHAGTYGLGDRKDQGGYTMHQDQEGDTDAGLKESPLQTPTEDGSEEPG

 1 SETSDAKSTPTAEDVTAPLVDEGAPGKQAAAQPHTEIPEGTTAEEAGIGDTPSLEDEAAG

 1 HVTQARMVSKSKDGTGSDDKKAKGADGKTKIATPRGAAPPGQKGQANATRIPAKTPPAPK

 1 TPPSSGEPPKSGDRSGYSSPGSPGTPGSRSRTPSLPTPPTREPKKVAVVRTPPKSPSSAK
 2                                               AVVRTPPKSPSSA
 3                                               AVVRTPPKSPSSAK
 4                                                  RTPPKSPSSAK

 1 SRLQTAPVPMPDLKNVKSKIGSTENLKHQPGGGKVQIINKKLDLSNVQSKCGSKDNIKHV
 3 SRLQTAPVPMPDLKNV
 4 SRLQTAPVPMPDLKNV
 5     TAPVPMPDLKNV
 6      APVPMPDLKNV
 7                 KSKIGSTENL
 8                           KHQPGGGKVQI
 9                           KHQPGGGKVQII
10                                      INKKLDLSNV
11                                       NKKLDLSNV
12                                                    GSKDNIKHV
13                                                          KHV

 1 PGGGSVQIVYKPVDLSKVTSKCGSLGNIHHKPGGGQVEVKSEKLDFKDRVQSKIGSLDNI
12 PGGGSVQI
13 PGGGSVQI
14         VYKPVDLSK
15         VYKPVDLSKV
16         VYKPVDLSKVT
17         VYKPVDLSKVTSK
18         VYKPVDLSKVTSKC
19          YKPVDLSKV
20                                             DFKDRVQSKIGSLDNI
21                                                   QSKIGSLDNI
22                                                          DNI

 1 THVPGGGNKKIETHKLTFRENAKAKTDHGAEIVYKSPVVSGDTSPRHLSNVSSTGSIDMV
22 THVPGGGNKKIETHKL
23 THVPGGGNKKIETHKL
24 THVPGGGNKKIETHKLT
25    PGGGNKKIETHKL
26      GGNKKIETHKL
27       GNKKIETHKL
28                 TFRENAKA
29                 TFRENAKAKTD
30                 TFRENAKAKTDH
31                 TFRENAKAKTDHGAE
32                 TFRENAKAKTDHGAEI
33                 TFRENAKAKTDHGAEIV
34                 TFRENAKAKTDHGAEIVY
35                 TFRENAKAKTDHGAEIVYK
36                 TFRENAKAKTDHGAEIVYKSPV
37                 TFRENAKAKTDHGAEIVYKSPVV
38                 TFRENAKAKTDHGAEIVYKSPVVS
39                 TFRENAKAKTDHGAEIVYKSPVVSG
40                 TFRENAKAKTDHGAEIVYKSPVVSGD
41                 TFRENAKAKTDHGAEIVYKSPVVSGDTS
42                 TFRENAKAKTDHGAEIVYKSPVVSGDTSP
43                 TFRENAKAKTDHGAEIVYKSPVVSGDTSPRH
44                 TFRENAKAKTDHGAEIVYKSPVVSGDTSPRHL
```

```
 45                 TFRENAKAKTDHGAEIVYKSPVVSGDTSPRHLSN
 46                 TFRENAKAKTDHGAEIVYKSPVVSGDTSPRHLSNV
 47                                 VYKSPVVS
 48                                 VYKSPVVSG
 49                                 VYKSPVVSGD
 50                                 VYKSPVVSGDT
 51                                 VYKSPVVSGDTS
 52                                 VYKSPVVSGDTSP
 53                                 VYKSPVVSGDTSPR
 54                                 VYKSPVVSGDTSPRH
 55                                 VYKSPVVSGDTSPRHL
 56                                 VYKSPVVSGDTSPRHLS
 57                                 VYKSPVVSGDTSPRHLSN
 58                                 VYKSPVVSGDTSPRHLSNV
 59                                 VYKSPVVSGDTSPRHLSNVS
 60                                 VYKSPVVSGDTSPRHLSNVSS
 61                                 VYKSPVVSGDTSPRHLSNVSST
 62                                 VYKSPVVSGDTSPRHLSNVSSTG
 63                                 VYKSPVVSGDTSPRHLSNVSSTGS
 64                                 VYKSPVVSGDTSPRHLSNVSSTGSID
 65                                 VYKSPVVSGDTSPRHLSNVSSTGSIDM
 66                                 VYKSPVVSGDTSPRHLSNVSSTGSIDMV
 67                                 VYKSPVVSGDTSPRHLSNVSSTGSIDMV
 68                                 VYKSPVVSGDTSPRHLSNVSSTGSIDMV
 69                                 VYKSPVVSGDTSPRHLSNVSSTGSIDMV
 70                                 VYKSPVVSGDTSPRHLSNVSSTGSIDMV
 71                                  YKSPVVSGDTSPRHL
 72                                  YKSPVVSGDTSPRHLSNV
 73                                       VSGDTSPRHL
 74                                       VSGDTSPRHLSNV
 75                                       VSGDTSPRHLSNVSSTGSIDMV
 76                                        SGDTSPRHL
 77                                         GDTSPRHLSNVSSTGSIDMV
 78                                                 SNVSSTGSIDM
 79                                                 SNVSSTGSIDMV
 80                                                 SNVSSTGSIDMV
 81                                                 SNVSSTGSIDMV
 82                                                 SNVSSTGSIDMV
 83                                                 SNVSSTGSIDMV
 84                                                 SNVSSTGSIDMV
 85                                                 SNVSSTGSIDMV
 86                                                 SNVSSTGSIDMV
 87                                                 SNVSSTGSIDMV
 88                                                 SNVSSTGSIDMV
 89                                                 SNVSSTGSIDMV
 90                                                 SNVSSTGSIDMV
 91                                                 SNVSSTGSIDMV
 92                                                 SNVSSTGSIDMV
 93                                                    SSTGSIDMV
 94                                                    SSTGSIDMV
 95                                                    SSTGSIDMV
 96                                                    SSTGSIDMV
 97                                                    SSTGSIDMV
 98                                                    SSTGSIDMV
 99                                                       GSIDMV
100                                                       GSIDMV

  1 DSPQLATLADEVSASLAKQGL
 66 D
 67 DSPQ
 68 DSPQL
 69 DSPQLA
 70 DSPQLATL
```

```
 75 DSPQLATL
 77 DSPQLATL
 80 D
 81 DS
 82 DSP
 83 DSPQ
 84 DSPQL
 85 DSPQLA
 86 DSPQLAT
 87 DSPQLATL
 88 DSPQLATLAD
 89 DSPQLATLADEV
 90 DSPQLATLADEVSA
 91 DSPQLATLADEVSAS
 92 DSPQLATLADEVSASLAKQGL
 93 DSPQ
 94 DSPQL
 95 DSPQLA
 96 DSPQLATL
 97 DSPQLATLADEV
 98 DSPQLATLADEVSASLAKQGL
 99 DSPQLATL
100 DSPQLATLADEVSASLAKQGL
101 DSPQLATLADEVSASLAKQGL
102     LATLADEVSASLAKQGL
103      ATLADEVSASLAKQGL
104       TLADEVSASLAKQGL
105        LADEVSASLAKQGL
106         ADEVSASLAK
107         ADEVSASLAKQG
108         ADEVSASLAKQGL
109             SASLAKQGL
110              ASLAKQGL
```

Tau fibrils 2 min

```
  1 MAEPRQEFEVMEDHAGTYGLGDRKDQGGYTMHQDQEGDTDAGLKESPLQTPTEDGSEEPG

  1 SETSDAKSTPTAEDVTAPLVDEGAPGKQAAAQPHTEIPEGTTAEEAGIGDTPSLEDEAAG

  1 HVTQARMVSKSKDGTGSDDKKAKGADGKTKIATPRGAAPPGQKGQANATRIPAKTPPAPK

  1 TPPSSGEPPKSGDRSGYSSPGSPGTPGSRSRTPSLPTPPTREPKKVAVVRTPPKSPSSAK
  2                                               AVVRTPPKSPSSAK
  3                                               AVVRTPPKSPSSAK
  4                                                  RTPPKSPSSAK
  5                                                          SAK
  6                                                            K

  1 SRLQTAPVPMPDLKNVKSKIGSTENLKHQPGGGKVQIINKKLDLSNVQSKCGSKDNIKHV
  2 SRLQ
  3 SRLQTAPVPMPDLKNV
  4 SRLQTAPVPMPDLKNV
  5 SRLQTAPVPMPDLKNV
  6 SRLQTAPVPMPDLKNV
  7  RLQTAPVPMPDLKNV
  8   LQTAPVPMPDLKNV
  9    QTAPVPMPDLKNV
 10     TAPVPMPDLKNV
 11      APVPMPDLKNV
 12                 KSKIGSTENL
 13                           KHQPGGGKVQI
 14                           KHQPGGGKVQII
 15                                      INKKLDLSNV
 16                                       NKKLDLSNV
 17                                                    GSKDNIKHV

  1 PGGGSVQIVYKPVDLSKVTSKCGSLGNIHHKPGGGQVEVKSEKLDFKDRVQSKIGSLDNI
 17 PGGGSVQI
 18         VYKPVDLSK
 19         VYKPVDLSKV
 20         VYKPVDLSKVT
 21         VYKPVDLSKVTSK
 22         VYKPVDLSKVTSKC
 23          YKPVDLSKV
 24                                             DFKDRVQSKIGSLDNI
 25                                                   QSKIGSLDNI
 26                                                    SKIGSLDNI
 27                                                          DNI

  1 THVPGGGNKKIETHKLTFRENAKAKTDHGAEIVYKSPVVSGDTSPRHLSNVSSTGSIDMV
 27 THVPGGGNKKIETHKL
 28 THVPGGGN
 29 THVPGGGNKKIETHKL
 30 THVPGGGNKKIETHKLT
 31    PGGGNKKIETHKL
 32      GGNKKIETHKL
 33       GNKKIETHKL
 34                 TFRENAKA
 35                 TFRENAKAKTD
 36                 TFRENAKAKTDH
 37                 TFRENAKAKTDHGAE
 38                 TFRENAKAKTDHGAEI
 39                 TFRENAKAKTDHGAEIV
 40                 TFRENAKAKTDHGAEIVY
 41                 TFRENAKAKTDHGAEIVYK
 42                 TFRENAKAKTDHGAEIVYKSPV
```

43	TFRENAKAKTDHGAEIVYKSPVVS
44	TFRENAKAKTDHGAEIVYKSPVVSGDTSPRHL
45	TFRENAKAKTDHGAEIVYKSPVVSGDTSPRHLSNV
46	VYKSPVVS
47	VYKSPVVSG
48	VYKSPVVSGD
49	VYKSPVVSGDT
50	VYKSPVVSGDTS
51	VYKSPVVSGDTSP
52	VYKSPVVSGDTSPR
53	VYKSPVVSGDTSPRH
54	VYKSPVVSGDTSPRHL
55	VYKSPVVSGDTSPRHLS
56	VYKSPVVSGDTSPRHLSN
57	VYKSPVVSGDTSPRHLSNV
58	VYKSPVVSGDTSPRHLSNVS
59	VYKSPVVSGDTSPRHLSNVSS
60	VYKSPVVSGDTSPRHLSNVSST
61	VYKSPVVSGDTSPRHLSNVSSTG
62	VYKSPVVSGDTSPRHLSNVSSTGSID
63	VYKSPVVSGDTSPRHLSNVSSTGSIDM
64	VYKSPVVSGDTSPRHLSNVSSTGSIDMV
65	VYKSPVVSGDTSPRHLSNVSSTGSIDMV
66	VYKSPVVSGDTSPRHLSNVSSTGSIDMV
67	VYKSPVVSGDTSPRHLSNVSSTGSIDMV
68	VYKSPVVSGDTSPRHLSNVSSTGSIDMV
69	VYKSPVVSGDTSPRHLSNVSSTGSIDMV
70	VYKSPVVSGDTSPRHLSNVSSTGSIDMV
71	YKSPVVSGDTSPRHL
72	YKSPVVSGDTSPRHLSNV
73	VSGDTSPRHL
74	VSGDTSPRHLSNV
75	VSGDTSPRHLSNVSSTGSIDMV
76	VSGDTSPRHLSNVSSTGSIDMV
77	SGDTSPRHL
78	GDTSPRHLSNVSSTGSIDMV
79	SNVSSTGSIDM
80	SNVSSTGSIDMV
81	SNVSSTGSIDMV
82	SNVSSTGSIDMV
83	SNVSSTGSIDMV
84	SNVSSTGSIDMV
85	SNVSSTGSIDMV
86	SNVSSTGSIDMV
87	SNVSSTGSIDMV
88	SNVSSTGSIDMV
89	SNVSSTGSIDMV
90	SNVSSTGSIDMV
91	SNVSSTGSIDMV
92	SNVSSTGSIDMV
93	SNVSSTGSIDMV
94	SNVSSTGSIDMV
95	SNVSSTGSIDMV
96	SSTGSIDMV
97	SSTGSIDMV
98	SSTGSIDMV
99	SSTGSIDMV
100	SSTGSIDMV
101	SSTGSIDMV
102	SSTGSIDMV
103	SSTGSIDMV
104	SSTGSIDMV
105	GSIDMV

```
106                                                       GSIDMV
107                                                       GSIDMV
108                                                       GSIDMV
109                                                           MV
110                                                            V

  1 DSPQLATLADEVSASLAKQGL
 65 D
 66 DSP
 67 DSPQ
 68 DSPQL
 69 DSPQLA
 70 DSPQLATL
 75 DSPQL
 76 DSPQLATL
 78 DSPQLATL
 81 D
 82 DS
 83 DSP
 84 DSPQ
 85 DSPQL
 86 DSPQLA
 87 DSPQLAT
 88 DSPQLATL
 89 DSPQLATLA
 90 DSPQLATLAD
 91 DSPQLATLADE
 92 DSPQLATLADEV
 93 DSPQLATLADEVSA
 94 DSPQLATLADEVSAS
 95 DSPQLATLADEVSASLAKQGL
 96 DSPQ
 97 DSPQL
 98 DSPQLA
 99 DSPQLAT
100 DSPQLATL
101 DSPQLATLA
102 DSPQLATLADEV
103 DSPQLATLADEVSA
104 DSPQLATLADEVSASLAKQGL
105 DSPQLA
106 DSPQLATL
107 DSPQLATLADEV
108 DSPQLATLADEVSASLAKQGL
109 DSPQLATL
110 DSPQLATLADEV
111 DSPQLATLADEV
112 DSPQLATLADEVSASLAKQGL
113     LATLADEVSASLAKQGL
114      ATLADEVSASLAKQGL
115       TLADEVSASLAKQGL
116        LADEVSASLAKQGL
117         ADEVSASLAK
118         ADEVSASLAKQG
119         ADEVSASLAKQGL
120             SASLAKQGL
121              ASLAKQGL
```

Tau fibrils 5 min

```
 1 MAEPRQEFEVMEDHAGTYGLGDRKDQGGYTMHQDQEGDTDAGLKESPLQTPTEDGSEEPG
 2  AEPRQEFEVMEDHAGTYGLGDRKDQGGYTMH

 1 SETSDAKSTPTAEDVTAPLVDEGAPGKQAAAQPHTEIPEGTTAEEAGIGDTPSLEDEAAG
 3                                               GIGDTPSLEDEAAG

 1 HVTQARMVSKSKDGTGSDDKKAKGADGKTKIATPRGAAPPGQKGQANATRIPAKTPPAPK
 3 HVTQA

 1 TPPSSGEPPKSGDRSGYSSPGSPGTPGSRSRTPSLPTPPTREPKKVAVVRTPPKSPSSAK
 4                                               AVVRTPPKSPS
 5                                               AVVRTPPKSPSSAK
 6                                               AVVRTPPKSPSSAK
 7                                               AVVRTPPKSPSSAK
 8                                                 VRTPPKSPSSAK
 9                                                  RTPPKSPSSAK
10                                                       SPSSAK
11                                                         SSAK
12                                                          SAK
13                                                            K

 1 SRLQTAPVPMPDLKNVKSKIGSTENLKHQPGGGKVQIINKKLDLSNVQSKCGSKDNIKHV
 5 S
 6 SRLQ
 7 SRLQTAPVPMPDLKNV
 8 SRLQTAPVPMPDLKNV
 9 SRLQTAPVPMPDLKNV
10 SRLQTAPVPMPDLKNV
11 SRLQTAPVPMPDLKNV
12 SRLQTAPVPMPDLKNV
13 SRLQTAPVPMPDLKNV
14  RLQTAPVPMPDLKNV
15  RLQTAPVPMPDLKNVKSKIGSTENL
16   LQTAPVPMPDLKNV
17    QTAPVPMPDLKNV
18     TAPVPMPDLKNV
19     TAPVPMPDLKNVKSKIGSTENL
20      APVPMPDLKNV
21                 KSKIGSTENL
22                 KSKIGSTENLKHQPGGGKVQI
23                 KSKIGSTENLKHQPGGGKVQII
24                           KHQPGGGKVQI
25                           KHQPGGGKVQII
26                           KHQPGGGKVQIINKKLDLS
27                           KHQPGGGKVQIINKKLDLSNV
28                                    QIINKKLDLSNV
29                                     IINKKLDLSNV
30                                      INKKLDLSNV
31                                       NKKLDLSNV
32                                                    GSKDNIKHV
33                                                          KHV
34                                                          KHV

 1 PGGGSVQIVYKPVDLSKVTSKCGSLGNIHHKPGGGQVEVKSEKLDFKDRVQSKIGSLDNI
32 PGGGSVQI
33 PGGGSVQI
34 PGGGSVQIVYKPVDLSKV
35         VYKPVDL
36         VYKPVDLS
37         VYKPVDLSK
38         VYKPVDLSKV
```

```
39         VYKPVDLSKVT
40         VYKPVDLSKVTS
41         VYKPVDLSKVTSK
42         VYKPVDLSKVTSKC
43          YKPVDLSKV
44                       GSLGNIHHKPGGGQVEV
45                       GSLGNIHHKPGGGQVEVKSEKL
46                             HHKPGGGQVEVKSEKL
47                                           KLDFKDRVQSKIGSLDNI
48                                             DFKDRVQSK
49                                             DFKDRVQSKIGSL
50                                             DFKDRVQSKIGSLDN
51                                             DFKDRVQSKIGSLDNI
52                                             DFKDRVQSKIGSLDNI
53                                             DFKDRVQSKIGSLDNI
54                                             DFKDRVQSKIGSLDNI
55                                             DFKDRVQSKIGSLDNI
56                                             DFKDRVQSKIGSLDNI
57                                                   QSKIGSLDNI
58                                                   QSKIGSLDNI
59                                                   QSKIGSLDNI
60                                                    SKIGSLDNI
61                                                       GSLDNI
62                                                        SLDNI
63                                                          DNI
64                                                          DNI

 1 THVPGGGNKKIETHKLTFRENAKAKTDHGAEIVYKSPVVSGDTSPRHLSNVSSTGSIDMV
52 T
53 TH
54 THVPG
55 THVPGGGN
56 THVPGGGNKKIETHKL
58 T
59 THVPGGGNKKIETHKL
61 THVPGGGNKKIETHKL
62 THVPGGGNKKIETHKL
63 THVPGGGNKKIETHKL
64 THVPGGGNKKIETHKLT
65 THVPGGGN
66 THVPGGGNKKIETH
67 THVPGGGNKKIETHK
68 THVPGGGNKKIETHKL
69 THVPGGGNKKIETHKLT
70    PGGGNKKIETHKL
71     GGGNKKIETHKL
72      GGNKKIETHKL
73                 TFRENAKAKTD
74                 TFRENAKAKTDH
75                 TFRENAKAKTDHGA
76                 TFRENAKAKTDHGAE
77                 TFRENAKAKTDHGAEI
78                 TFRENAKAKTDHGAEIV
79                 TFRENAKAKTDHGAEIVY
80                 TFRENAKAKTDHGAEIVYK
81                 TFRENAKAKTDHGAEIVYKS
82                 TFRENAKAKTDHGAEIVYKSPV
83                 TFRENAKAKTDHGAEIVYKSPVV
84                 TFRENAKAKTDHGAEIVYKSPVVS
85                 TFRENAKAKTDHGAEIVYKSPVVSG
86                 TFRENAKAKTDHGAEIVYKSPVVSGD
87                 TFRENAKAKTDHGAEIVYKSPVVSGDT
88                 TFRENAKAKTDHGAEIVYKSPVVSGDTS
```

89	TFRENAKAKTDHGAEIVYKSPVVSGDTSP
90	TFRENAKAKTDHGAEIVYKSPVVSGDTSPR
91	TFRENAKAKTDHGAEIVYKSPVVSGDTSPRH
92	TFRENAKAKTDHGAEIVYKSPVVSGDTSPRHL
93	TFRENAKAKTDHGAEIVYKSPVVSGDTSPRHLS
94	TFRENAKAKTDHGAEIVYKSPVVSGDTSPRHLSN
95	TFRENAKAKTDHGAEIVYKSPVVSGDTSPRHLSNV
96	TFRENAKAKTDHGAEIVYKSPVVSGDTSPRHLSNVS
97	KTDHGAEIVYKSPVVSGDTSPRHL
98	VYKSPVVSG
99	VYKSPVVSGD
100	VYKSPVVSGDT
101	VYKSPVVSGDTS
102	VYKSPVVSGDTSP
103	VYKSPVVSGDTSPR
104	VYKSPVVSGDTSPRH
105	VYKSPVVSGDTSPRHL
106	VYKSPVVSGDTSPRHLS
107	VYKSPVVSGDTSPRHLSN
108	VYKSPVVSGDTSPRHLSNV
109	VYKSPVVSGDTSPRHLSNVS
110	VYKSPVVSGDTSPRHLSNVSS
111	VYKSPVVSGDTSPRHLSNVSST
112	VYKSPVVSGDTSPRHLSNVSSTG
113	VYKSPVVSGDTSPRHLSNVSSTGS
114	VYKSPVVSGDTSPRHLSNVSSTGSID
115	VYKSPVVSGDTSPRHLSNVSSTGSIDM
116	VYKSPVVSGDTSPRHLSNVSSTGSIDMV
117	VYKSPVVSGDTSPRHLSNVSSTGSIDMV
118	VYKSPVVSGDTSPRHLSNVSSTGSIDMV
119	VYKSPVVSGDTSPRHLSNVSSTGSIDMV
120	VYKSPVVSGDTSPRHLSNVSSTGSIDMV
121	VYKSPVVSGDTSPRHLSNVSSTGSIDMV
122	VYKSPVVSGDTSPRHLSNVSSTGSIDMV
123	VYKSPVVSGDTSPRHLSNVSSTGSIDMV
124	VYKSPVVSGDTSPRHLSNVSSTGSIDMV
125	YKSPVVSGDTSPRHL
126	YKSPVVSGDTSPRHLSNV
127	YKSPVVSGDTSPRHLSNVSSTGSIDMV
128	KSPVVSGDTSPRHL
129	VSGDTSPRHL
130	VSGDTSPRHLSNV
131	VSGDTSPRHLSNVSSTGSIDMV
132	VSGDTSPRHLSNVSSTGSIDMV
133	VSGDTSPRHLSNVSSTGSIDMV
134	SGDTSPRHL
135	SGDTSPRHLSNV
136	GDTSPRHLSNVSSTGSIDMV
137	GDTSPRHLSNVSSTGSIDMV
138	SNVSSTGSIDM
139	SNVSSTGSIDMV
140	SNVSSTGSIDMV
141	SNVSSTGSIDMV
142	SNVSSTGSIDMV
143	SNVSSTGSIDMV
144	SNVSSTGSIDMV
145	SNVSSTGSIDMV
146	SNVSSTGSIDMV
147	SNVSSTGSIDMV
148	SNVSSTGSIDMV
149	SNVSSTGSIDMV
150	SNVSSTGSIDMV
151	SNVSSTGSIDMV

```
152                                              SNVSSTGSIDMV
153                                              SNVSSTGSIDMV
154                                              SNVSSTGSIDMV
155                                              SNVSSTGSIDMV
156                                                VSSTGSIDMV
157                                                 SSTGSIDMV
158                                                 SSTGSIDMV
159                                                 SSTGSIDMV
160                                                 SSTGSIDMV
161                                                 SSTGSIDMV
162                                                 SSTGSIDMV
163                                                 SSTGSIDMV
164                                                 SSTGSIDMV
165                                                 SSTGSIDMV
166                                                 SSTGSIDMV
167                                                    GSIDMV
168                                                    GSIDMV
169                                                    GSIDMV
170                                                    GSIDMV
171                                                        MV
172                                                        MV
173                                                         V

  1 DSPQLATLADEVSASLAKQGL
117 D
118 DS
119 DSP
120 DSPQ
121 DSPQL
122 DSPQLA
123 DSPQLAT
124 DSPQLATL
127 DSPQLATL
131 DSPQL
132 DSPQLATL
133 DSPQLATLADEV
136 DSPQLATL
137 DSPQLATLADEV
140 D
141 DS
142 DSP
143 DSPQ
144 DSPQL
145 DSPQLA
146 DSPQLAT
147 DSPQLATL
148 DSPQLATLA
149 DSPQLATLAD
150 DSPQLATLADE
151 DSPQLATLADEV
152 DSPQLATLADEVS
153 DSPQLATLADEVSA
154 DSPQLATLADEVSAS
155 DSPQLATLADEVSASLAKQGL
156 DSPQLATLADEVSASLAKQGL
157 DSPQ
158 DSPQL
159 DSPQLA
160 DSPQLAT
161 DSPQLATL
162 DSPQLATLA
163 DSPQLATLADEV
164 DSPQLATLADEVSA
```

165 DSPQLATLADEVSAS
166 DSPQLATLADEVSASLAKQGL
167 DSPQLA
168 DSPQLATL
169 DSPQLATLADEV
170 DSPQLATLADEVSASLAKQGL
171 DSPQLATL
172 DSPQLATLADEV
173 DSPQLATLADEV
174 DSPQLATLADEV
175 DSPQLATLADEVSASLAKQGL
176   LATLADEVSASLAKQGL
177   ATLADEVSASLAKQGL
178   TLADEVSASLAKQGL
179   LADEVSASLAKQGL
180   ADEVSASLAK
181   ADEVSASLAKQG
182   ADEVSASLAKQGL
183   SASLAKQGL
184   ASLAKQGL

Tau fibrils 10 min

```
 1 MAEPRQEFEVMEDHAGTYGLGDRKDQGGYTMHQDQEGDTDAGLKESPLQTPTEDGSEEPG
 2  AEPRQEFEVMEDHAGTYGL
 3  AEPRQEFEVMEDHAGTYGLGDRKDQGGYTMH

 1 SETSDAKSTPTAEDVTAPLVDEGAPGKQAAAQPHTEIPEGTTAEEAGIGDTPSLEDEAAG
 4                                               GIGDTPSLEDEAAG

 1 HVTQARMVSKSKDGTGSDDKKAKGADGKTKIATPRGAAPPGQKGQANATRIPAKTPPAPK
 4 HVTQA

 1 TPPSSGEPPKSGDRSGYSSPGSPGTPGSRSRTPSLPTPPTREPKKVAVVRTPPKSPSSAK
 5                                TPSLPTPPTREPKKV
 6                                               AVVRTPPKSPS
 7                                               AVVRTPPKSPSSAK
 8                                               AVVRTPPKSPSSAK
 9                                               AVVRTPPKSPSSAK
10                                                 VRTPPKSPSSAK
11                                                  RTPPKSPSSAK
12                                                       SPSSAK
13                                                         SSAK
14                                                          SAK
15                                                            K

 1 SRLQTAPVPMPDLKNVKSKIGSTENLKHQPGGGKVQIINKKLDLSNVQSKCGSKDNIKHV
 7 S
 8 SRLQ
 9 SRLQTAPVPMPDLKNV
10 SRLQTAPVPMPDLKNV
11 SRLQTAPVPMPDLKNV
12 SRLQTAPVPMPDLKNV
13 SRLQTAPVPMPDLKNV
14 SRLQTAPVPMPDLKNV
15 SRLQTAPVPMPDLKNV
16  RLQTAPVPMPDLKNV
17  RLQTAPVPMPDLKNVKSKIGSTENL
18   LQTAPVPMPDLKNV
19    QTAPVPMPDLKNV
20     TAPVPMPDLKNV
21     TAPVPMPDLKNVKSKIGSTENL
22      APVPMPDLKNV
23        VPMPDLKNV
24                 KSKIGSTENL
25                 KSKIGSTENLKHQPGGGKVQI
26                 KSKIGSTENLKHQPGGGKVQII
27                           KHQPGGGKVQI
28                           KHQPGGGKVQII
29                           KHQPGGGKVQIINKKLDLS
30                           KHQPGGGKVQIINKKLDLSNV
31                           KHQPGGGKVQIINKKLDLSNVQS
32                                    QIINKKLDLSNV
33                                     IINKKLDLSNV
34                                      INKKLDLSNV
35                                      INKKLDLSNVQS
36                                       NKKLDLSNV
37                                       NKKLDLSNVQSKC
38                                                    GSKDNIKHV
39                                                          KHV
40                                                          KHV

 1 PGGGSVQIVYKPVDLSKVTSKCGSLGNIHHKPGGGQVEVKSEKLDFKDRVQSKIGSLDNI
38 PGGGSVQI
```

```
39 PGGGSVQI
40 PGGGSVQIVYKPVDLSKV
41         VYKPVDL
42         VYKPVDLS
43         VYKPVDLSK
44         VYKPVDLSKV
45         VYKPVDLSKVT
46         VYKPVDLSKVTS
47         VYKPVDLSKVTSK
48         VYKPVDLSKVTSKC
49          YKPVDLSKV
50                       GSLGNIHHKPGGGQVEV
51                       GSLGNIHHKPGGGQVEVKSEKL
52                          GNIHHKPGGGQVEVKSEKL
53                             HHKPGGGQVEVKSEKL
54                                        KSEKLDFKDRVQSKIGSLDNI
55                                           KLDFKDRVQSKIGSLDNI
56                                             DFKDRVQSK
57                                             DFKDRVQSKIGS
58                                             DFKDRVQSKIGSL
59                                             DFKDRVQSKIGSLDN
60                                             DFKDRVQSKIGSLDNI
61                                             DFKDRVQSKIGSLDNI
62                                             DFKDRVQSKIGSLDNI
63                                             DFKDRVQSKIGSLDNI
64                                             DFKDRVQSKIGSLDNI
65                                             DFKDRVQSKIGSLDNI
66                                             DFKDRVQSKIGSLDNI
67                                             DFKDRVQSKIGSLDNI
68                                             DFKDRVQSKIGSLDNI
69                                                  VQSKIGSLDNI
70                                                   QSKIGSLDNI
71                                                   QSKIGSLDNI
72                                                   QSKIGSLDNI
73                                                    SKIGSLDNI
74                                                    SKIGSLDNI
75                                                     KIGSLDNI
76                                                       GSLDNI
77                                                        SLDNI
78                                                          DNI
79                                                          DNI

 1 THVPGGGNKKIETHKLTFRENAKAKTDHGAEIVYKSPVVSGDTSPRHLSNVSSTGSIDMV
61 T
62 TH
63 THVPG
64 THVPGG
65 THVPGGG
66 THVPGGGN
67 THVPGGGNKKIETHKL
68 THVPGGGNKKIETHKLT
71 T
72 THVPGGGNKKIETHKL
74 THVPGGGNKKIETHKL
75 THVPGGGNKKIETHKL
76 THVPGGGNKKIETHKL
77 THVPGGGNKKIETHKL
78 THVPGGGNKKIETHKL
79 THVPGGGNKKIETHKLT
80 THVPGGGNKKIETHK
81 THVPGGGNKKIETHKL
82 THVPGGGNKKIETHKLT
83 THVPGGGNKKIETHKLTFRENA
```

```
 84    PGGGNKKIETHKL
 85     GGGNKKIETHKL
 86      GGNKKIETHKL
 87       GNKKIETHKL
 88                 TFRENAKAKTD
 89                 TFRENAKAKTDH
 90                 TFRENAKAKTDHGAE
 91                 TFRENAKAKTDHGAEI
 92                 TFRENAKAKTDHGAEIV
 93                 TFRENAKAKTDHGAEIVY
 94                 TFRENAKAKTDHGAEIVYK
 95                 TFRENAKAKTDHGAEIVYKS
 96                 TFRENAKAKTDHGAEIVYKSPV
 97                 TFRENAKAKTDHGAEIVYKSPVV
 98                 TFRENAKAKTDHGAEIVYKSPVVS
 99                 TFRENAKAKTDHGAEIVYKSPVVSG
100                 TFRENAKAKTDHGAEIVYKSPVVSGD
101                 TFRENAKAKTDHGAEIVYKSPVVSGDT
102                 TFRENAKAKTDHGAEIVYKSPVVSGDTS
103                 TFRENAKAKTDHGAEIVYKSPVVSGDTSP
104                 TFRENAKAKTDHGAEIVYKSPVVSGDTSPR
105                 TFRENAKAKTDHGAEIVYKSPVVSGDTSPRH
106                 TFRENAKAKTDHGAEIVYKSPVVSGDTSPRHL
107                 TFRENAKAKTDHGAEIVYKSPVVSGDTSPRHLS
108                 TFRENAKAKTDHGAEIVYKSPVVSGDTSPRHLSN
109                 TFRENAKAKTDHGAEIVYKSPVVSGDTSPRHLSNV
110                 TFRENAKAKTDHGAEIVYKSPVVSGDTSPRHLSNVS
111                         KTDHGAEIVYKSPVVSGDTSPRHL
112                                 VYKSPVVSG
113                                 VYKSPVVSGD
114                                 VYKSPVVSGDT
115                                 VYKSPVVSGDTS
116                                 VYKSPVVSGDTSP
117                                 VYKSPVVSGDTSPR
118                                 VYKSPVVSGDTSPRH
119                                 VYKSPVVSGDTSPRHL
120                                 VYKSPVVSGDTSPRHLS
121                                 VYKSPVVSGDTSPRHLSN
122                                 VYKSPVVSGDTSPRHLSNV
123                                 VYKSPVVSGDTSPRHLSNVS
124                                 VYKSPVVSGDTSPRHLSNVSST
125                                 VYKSPVVSGDTSPRHLSNVSSTG
126                                 VYKSPVVSGDTSPRHLSNVSSTGS
127                                 VYKSPVVSGDTSPRHLSNVSSTGSID
128                                 VYKSPVVSGDTSPRHLSNVSSTGSIDM
129                                 VYKSPVVSGDTSPRHLSNVSSTGSIDMV
130                                 VYKSPVVSGDTSPRHLSNVSSTGSIDMV
131                                 VYKSPVVSGDTSPRHLSNVSSTGSIDMV
132                                 VYKSPVVSGDTSPRHLSNVSSTGSIDMV
133                                 VYKSPVVSGDTSPRHLSNVSSTGSIDMV
134                                 VYKSPVVSGDTSPRHLSNVSSTGSIDMV
135                                 VYKSPVVSGDTSPRHLSNVSSTGSIDMV
136                                 VYKSPVVSGDTSPRHLSNVSSTGSIDMV
137                                 VYKSPVVSGDTSPRHLSNVSSTGSIDMV
138                                  YKSPVVSGDTSPRHL
139                                  YKSPVVSGDTSPRHLSNV
140                                  YKSPVVSGDTSPRHLSNVSSTGSIDMV
141                                   KSPVVSGDTSPRHL
142                                       VSGDTSPRHL
143                                       VSGDTSPRHLSNV
144                                       VSGDTSPRHLSNVSSTGSIDMV
145                                       VSGDTSPRHLSNVSSTGSIDMV
146                                       VSGDTSPRHLSNVSSTGSIDMV
```

```
147                                       VSGDTSPRHLSNVSSTGSIDMV
148                                        SGDTSPRHL
149                                        SGDTSPRHLSNV
150                                         GDTSPRHLSNVSSTGSIDMV
151                                         GDTSPRHLSNVSSTGSIDMV
152                                         GDTSPRHLSNVSSTGSIDMV
153                                                 SNVSSTGSIDM
154                                                 SNVSSTGSIDMV
155                                                 SNVSSTGSIDMV
156                                                 SNVSSTGSIDMV
157                                                 SNVSSTGSIDMV
158                                                 SNVSSTGSIDMV
159                                                 SNVSSTGSIDMV
160                                                 SNVSSTGSIDMV
161                                                 SNVSSTGSIDMV
162                                                 SNVSSTGSIDMV
163                                                 SNVSSTGSIDMV
164                                                 SNVSSTGSIDMV
165                                                 SNVSSTGSIDMV
166                                                 SNVSSTGSIDMV
167                                                 SNVSSTGSIDMV
168                                                 SNVSSTGSIDMV
169                                                 SNVSSTGSIDMV
170                                                 SNVSSTGSIDMV
171                                                   VSSTGSIDMV
172                                                    SSTGSIDMV
173                                                    SSTGSIDMV
174                                                    SSTGSIDMV
175                                                    SSTGSIDMV
176                                                    SSTGSIDMV
177                                                    SSTGSIDMV
178                                                    SSTGSIDMV
179                                                    SSTGSIDMV
180                                                    SSTGSIDMV
181                                                       GSIDMV
182                                                       GSIDMV
183                                                       GSIDMV
184                                                       GSIDMV
185                                                           MV
186                                                           MV
187                                                            V

  1 DSPQLATLADEVSASLAKQGL
130 D
131 DS
132 DSP
133 DSPQ
134 DSPQL
135 DSPQLA
136 DSPQLAT
137 DSPQLATL
140 DSPQLATL
144 DSPQL
145 DSPQLA
146 DSPQLATL
147 DSPQLATLADEV
150 DSPQLA
151 DSPQLATL
152 DSPQLATLADEV
155 D
156 DS
157 DSP
158 DSPQ
```

159 DSPQL
160 DSPQLA
161 DSPQLAT
162 DSPQLATL
163 DSPQLATLA
164 DSPQLATLAD
165 DSPQLATLADE
166 DSPQLATLADEV
167 DSPQLATLADEVS
168 DSPQLATLADEVSA
169 DSPQLATLADEVSAS
170 DSPQLATLADEVSASLAKQGL
171 DSPQLATLADEVSASLAKQGL
172 DSPQ
173 DSPQL
174 DSPQLA
175 DSPQLAT
176 DSPQLATL
177 DSPQLATLADEV
178 DSPQLATLADEVSA
179 DSPQLATLADEVSAS
180 DSPQLATLADEVSASLAKQGL
181 DSPQLA
182 DSPQLATL
183 DSPQLATLADEV
184 DSPQLATLADEVSASLAKQGL
185 DSPQLATL
186 DSPQLATLADEV
187 DSPQLATLADEV
188 DSPQLATLADEV
189 DSPQLATLADEVSASLAKQGL
190 LATLADEVSASLAKQGL
191 ATLADEVSASLAKQGL
192 TLADEVSASLAKQGL
193 LADEVSASLAKQGL
194 ADEVSASLAK
195 ADEVSASLAKQG
196 ADEVSASLAKQGL
197 SASLAKQGL
198 ASLAKQGL

Tau fibrils 120 min

```
  1 MAEPRQEFEVMEDHAGTYGLGDRKDQGGYTMHQDQEGDTDAGLKESPLQTPTEDGSEEPG

  1 SETSDAKSTPTAEDVTAPLVDEGAPGKQAAAQPHTEIPEGTTAEEAGIGDTPSLEDEAAG
  2                                               GIGDTPSLEDEAAG
  3                                                     SLEDEAAG

  1 HVTQARMVSKSKDGTGSDDKKAKGADGKTKIATPRGAAPPGQKGQANATRIPAKTPPAPK
  2 HVTQA
  3 HVTQA

  1 TPPSSGEPPKSGDRSGYSSPGSPGTPGSRSRTPSLPTPPTREPKKVAVVRTPPKSPSSAK
  4                            SRSRTPSLPTPPTREPKKV
  5                                TPSLPTPPTREPKKV
  6                                               AVVRTPPKSPS
  7                                               AVVRTPPKSPSSA
  8                                               AVVRTPPKSPSSAK
  9                                               AVVRTPPKSPSSAK
 10                                               AVVRTPPKSPSSAK
 11                                               AVVRTPPKSPSSAK
 12                                                VVRTPPKSPSSAK
 13                                                 VRTPPKSPSSAK
 14                                                  RTPPKSPSSAK
 15                                                       SPSSAK
 16                                                         SSAK
 17                                                          SAK
 18                                                          SAK
 19                                                            K

  1 SRLQTAPVPMPDLKNVKSKIGSTENLKHQPGGGKVQIINKKLDLSNVQSKCGSKDNIKHV
  8 S
  9 SRLQ
 10 SRLQT
 11 SRLQTAPVPMPDLKNV
 12 SRLQTAPVPMPDLKNV
 13 SRLQTAPVPMPDLKNV
 14 SRLQTAPVPMPDLKNV
 15 SRLQTAPVPMPDLKNV
 16 SRLQTAPVPMPDLKNV
 17 SRLQTAPVPMPDLKNV
 18 SRLQTAPVPMPDLKNVKSKIGST
 19 SRLQTAPVPMPDLKNV
 20 SRLQTAPVPMPDLKNV
 21  RLQTAPVPMPDLKNV
 22  RLQTAPVPMPDLKNVKSKIGSTENL
 23   LQTAPVPMPDLKNV
 24    QTAPVPMPDLKNV
 25     TAPVPMPDLKNV
 26     TAPVPMPDLKNVKSKIGST
 27     TAPVPMPDLKNVKSKIGSTENL
 28      APVPMPDLKNV
 29       PVPMPDLKNV
 30        VPMPDLKNV
 31                 KSKIGSTENL
 32                 KSKIGSTENLKHQPGGGKVQI
 33                 KSKIGSTENLKHQPGGGKVQII
 34                        ENLKHQPGGGKVQI
 35                        ENLKHQPGGGKVQII
 36                           KHQPGGGKVQI
 37                           KHQPGGGKVQII
 38                           KHQPGGGKVQIINKKLDLS
 39                           KHQPGGGKVQIINKKLDLSNV
```

```
40                           KHQPGGGKVQIINKKLDLSNVQS
41                                    QIINKKLDLSNV
42                                     IINKKLDLSNV
43                                      INKKLDLSNV
44                                      INKKLDLSNVQS
45                                       NKKLDLSNV
46                                       NKKLDLSNVQS
47                                       NKKLDLSNVQSKC
48                                                    GSKDNIKHV
49                                                          KHV
50                                                          KHV

 1 PGGGSVQIVYKPVDLSKVTSKCGSLGNIHHKPGGGQVEVKSEKLDFKDRVQSKIGSLDNI
48 PGGGSVQI
49 PGGGSVQI
50 PGGGSVQIVYKPVDLSKV
51         VYKPVDL
52         VYKPVDLS
53         VYKPVDLSK
54         VYKPVDLSKV
55         VYKPVDLSKVT
56         VYKPVDLSKVTS
57         VYKPVDLSKVTSK
58         VYKPVDLSKVTSKC
59          YKPVDLSKV
60                       GSLGNIHHKPGGGQVEV
61                       GSLGNIHHKPGGGQVEVKSEKL
62                          GNIHHKPGGGQVEVKSEKL
63                             HHKPGGGQVEVKSEKL
64                                        KSEKLDFKDRVQSKIGSLDNI
65                                           KLDFKDRVQSKIGSLDNI
66                                             DFKDRVQSKIGS
67                                             DFKDRVQSKIGSL
68                                             DFKDRVQSKIGSLD
69                                             DFKDRVQSKIGSLDN
70                                             DFKDRVQSKIGSLDNI
71                                             DFKDRVQSKIGSLDNI
72                                             DFKDRVQSKIGSLDNI
73                                             DFKDRVQSKIGSLDNI
74                                             DFKDRVQSKIGSLDNI
75                                             DFKDRVQSKIGSLDNI
76                                             DFKDRVQSKIGSLDNI
77                                                   QSKIGSLDNI
78                                                   QSKIGSLDNI
79                                                   QSKIGSLDNI
80                                                   QSKIGSLDNI
81                                                    SKIGSLDNI
82                                                    SKIGSLDNI
83                                                     KIGSLDNI
84                                                       GSLDNI
85                                                        SLDNI
86                                                          DNI
87                                                          DNI

 1 THVPGGGNKKIETHKLTFRENAKAKTDHGAEIVYKSPVVSGDTSPRHLSNVSSTGSIDMV
71 T
72 TH
73 THV
74 THVPG
75 THVPGGGN
76 THVPGGGNKKIETHKL
78 T
79 TH
```

```
 80 THVPGGGNKKIETHKL
 82 THVPGGGNKKIETHKL
 83 THVPGGGNKKIETHKL
 84 THVPGGGNKKIETHKL
 85 THVPGGGNKKIETHKL
 86 THVPGGGNKKIETHKL
 87 THVPGGGNKKIETHKLT
 88 THVPGGGN
 89 THVPGGGNKKIETH
 90 THVPGGGNKKIETHK
 91 THVPGGGNKKIETHKL
 92 THVPGGGNKKIETHKLT
 93    PGGGNKKIETHKL
 94     GGGNKKIETHKL
 95      GGNKKIETHKL
 96       GNKKIETHKL
 97                 TFRENAKAKTD
 98                 TFRENAKAKTDH
 99                 TFRENAKAKTDHGA
100                 TFRENAKAKTDHGAE
101                 TFRENAKAKTDHGAEI
102                 TFRENAKAKTDHGAEIV
103                 TFRENAKAKTDHGAEIVY
104                 TFRENAKAKTDHGAEIVYK
105                 TFRENAKAKTDHGAEIVYKS
106                 TFRENAKAKTDHGAEIVYKSPV
107                 TFRENAKAKTDHGAEIVYKSPVV
108                 TFRENAKAKTDHGAEIVYKSPVVS
109                 TFRENAKAKTDHGAEIVYKSPVVSG
110                 TFRENAKAKTDHGAEIVYKSPVVSGD
111                 TFRENAKAKTDHGAEIVYKSPVVSGDT
112                 TFRENAKAKTDHGAEIVYKSPVVSGDTS
113                 TFRENAKAKTDHGAEIVYKSPVVSGDTSP
114                 TFRENAKAKTDHGAEIVYKSPVVSGDTSPR
115                 TFRENAKAKTDHGAEIVYKSPVVSGDTSPRH
116                 TFRENAKAKTDHGAEIVYKSPVVSGDTSPRHL
117                 TFRENAKAKTDHGAEIVYKSPVVSGDTSPRHLS
118                 TFRENAKAKTDHGAEIVYKSPVVSGDTSPRHLSN
119                 TFRENAKAKTDHGAEIVYKSPVVSGDTSPRHLSNV
120                 TFRENAKAKTDHGAEIVYKSPVVSGDTSPRHLSNVS
121                         KTDHGAEIVYKSPVVSGDTSPRHL
122                                 VYKSPVVS
123                                 VYKSPVVSG
124                                 VYKSPVVSGD
125                                 VYKSPVVSGDT
126                                 VYKSPVVSGDTS
127                                 VYKSPVVSGDTSP
128                                 VYKSPVVSGDTSPR
129                                 VYKSPVVSGDTSPRH
130                                 VYKSPVVSGDTSPRHL
131                                 VYKSPVVSGDTSPRHLS
132                                 VYKSPVVSGDTSPRHLSN
133                                 VYKSPVVSGDTSPRHLSNV
134                                 VYKSPVVSGDTSPRHLSNVS
135                                 VYKSPVVSGDTSPRHLSNVSS
136                                 VYKSPVVSGDTSPRHLSNVSST
137                                 VYKSPVVSGDTSPRHLSNVSSTG
138                                 VYKSPVVSGDTSPRHLSNVSSTGSID
139                                 VYKSPVVSGDTSPRHLSNVSSTGSIDM
140                                 VYKSPVVSGDTSPRHLSNVSSTGSIDMV
141                                 VYKSPVVSGDTSPRHLSNVSSTGSIDMV
142                                 VYKSPVVSGDTSPRHLSNVSSTGSIDMV
143                                 VYKSPVVSGDTSPRHLSNVSSTGSIDMV
```

```
144    VYKSPVVSGDTSPRHLSNVSSTGSIDMV
145    VYKSPVVSGDTSPRHLSNVSSTGSIDMV
146    VYKSPVVSGDTSPRHLSNVSSTGSIDMV
147    VYKSPVVSGDTSPRHLSNVSSTGSIDMV
148    VYKSPVVSGDTSPRHLSNVSSTGSIDMV
149     YKSPVVSGDTSPRHL
150     YKSPVVSGDTSPRHLSNV
151     YKSPVVSGDTSPRHLSNVSSTGSIDMV
152      KSPVVSGDTSPRHL
153          VSGDTSPRHL
154          VSGDTSPRHLSNV
155          VSGDTSPRHLSNVSSTGSIDMV
156          VSGDTSPRHLSNVSSTGSIDMV
157          VSGDTSPRHLSNVSSTGSIDMV
158          VSGDTSPRHLSNVSSTGSIDMV
159           SGDTSPRHL
160           SGDTSPRHLSNV
161            GDTSPRHLSNVSSTGSIDMV
162            GDTSPRHLSNVSSTGSIDMV
163            GDTSPRHLSNVSSTGSIDMV
164                    SNVSSTGSIDM
165                    SNVSSTGSIDMV
166                    SNVSSTGSIDMV
167                    SNVSSTGSIDMV
168                    SNVSSTGSIDMV
169                    SNVSSTGSIDMV
170                    SNVSSTGSIDMV
171                    SNVSSTGSIDMV
172                    SNVSSTGSIDMV
173                    SNVSSTGSIDMV
174                    SNVSSTGSIDMV
175                    SNVSSTGSIDMV
176                    SNVSSTGSIDMV
177                    SNVSSTGSIDMV
178                    SNVSSTGSIDMV
179                    SNVSSTGSIDMV
180                    SNVSSTGSIDMV
181                    SNVSSTGSIDMV
182                       SSTGSIDMV
183                       SSTGSIDMV
184                       SSTGSIDMV
185                       SSTGSIDMV
186                       SSTGSIDMV
187                       SSTGSIDMV
188                       SSTGSIDMV
189                       SSTGSIDMV
190                          GSIDMV
191                          GSIDMV
192                          GSIDMV
193                          GSIDMV
194                              MV
195                               V
```

```
  1 DSPQLATLADEVSASLAKQGL
141 D
142 DS
143 DSP
144 DSPQ
145 DSPQL
146 DSPQLA
147 DSPQLAT
148 DSPQLATL
151 DSPQLATL
```

```
155 DSPQL
156 DSPQLA
157 DSPQLATL
158 DSPQLATLADEV
161 DSPQLA
162 DSPQLATL
163 DSPQLATLADEV
166 D
167 DS
168 DSP
169 DSPQ
170 DSPQL
171 DSPQLA
172 DSPQLAT
173 DSPQLATL
174 DSPQLATLA
175 DSPQLATLAD
176 DSPQLATLADE
177 DSPQLATLADEV
178 DSPQLATLADEVS
179 DSPQLATLADEVSA
180 DSPQLATLADEVSAS
181 DSPQLATLADEVSASLAKQGL
183 DSPQL
184 DSPQLA
185 DSPQLATL
186 DSPQLATLADEV
187 DSPQLATLADEVSA
188 DSPQLATLADEVSAS
189 DSPQLATLADEVSASLAKQGL
190 DSPQLA
191 DSPQLATL
192 DSPQLATLADEV
193 DSPQLATLADEVSASLAKQGL
194 DSPQLATLADEV
195 DSPQLATLADEV
196 DSPQLATLADEV
197 DSPQLATLADEVSASLAKQGL
198     LATLADEVSASLAKQGL
199      ATLADEVSASLAKQGL
200       TLADEVSASLAKQGL
201        LADEVSASLAKQGL
202         ADEVSASLAK
203         ADEVSASLAKQG
204         ADEVSASLAKQGL
205             SASLAKQGL
206              ASLAKQGL
```

Supporting data 5. Peptides identified in tau CN fibrils digested by HTRA1

Tau CN (β-sheets highlighted in yellow, N-and C-terminal swaps highlighted in grey)

MAEPRQEFEVMEDHAGTYGLGDRKDQGGYTMHQDQEGDTDAEIVYKSPVVSGDTSPRHLSNVSST
GSIDMVDSPQLATLADEVSASLAKQGLEGTTAEEAGIGDTPSLEDEAAGHVTQARMVSKSKDGTG
SDDKKAKGADGKTKIATPRGAAPPGQKGQANATRIPAKTPPAPKTPPSSGEPPKSGDRSGYSSPG
SPGTPGSRSRTPSLPTPPTREPKKVAVVRTPPKSPSSAKSRLQTAPVPMPDLKNVKSKIGSTENL
KHQPGGGKVQIINKKLDLSNVQSKCGSKDNIKHVPGGGSVQIVYKPVDLSKVTSKCGSLGNIHHK
PGGGQVEVKSEKLDFKDRVQSKIGSLDNITHVPGGGNKKIETHKLTFRENAKAKTDHGAGLKESP
LQTPTEDGSEEPGSETSDAKSTPTAEDVTAPLVDEGAPGKQAAAQPHTEIP

Tau CN fibrils 0 sec

```
   1 MAEPRQEFEVMEDHAGTYGLGDRKDQGGYTMHQDQEGDTDAEIVYKSPVVSGDTSPRHLS

   1 NVSSTGSIDMVDSPQLATLADEVSASLAKQGLEGTTAEEAGIGDTPSLEDEAAGHVTQAR
   2                        SASLAKQGL
   3                        SASLAKQGLEGTTAEEA
   4                        SASLAKQGLEGTTAEEAGIGDTPSLEDEAAGHVTQA

   1 MVSKSKDGTGSDDKKAKGADGKTKIATPRGAAPPGQKGQANATRIPAKTPPAPKTPPSSG

   1 EPPKSGDRSGYSSPGSPGTPGSRSRTPSLPTPPTREPKKVAVVRTPPKSPSSAKSRLQTA
   5                                                         LQTA

   1 PVPMPDLKNVKSKIGSTENLKHQPGGGKVQIINKKLDLSNVQSKCGSKDNIKHVPGGGSV
   5 PVPMPDLKNVKSKIG

   1 QIVYKPVDLSKVTSKCGSLGNIHHKPGGGQVEVKSEKLDFKDRVQSKIGSLDNITHVPGG

   1 GNKKIETHKLTFRENAKAKTDHGAGLKESPLQTPTEDGSEEPGSETSDAKSTPTAEDVTA

   1 PLVDEGAPGKQAAAQPHTEIP
```

Tau CN fibrils 15 sec

```
 1 MAEPRQEFEVMEDHAGTYGLGDRKDQGGYTMHQDQEGDTDAEIVYKSPVVSGDTSPRHLS
 2  AEPRQEFEV
 3                                            VYKSPVVSGDTSPRH
 4                                            VYKSPVVSGDTSPRHL
 5                                            VYKSPVVSGDTSPRHLS
 6                                            VYKSPVVSGDTSPRHLS
 7                                                            S
 8                                                            S
 9                                                            S

 1 NVSSTGSIDMVDSPQLATLADEVSASLAKQGLEGTTAEEAGIGDTPSLEDEAAGHVTQAR
 6 NV
 7 NVSSTGSIDMVDSPQLATL
 8 NVSSTGSIDMVDSPQLATLADEV
 9 NVSSTGSIDMVDSPQLATLADEVSASLAKQGL
10   SSTGSIDMVDSPQLATL
11   SSTGSIDMVDSPQLATLADEV
12                    ADEVSASLAKQGL
13                        SASLAKQGL
14                        SASLAKQGLEGTTAEEA
15                        SASLAKQGLEGTTAEEAGIGDTPSLEDEAAGHVTQA

 1 MVSKSKDGTGSDDKKAKGADGKTKIATPRGAAPPGQKGQANATRIPAKTPPAPKTPPSSG

 1 EPPKSGDRSGYSSPGSPGTPGSRSRTPSLPTPPTREPKKVAVVRTPPKSPSSAKSRLQTA
16                                         AVVRTPPKSPSSAKSRLQTA
17                                                    SAKSRLQTA
18                                                         LQTA

 1 PVPMPDLKNVKSKIGSTENLKHQPGGGKVQIINKKLDLSNVQSKCGSKDNIKHVPGGGSV
16 PVPMPDLKNV
17 PVPMPDLKNV
18 PVPMPDLKNVKSKIG
19                               IINKKLDLSNV
20                                              GSKDNIKHVPGGGSV

 1 QIVYKPVDLSKVTSKCGSLGNIHHKPGGGQVEVKSEKLDFKDRVQSKIGSLDNITHVPGG
20 QI
21  IVYKPVDLSKV
22   VYKPVDLSKV
23   VYKPVDLSKVTS
24                                       DFKDRVQSKIGSLDNI
25                                                       THVPGG

 1 GNKKIETHKLTFRENAKAKTDHGAGLKESPLQTPTEDGSEEPGSETSDAKSTPTAEDVTA
25 GNKKIETH
26           TFRENAKAKTD

 1 PLVDEGAPGKQAAAQPHTEIP
```

Tau CN fibrils 1 min

```
 1 MAEPRQEFEVMEDHAGTYGLGDRKDQGGYTMHQDQEGDTDAEIVYKSPVVSGDTSPRHLS
 2  AEPRQEFEV
 3                                            VYKSPVVSGDTSPR
 4                                            VYKSPVVSGDTSPRH
 5                                            VYKSPVVSGDTSPRHL
 6                                            VYKSPVVSGDTSPRHLS
 7                                            VYKSPVVSGDTSPRHLS
 8                                            VYKSPVVSGDTSPRHLS
 9                                            VYKSPVVSGDTSPRHLS
10                                             YKSPVVSGDTSPRHL
11                                                  VSGDTSPRHL
12                                                   SGDTSPRHL
13                                                            S
14                                                            S
15                                                            S

 1 NVSSTGSIDMVDSPQLATLADEVSASLAKQGLEGTTAEEAGIGDTPSLEDEAAGHVTQAR
 7 N
 8 NV
 9 NVS
13 NVSSTGSIDMVDSPQL
14 NVSSTGSIDMVDSPQLATL
15 NVSSTGSIDMVDSPQLATLADEVSASLAKQGL
16   SSTGSIDMVDSPQLATL
17   SSTGSIDMVDSPQLATLADEVSASLAKQGL
18            DSPQLATLADEVSASLAKQGL
19                  TLADEVSASLAKQGL
20                   LADEVSASLAKQGL
21                    ADEVSASLAKQ
22                    ADEVSASLAKQGL
23                        SASLAKQGL
24                        SASLAKQGLEGTTAEEAGIGDTPSLEDEAAGHVTQA

 1 MVSKSKDGTGSDDKKAKGADGKTKIATPRGAAPPGQKGQANATRIPAKTPPAPKTPPSSG

 1 EPPKSGDRSGYSSPGSPGTPGSRSRTPSLPTPPTREPKKVAVVRTPPKSPSSAKSRLQTA
25                                         AVVRTPPKSPSSAKSRLQTA
26                                          VVRTPPKSPSSAKSRLQTA
27                                            RTPPKSPSSAKSRLQTA
28                                                    SAKSRLQTA
29                                                       SRLQTA
30                                                         LQTA
31                                                         LQTA
32                                                           TA

 1 PVPMPDLKNVKSKIGSTENLKHQPGGGKVQIINKKLDLSNVQSKCGSKDNIKHVPGGGSV
25 PVPMPDLKNV
26 PVPMPDLKNV
27 PVPMPDLKNV
28 PVPMPDLKNV
29 PVPMPDLKNV
30 PVPMPDLKNV
31 PVPMPDLKNVKSKIG
32 PVPMPDLKNVKSKIGSTENL
33           KSKIGSTENL
34            SKIGSTENL
35                     KHQPGGGKVQI
36                               IINKKLDLSNV
37                                INKKLDLSNV
38                                 NKKLDLSNV
39                                              GSKDNIKHVPGGGSV
```

```
40                                               SKDNIKHVPGGGSV

 1 QIVYKPVDLSKVTSKCGSLGNIHHKPGGGQVEVKSEKLDFKDRVQSKIGSLDNITHVPGG
39 QI
40 QI
41  IVYKPVDLSKV
42   VYKPVDL
43   VYKPVDLSKV
44   VYKPVDLSKVT
45   VYKPVDLSKVTS
46   VYKPVDLSKVTSK
47                 GSLGNIHHKPGGGQ
48                 GSLGNIHHKPGGGQVEV
49                                       DFKDRVQSKIGSL
50                                       DFKDRVQSKIGSLDNI
51                                             QSKIGSLDNI
52                                                    DNITHVPGG
53                                                       THVPGG
54                                                       THVPGG
55                                                       THVPGG
56                                                       THVPGG
57                                                       THVPGG
58                                                         VPGG
59                                                          PGG
60                                                           GG
61                                                            G

 1 GNKKIETHKLTFRENAKAKTDHGAGLKESPLQTPTEDGSEEPGSETSDAKSTPTAEDVTA
52 GNKKIE
53 GNKKIE
54 GNKKIETH
55 GNKKIETHK
56 GNKKIETHKL
57 GNKKIETHKLT
58 GNKKIETHKL
59 GNKKIETHKL
60 GNKKIETHKL
61 GNKKIETHKL
62 GNKKIETHKL
63           TFRENAKAKTD
64           TFRENAKAKTDHGA

 1 PLVDEGAPGKQAAAQPHTEIP
```

Tau CN fibrils 2 min

```
  1 MAEPRQEFEVMEDHAGTYGLGDRKDQGGYTMHQDQEGDTDAEIVYKSPVVSGDTSPRHLS
  2  AEPRQEFEV
  3                                            VYKSPVVS
  4                                            VYKSPVVSGDT
  5                                            VYKSPVVSGDTSPR
  6                                            VYKSPVVSGDTSPRH
  7                                            VYKSPVVSGDTSPRHL
  8                                            VYKSPVVSGDTSPRHLS
  9                                                  VSGDTSPRHL
 10                                                  VSGDTSPRHLS
 11                                                  VSGDTSPRHLS
 12                                                   SGDTSPRHL
 13                                                            S
 14                                                            S
 15                                                            S
 16                                                            S
 17                                                            S
 18                                                            S
 19                                                            S
 20                                                            S
 21                                                            S
 22                                                            S

  1 NVSSTGSIDMVDSPQLATLADEVSASLAKQGLEGTTAEEAGIGDTPSLEDEAAGHVTQAR
  8 NV
 10 NV
 11 NVSSTGSIDMVDSPQLATL
 13 NVSSTGSIDMV
 14 NVSSTGSIDMVDSPQL
 15 NVSSTGSIDMVDSPQLA
 16 NVSSTGSIDMVDSPQLAT
 17 NVSSTGSIDMVDSPQLATL
 18 NVSSTGSIDMVDSPQLATLA
 19 NVSSTGSIDMVDSPQLATLADEV
 20 NVSSTGSIDMVDSPQLATLADEVSAS
 21 NVSSTGSIDMVDSPQLATLADEVSASLAKQ
 22 NVSSTGSIDMVDSPQLATLADEVSASLAKQGL
 23   SSTGSIDMVDSPQLA
 24   SSTGSIDMVDSPQLATL
 25   SSTGSIDMVDSPQLATLADEV
 26   SSTGSIDMVDSPQLATLADEVSAS
 27   SSTGSIDMVDSPQLATLADEVSASLAKQGL
 28      GSIDMVDSPQLA
 29      GSIDMVDSPQLATL
 30      GSIDMVDSPQLATLADEV
 31            DSPQLATLADEVSASLAKQGL
 32                LATLADEVSASLAKQGL
 33                 ATLADEVSASLAKQGL
 34                  TLADEVSASLAKQ
 35                  TLADEVSASLAKQGL
 36                   LADEVSASLAKQGL
 37                    ADEVSASLA
 38                    ADEVSASLAKQ
 39                    ADEVSASLAKQGL
 40                     DEVSASLAKQGL
 41                      EVSASLAKQGL
 42                        SASLAKQGL
 43                        SASLAKQGLEGT
 44                        SASLAKQGLEGTTAEEA
 45                          SLAKQGL
```

```
 1 MVSKSKDGTGSDDKKAKGADGKTKIATPRGAAPPGQKGQANATRIPAKTPPAPKTPPSSG

 1 EPPKSGDRSGYSSPGSPGTPGSRSRTPSLPTPPTREPKKVAVVRTPPKSPSSAKSRLQTA
46                                         AVVRTPPKSPSSAKSRLQTA
47                                                    SAKSRLQTA
48                                                       SRLQTA
49                                                        RLQTA
50                                                         LQTA
51                                                           TA
52                                                           TA
53                                                            A

 1 PVPMPDLKNVKSKIGSTENLKHQPGGGKVQIINKKLDLSNVQSKCGSKDNIKHVPGGGSV
46 PVPMPDLKNV
47 PVPMPDLKNV
48 PVPMPDLKNV
49 PVPMPDLKNV
50 PVPMPDLKNV
51 PVPMPDLKNV
52 PVPMPDLKNVKSKIGSTENL
53 PVPMPDLKNV
54 PVPMPDLKNV
55  VPMPDLKNV
56   PMPDLKNV
57           KSKIGSTENL
58            SKIGSTENL
59                     KHQPGGGKVQI
60                               IINKKLDLSNV
61                                INKKLDLSNV
62                                   KLDLSNV
63                                                    KHVPGGGSV

 1 QIVYKPVDLSKVTSKCGSLGNIHHKPGGGQVEVKSEKLDFKDRVQSKIGSLDNITHVPGG
63 QI
64  IVYKPVDLSKV
65   VYKPVDL
66   VYKPVDLS
67   VYKPVDLSKV
68   VYKPVDLSKVTS
69                                       DFKDRVQSKIGSLDNI
70                                             QSKIGSLDNI
71                                              SKIGSLDNI
72                                                          PGG
73                                                           GG
74                                                            G

 1 GNKKIETHKLTFRENAKAKTDHGAGLKESPLQTPTEDGSEEPGSETSDAKSTPTAEDVTA
72 GNKKIETHKL
73 GNKKIETHKL
74 GNKKIETHKL

 1 PLVDEGAPGKQAAAQPHTEIP
```

Tau CN fibrils 5 min

```
 1 MAEPRQEFEVMEDHAGTYGLGDRKDQGGYTMHQDQEGDTDAEIVYKSPVVSGDTSPRHLS
 2                                            VYKSPVVSGDT
 3                                            VYKSPVVSGDTSPR
 4                                            VYKSPVVSGDTSPRH
 5                                            VYKSPVVSGDTSPRHL
 6                                            VYKSPVVSGDTSPRHLS
 7                                            VYKSPVVSGDTSPRHLS
 8                                            VYKSPVVSGDTSPRHLS
 9                                            VYKSPVVSGDTSPRHLS
10                                            VYKSPVVSGDTSPRHLS
11                                             YKSPVVSGDTSPRHL
12                                             YKSPVVSGDTSPRHLS
13                                                  VSGDTSPRHL
14                                                   SGDTSPRHL
15                                                            S
16                                                            S
17                                                            S
18                                                            S
19                                                            S
20                                                            S
21                                                            S

 1 NVSSTGSIDMVDSPQLATLADEVSASLAKQGLEGTTAEEAGIGDTPSLEDEAAGHVTQAR
 7 N
 8 NV
 9 NVS
10 NVSST
12 NV
15 NVSSTGSIDMV
16 NVSSTGSIDMVDSPQL
17 NVSSTGSIDMVDSPQLA
18 NVSSTGSIDMVDSPQLATL
19 NVSSTGSIDMVDSPQLATLA
20 NVSSTGSIDMVDSPQLATLADEV
21 NVSSTGSIDMVDSPQLATLADEVSASLAKQGL
22   SSTGSIDMVDSPQLA
23   SSTGSIDMVDSPQLATL
24   SSTGSIDMVDSPQLATLADEV
25   SSTGSIDMVDSPQLATLADEVSASLAKQGL
26            DSPQLATLADEVSASLAKQGL
27                LATLADEVSASLAKQGL
28                 ATLADEVSASLAKQGL
29                  TLADEVSASLAKQGL
30                   LADEVSASLAKQGL
31                    ADEVSASLA
32                    ADEVSASLAKQ
33                    ADEVSASLAKQGL
34                        SASLAKQGL
35                        SASLAKQGLEGT
36                        SASLAKQGLEGTTAEEAGIGDTPSLEDEAAGHVTQA

 1 MVSKSKDGTGSDDKKAKGADGKTKIATPRGAAPPGQKGQANATRIPAKTPPAPKTPPSSG

 1 EPPKSGDRSGYSSPGSPGTPGSRSRTPSLPTPPTREPKKVAVVRTPPKSPSSAKSRLQTA
37                                         AVVRTPPKSPSSAKS
38                                         AVVRTPPKSPSSAKSRLQTA
39                                          VVRTPPKSPSSAKSRLQTA
40                                                    SAKSRLQTA
41                                                       SRLQTA
42                                                         LQTA
43                                                         LQTA
```

```
44                                                            TA
45                                                            TA
46                                                             A

 1 PVPMPDLKNVKSKIGSTENLKHQPGGGKVQIINKKLDLSNVQSKCGSKDNIKHVPGGGSV
38 PVPMPDLKNV
39 PVPMPDLKNV
40 PVPMPDLKNV
41 PVPMPDLKNV
42 PVPMPDLKNV
43 PVPMPDLKNVKSKIG
44 PVPMPDLKNVKSKIGST
45 PVPMPDLKNVKSKIGSTENL
46 PVPMPDLKNV
47           KSKIGSTENL
48            SKIGSTENL
49                     KHQPGGGKVQI
50                               IINKKLDLSNV
51                                INKKLDLSNV
52                                 NKKLDLSNV
53                                              GSKDNIKHVPGGGSV
54                                               SKDNIKHVPGGGSV
55                                                    KHVPGGGSV

 1 QIVYKPVDLSKVTSKCGSLGNIHHKPGGGQVEVKSEKLDFKDRVQSKIGSLDNITHVPGG
53 QI
54 QI
55 QI
56  IVYKPVDLSKV
57   VYKPVDL
58   VYKPVDLSKV
59   VYKPVDLSKVT
60   VYKPVDLSKVTS
61   VYKPVDLSKVTSK
62    YKPVDLSKV
63                 GSLGNIHHKPGGGQVEV
64                 GSLGNIHHKPGGGQVEVKSEKL
65                                        DFKDRVQS
66                                        DFKDRVQSKIGSL
67                                        DFKDRVQSKIGSLDNI
68                                              QSKIGSLDNI
69                                                     DNITHVPGG
70                                                        THVPGG
71                                                        THVPGG
72                                                        THVPGG
73                                                        THVPGG
74                                                        THVPGG
75                                                        THVPGG
76                                                          VPGG
77                                                           PGG
78                                                            GG
79                                                             G

 1 GNKKIETHKLTFRENAKAKTDHGAGLKESPLQTPTEDGSEEPGSETSDAKSTPTAEDVTA
69 GNKKIETHKL
70 GNKKIE
71 GNKKIET
72 GNKKIETH
73 GNKKIETHK
74 GNKKIETHKL
75 GNKKIETHKLT
76 GNKKIETHKL
77 GNKKIETHKL
```

```
78 GNKKIETHKL
79 GNKKIETHKL
80 GNKKIETHKL
81           TFRENAKAKTD
82           TFRENAKAKTDHGA

 1 PLVDEGAPGKQAAAQPHTEIP
```

Tau CN fibrils 10 min

```
 1 MAEPRQEFEVMEDHAGTYGLGDRKDQGGYTMHQDQEGDTDAEIVYKSPVVSGDTSPRHLS
 2                                            VYKSPVVS
 3                                            VYKSPVVSGDTSPR
 4                                            VYKSPVVSGDTSPRH
 5                                            VYKSPVVSGDTSPRHL
 6                                            VYKSPVVSGDTSPRHLS
 7                                            VYKSPVVSGDTSPRHLS
 8                                            VYKSPVVSGDTSPRHLS
 9                                            VYKSPVVSGDTSPRHLS
10                                            VYKSPVVSGDTSPRHLS
11                                            VYKSPVVSGDTSPRHLS
12                                            VYKSPVVSGDTSPRHLS
13                                             YKSPVVSGDTSPRHL
14                                             YKSPVVSGDTSPRHLS
15                                               SPVVSGDTSPRHL
16                                                  VSGDTSPRHL
17                                                  VSGDTSPRHLS
18                                                  VSGDTSPRHLS
19                                                   SGDTSPRHL
20                                                   SGDTSPRHLS
21                                                            S
22                                                            S
23                                                            S
24                                                            S
25                                                            S
26                                                            S
27                                                            S
28                                                            S
29                                                            S

 1 NVSSTGSIDMVDSPQLATLADEVSASLAKQGLEGTTAEEAGIGDTPSLEDEAAGHVTQAR
 7 N
 8 NV
 9 NVS
10 NVSST
11 NVSSTG
12 NVSSTGSIDMVDSPQLA
14 NV
17 NV
18 NVSSTGSIDMVDSPQLATL
20 NV
21 NVSSTGSIDMV
22 NVSSTGSIDMVDSPQL
23 NVSSTGSIDMVDSPQLA
24 NVSSTGSIDMVDSPQLAT
25 NVSSTGSIDMVDSPQLATL
26 NVSSTGSIDMVDSPQLATLA
27 NVSSTGSIDMVDSPQLATLADEV
28 NVSSTGSIDMVDSPQLATLADEVSAS
29 NVSSTGSIDMVDSPQLATLADEVSASLAKQGL
30   SSTGSIDMVDSPQLA
31   SSTGSIDMVDSPQLATL
32   SSTGSIDMVDSPQLATLADEV
33   SSTGSIDMVDSPQLATLADEVSAS
34   SSTGSIDMVDSPQLATLADEVSASLAKQGL
35      GSIDMVDSPQLATL
36            DSPQLATLADEVSASLAKQGL
37                LATLADEVSASLAKQGL
38                 ATLADEVSASLAKQGL
39                  TLADEVSASLAKQ
40                  TLADEVSASLAKQGL
```

```
41                   LADEVSASLAKQGL
42                    ADEVSASLA
43                    ADEVSASLAKQ
44                    ADEVSASLAKQGL
45                     DEVSASLAKQGL
46                      EVSASLAKQGL
47                        SASLAKQGL
48                        SASLAKQGLEGT
49                        SASLAKQGLEGTTAEEA
50                          SLAKQGL

 1 MVSKSKDGTGSDDKKAKGADGKTKIATPRGAAPPGQKGQANATRIPAKTPPAPKTPPSSG

 1 EPPKSGDRSGYSSPGSPGTPGSRSRTPSLPTPPTREPKKVAVVRTPPKSPSSAKSRLQTA
51                      SRSRTPSLPTPPTREPKKV
52                                         AVVRTPPKSPSSAKS
53                                         AVVRTPPKSPSSAKSRLQ
54                                         AVVRTPPKSPSSAKSRLQTA
55                                          VVRTPPKSPSSAKSRLQTA
56                                           VRTPPKSPSSAKSRLQTA
57                                            RTPPKSPSSAKSRLQTA
58                                                    SAKSRLQTA
59                                                    SAKSRLQTA
60                                                       SRLQTA
61                                                        RLQTA
62                                                         LQTA
63                                                         LQTA
64                                                           TA
65                                                           TA
66                                                            A

 1 PVPMPDLKNVKSKIGSTENLKHQPGGGKVQIINKKLDLSNVQSKCGSKDNIKHVPGGGSV
54 PVPMPDLKNV
55 PVPMPDLKNV
56 PVPMPDLKNV
57 PVPMPDLKNV
58 PVPMPDLKNV
59 PVPMPDLKNVKSKIGSTENL
60 PVPMPDLKNV
61 PVPMPDLKNV
62 PVPMPDLKNV
63 PVPMPDLKNVKSKIG
64 PVPMPDLKNV
65 PVPMPDLKNVKSKIGSTENL
66 PVPMPDLKNV
67 PVPMPDLKNV
68           KSKIGSTENL
69           KSKIGSTENLKHQPGGGKVQ
70            SKIGSTENL
71                     KHQPGGGKVQI
72                     KHQPGGGKVQIINKKLDLSNV
73                               IINKKLDLSNV
74                               IINKKLDLSNVQS
75                                INKKLDLSNV
76                                 NKKLDLSNV
77                                  KKLDLSNV
78                                   KLDLSNV
79                                              GSKDNIKHVPGGGSV
80                                               SKDNIKHVPGGGSV
81                                                    KHVPGGGSV

 1 QIVYKPVDLSKVTSKCGSLGNIHHKPGGGQVEVKSEKLDFKDRVQSKIGSLDNITHVPGG
79 QI
```

```
 80 QI
 81 QI
 82  IVYKPVDLSKV
 83  IVYKPVDLSKVTS
 84   VYKPVDL
 85   VYKPVDLS
 86   VYKPVDLSKV
 87   VYKPVDLSKVT
 88   VYKPVDLSKVTS
 89   VYKPVDLSKVTSK
 90   VYKPVDLSKVTSKC
 91    YKPVDLSKV
 92                 GSLGNIHHKPGGGQ
 93                 GSLGNIHHKPGGGQVEV
 94                 GSLGNIHHKPGGGQVEVKSEKL
 95                  SLGNIHHKPGGGQVE
 96                                       DFKDRVQ
 97                                       DFKDRVQS
 98                                       DFKDRVQSK
 99                                       DFKDRVQSKIG
100                                       DFKDRVQSKIGSL
101                                       DFKDRVQSKIGSLDNI
102                                       DFKDRVQSKIGSLDNIT
103                                             QSKIGSLDNI
104                                             QSKIGSLDNITHVPGG
105                                                    DNITHVPGG
106                                                    DNITHVPGG
107                                                       THVPGG
108                                                       THVPGG
109                                                       THVPGG
110                                                       THVPGG
111                                                       THVPGG
112                                                       THVPGG
113                                                         VPGG
114                                                          PGG
115                                                          PGG
116                                                           GG
117                                                           GG
118                                                            G

  1 GNKKIETHKLTFRENAKAKTDHGAGLKESPLQTPTEDGSEEPGSETSDAKSTPTAEDVTA
104 GNKKIETHKL
105 GNKKIE
106 GNKKIETHKL
107 GNKKIE
108 GNKKIET
109 GNKKIETH
110 GNKKIETHK
111 GNKKIETHKL
112 GNKKIETHKLT
113 GNKKIETHKL
114 GNKKIE
115 GNKKIETHKL
116 GNKKIE
117 GNKKIETHKL
118 GNKKIETHKL
119 GNKKIETHKL
120           TFRENAKAKTD
121           TFRENAKAKTDHGA
122           TFRENAKAKTDHGAGLK
123           TFRENAKAKTDHGAGLKESPLQTPTEDGSEEPGSE

  1 PLVDEGAPGKQAAAQPHTEIP
```

Tau CN fibrils 120 min

```
  1 MAEPRQEFEVMEDHAGTYGLGDRKDQGGYTMHQDQEGDTDAEIVYKSPVVSGDTSPRHLS
  2  AEPRQEFEVM
  3                                            VYKSPVVS
  4                                            VYKSPVVSGDT
  5                                            VYKSPVVSGDTSPR
  6                                            VYKSPVVSGDTSPRH
  7                                            VYKSPVVSGDTSPRHL
  8                                            VYKSPVVSGDTSPRHLS
  9                                            VYKSPVVSGDTSPRHLS
 10                                            VYKSPVVSGDTSPRHLS
 11                                            VYKSPVVSGDTSPRHLS
 12                                            VYKSPVVSGDTSPRHLS
 13                                            VYKSPVVSGDTSPRHLS
 14                                            VYKSPVVSGDTSPRHLS
 15                                             YKSPVVSGDTSPRHL
 16                                             YKSPVVSGDTSPRHLS
 17                                               SPVVSGDTSPRHL
 18                                                  VSGDTSPRHL
 19                                                  VSGDTSPRHLS
 20                                                  VSGDTSPRHLS
 21                                                   SGDTSPRHL
 22                                                   SGDTSPRHLS
 23                                                          HLS
 24                                                          HLS
 25                                                            S
 26                                                            S
 27                                                            S
 28                                                            S
 29                                                            S
 30                                                            S
 31                                                            S
 32                                                            S
 33                                                            S

  1 NVSSTGSIDMVDSPQLATLADEVSASLAKQGLEGTTAEEAGIGDTPSLEDEAAGHVTQAR
  9 N
 10 NV
 11 NVS
 12 NVSST
 13 NVSSTG
 14 NVSSTGSIDMVDSPQLA
 16 NV
 19 NV
 20 NVSSTGSIDMVDSPQLATL
 22 NV
 23 NVSSTGSIDMVDSPQLATL
 24 NVSSTGSIDMVDSPQLATLADEV
 25 NVSSTGSIDMV
 26 NVSSTGSIDMVDSPQL
 27 NVSSTGSIDMVDSPQLA
 28 NVSSTGSIDMVDSPQLAT
 29 NVSSTGSIDMVDSPQLATL
 30 NVSSTGSIDMVDSPQLATLA
 31 NVSSTGSIDMVDSPQLATLADEV
 32 NVSSTGSIDMVDSPQLATLADEVSAS
 33 NVSSTGSIDMVDSPQLATLADEVSASLAKQGL
 34   SSTGSIDMVDSPQLA
 35   SSTGSIDMVDSPQLATL
 36   SSTGSIDMVDSPQLATLADEV
 37   SSTGSIDMVDSPQLATLADEVSAS
 38   SSTGSIDMVDSPQLATLADEVSASLAKQGL
```

```
39      GSIDMVDSPQLA
40      GSIDMVDSPQLATL
41       SIDMVDSPQLATL
42            DSPQLATLADEV
43                  TLADEVSASLAKQGL
44                   LADEVSASLAKQGL
45                    ADEVSASLA
46                    ADEVSASLAKQ
47                    ADEVSASLAKQGL
48                        SASLAKQGL
49                        SASLAKQGLEGT
50                        SASLAKQGLEGTTAEEA
51                                         GIGDTPSLEDEAAGHVTQA
52                                         GIGDTPSLEDEAAGHVTQAR
53                                             TPSLEDEAAGHVTQA

 1 MVSKSKDGTGSDDKKAKGADGKTKIATPRGAAPPGQKGQANATRIPAKTPPAPKTPPSSG
52 MV

 1 EPPKSGDRSGYSSPGSPGTPGSRSRTPSLPTPPTREPKKVAVVRTPPKSPSSAKSRLQTA
54                      SRSRTPSLPTPPTREPKKV
55                                         AVVRTPPKSPS
56                                         AVVRTPPKSPSSA
57                                         AVVRTPPKSPSSAKS
58                                         AVVRTPPKSPSSAKSRL
59                                         AVVRTPPKSPSSAKSRLQ
60                                         AVVRTPPKSPSSAKSRLQT
61                                         AVVRTPPKSPSSAKSRLQTA
62                                         AVVRTPPKSPSSAKSRLQTA
63                                          VVRTPPKSPS
64                                          VVRTPPKSPSSAKSRLQTA
65                                           VRTPPKSPS
66                                           VRTPPKSPSSAKSRLQTA
67                                            RTPPKSPSSAKS
68                                            RTPPKSPSSAKSRLQ
69                                            RTPPKSPSSAKSRLQT
70                                            RTPPKSPSSAKSRLQTA
71                                             TPPKSPSSAKSRLQTA
72                                               PKSPSSAKSRLQ
73                                                 SPSSAKSRLQTA
74                                                   SSAKSRLQTA
75                                                    SAKSRLQTA
76                                                    SAKSRLQTA
77                                                      KSRLQTA
78                                                       SRLQTA
79                                                        RLQTA
80                                                         LQTA
81                                                         LQTA
82                                                          QTA
83                                                           TA
84                                                           TA
85                                                           TA
86                                                            A

 1 PVPMPDLKNVKSKIGSTENLKHQPGGGKVQIINKKLDLSNVQSKCGSKDNIKHVPGGGSV
61 PVPMPDL
62 PVPMPDLKNV
64 PVPMPDLKNV
66 PVPMPDLKNV
70 PVPMPDLKNV
71 PVPMPDLKNV
73 PVPMPDLKNV
74 PVPMPDLKNV
```

```
 75 PVPMPDLKNV
 76 PVPMPDLKNVKSKIGSTENL
 77 PVPMPDLKNV
 78 PVPMPDLKNV
 79 PVPMPDLKNV
 80 PVPMPDLKNV
 81 PVPMPDLKNVKSKIGSTENL
 82 PVPMPDLKNV
 83 PVPMPDLKNV
 84 PVPMPDLKNVKSKIGST
 85 PVPMPDLKNVKSKIGSTENL
 86 PVPMPDLKNV
 87 PVPMPDLKNV
 88  VPMPDLKNV
 89   PMPDLKNV
 90           KSKIGSTENL
 91           KSKIGSTENLKHQPGGGKVQ
 92           KSKIGSTENLKHQPGGGKVQI
 93            SKIGSTENL
 94            SKIGSTENLK
 95                     KHQPGGGKVQI
 96                     KHQPGGGKVQII
 97                     KHQPGGGKVQIINK
 98                     KHQPGGGKVQIINKKLDL
 99                     KHQPGGGKVQIINKKLDLSNV
100                              QIINKKLDLSNV
101                               IINKKLDLSNV
102                               IINKKLDLSNVQS
103                                INKKLDLSNV
104                                INKKLDLSNVQS
105                                 NKKLDLSNV
106                                  KKLDLSNV
107                                   KLDLSNV
108                                              GSKDNIKHVPGGGSV
109                                               SKDNIKHVPGGGSV
110                                                    KHVPGGGSV

  1 QIVYKPVDLSKVTSKCGSLGNIHHKPGGGQVEVKSEKLDFKDRVQSKIGSLDNITHVPGG
108 QI
109 QI
110 QI
111  IVYKPVDLSKV
112   VYKPVDL
113   VYKPVDLSKV
114   VYKPVDLSKVT
115   VYKPVDLSKVTS
116   VYKPVDLSKVTSK
117   VYKPVDLSKVTSKC
118    YKPVDLSKV
119     KPVDLSKV
120                 GSLGNIHHKPGGGQ
121                 GSLGNIHHKPGGGQVEV
122                 GSLGNIHHKPGGGQVEVKSEKL
123                   LGNIHHKPGGGQVEVKSEKL
124                    GNIHHKPGGGQVEVKSEKL
125                                      LDFKDRVQSK
126                                       DFKDRVQ
127                                       DFKDRVQS
128                                       DFKDRVQSK
129                                       DFKDRVQSKIGSL
130                                       DFKDRVQSKIGSLD
131                                       DFKDRVQSKIGSLDNI
132                                       DFKDRVQSKIGSLDNIT
```

```
133                                       DFKDRVQSKIGSLDNITHV
134                                            VQSKIGSLDNI
135                                             QSKIGSLDNI
136                                             QSKIGSLDNIT
137                                             QSKIGSLDNITHVPGG
138                                              SKIGSLDNI
139                                                    DNITHVPGG
140                                                    DNITHVPGG
141                                                       THVPGG
142                                                       THVPGG
143                                                       THVPGG
144                                                       THVPGG
145                                                       THVPGG
146                                                       THVPGG
147                                                         VPGG
148                                                          PGG
149                                                          PGG
150                                                           GG
151                                                           GG
152                                                            G

  1 GNKKIETHKLTFRENAKAKTDHGAGLKESPLQTPTEDGSEEPGSETSDAKSTPTAEDVTA
137 GNKKIETHKL
139 GNKKIE
140 GNKKIETHKL
141 GNKKIE
142 GNKKIET
143 GNKKIETH
144 GNKKIETHK
145 GNKKIETHKL
146 GNKKIETHKLT
147 GNKKIETHKL
148 GNKKIE
149 GNKKIETHKL
150 GNKKIE
151 GNKKIETHKL
152 GNKKIETHKL
153 GNKKIETHKL
154           TFRENAKAKTD
155           TFRENAKAKTDHGA
156           TFRENAKAKTDHGAGLK

  1 PLVDEGAPGKQAAAQPHTEIP
157            AAAQPHTEIP
```

Supporting data 6. Mass spectrometry information

Overview

ACE project ID	Title
ACE_0711-02	Time-resolved analysis of proteolytic cleavage sites in Tau (10 µM) by HTRA1 (2 µM) (0 s, 15 s, 1 m, 2 m, 5 m, 10 m, 120 m) in NaPi
ACE_0711-04	Time-resolved analysis of proteolytic cleavage sites in fibrillar Tau (10 µM) by HTRA1 (2 µM) (0 s, 15 s, 1 m, 2 m, 5 m, 10 m, 120 m) in HEPES
ACE_0809-03	Time-resolved analysis of proteolytic cleavage sites in fibrillar Tau_CN by HTRA1 (t= 0; 15 s; 1 m; 2 m; 5 m; 10 m; 120 m)

ACE_0711-02

File legend

ACE ID	Alternate ID	Organ/ cell line	Treatment/ experimental setup
ACE_0711-02_BH01	MS8_H_0	Human (overexpressed in *E. coli*)	2 µM HTRA1 Control, 0 sec, supernatant after acetone precipitation
ACE_0711-02_BH02	MS8_H_0	dito	2 µM HTRA1 Control, 0 sec, supernatant after acetone precipitation
ACE_0711-02_BH03	MS8_H_0	dito	2 µM HTRA1 Control, 0 sec, supernatant after acetone precipitation
ACE_0711-02_BH04	MS8_H_0	dito	2 µM HTRA1 Control, 0 sec, supernatant after acetone precipitation
ACE_0711-02_BH05	MS8_H_120	dito	2 µM HTRA1 Control, 120 min, supernatant after acetone precipitation
ACE_0711-02_BH06	MS8_H_120	dito	2 µM HTRA1 Control, 120 min, supernatant after acetone precipitation
ACE_0711-02_BH07	MS8_H_120	dito	2 µM HTRA1 Control, 120 min, supernatant after acetone precipitation
ACE_0711-02_BH08	MS8_H_120	dito	2 µM HTRA1 Control, 120 min, supernatant after acetone precipitation
ACE_0711-02_BH09	MS8_T_0	dito	10 µM Tau Control, 0 sec, supernatant after acetone precipitation
ACE_0711-02_BH10	MS8_T_0	dito	10 µM Tau Control, 0 sec, supernatant after acetone precipitation
ACE_0711-02_BH11	MS8_T_0	dito	10 µM Tau Control, 0 sec, supernatant after acetone precipitation
ACE_0711-02_BH12	MS8_T_0	dito	10 µM Tau Control, 0 sec, supernatant after acetone precipitation
ACE_0711-02_BH13	MS8_T_120	dito	10 µM Tau Control, 120 min, supernatant after acetone precipitation
ACE_0711-02_BH14	MS8_T_120	dito	10 µM Tau Control, 120 min, supernatant after acetone precipitation
ACE_0711-02_BH15	MS8_T_120	dito	10 µM Tau Control, 120 min, supernatant after acetone precipitation
ACE_0711-02_BH16	MS8_T_120	dito	10 µM Tau Control, 120 min, supernatant after acetone precipitation
ACE_0711-02_BH17	MS8_TH_0	dito	10 µM Tau + 2 µM HTRA1, 0 sec, supernatant after acetone precipitation
ACE_0711-02_BH18	MS8_TH_0	dito	10 µM Tau + 2 µM HTRA1, 0 sec, supernatant after acetone precipitation
ACE_0711-02_BH19	MS8_TH_0	dito	10 µM Tau + 2 µM HTRA1, 0 sec, supernatant after acetone precipitation
ACE_0711-02_BH20	MS8_TH_0	dito	10 µM Tau + 2 µM HTRA1, 0 sec, supernatant after acetone precipitation
ACE_0711-02_BH21	MS8_TH_15	dito	10 µM Tau + 2 µM HTRA1, 15 sec, supernatant after acetone precipitation
ACE_0711-02_BH22	MS8_TH_15	dito	10 µM Tau + 2 µM HTRA1, 15 sec, supernatant after acetone precipitation
ACE_0711-02_BH23	MS8_TH_15	dito	10 µM Tau + 2 µM HTRA1, 15 sec, supernatant after acetone precipitation
ACE_0711-02_BH24	MS8_TH_15	dito	10 µM Tau + 2 µM HTRA1, 15 sec, supernatant after acetone precipitation
ACE_0711-02_BH25	MS8_TH_1	dito	10 µM Tau + 2 µM HTRA1, 1 min, supernatant after acetone precipitation
ACE_0711-02_BH26	MS8_TH_1	dito	10 µM Tau + 2 µM HTRA1, 1 min, supernatant after acetone precipitation
ACE_0711-02_BH27	MS8_TH_1	dito	10 µM Tau + 2 µM HTRA1, 1 min, supernatant after acetone precipitation
ACE_0711-02_BH28	MS8_TH_1	dito	10 µM Tau + 2 µM HTRA1, 1 min, supernatant after acetone precipitation
ACE_0711-02_BH29	MS8_TH_5	dito	10 µM Tau + 2 µM HTRA1, 5 min, supernatant after acetone precipitation
ACE_0711-02_BH30	MS8_TH_5	dito	10 µM Tau + 2 µM HTRA1, 5 min, supernatant after acetone precipitation
ACE_0711-02_BH31	MS8_TH_5	dito	10 µM Tau + 2 µM HTRA1, 5 min, supernatant after acetone precipitation
ACE_0711-02_BH32	MS8_TH_5	dito	10 µM Tau + 2 µM HTRA1, 5 min, supernatant after acetone precipitation
ACE_0711-02_BH33	MS8_TH_10	dito	10 µM Tau + 2 µM HTRA1, 10 min, supernatant after acetone precipitation
ACE_0711-02_BH34	MS8_TH_10	dito	10 µM Tau + 2 µM HTRA1, 10 min, supernatant after acetone precipitation
ACE_0711-02_BH35	MS8_TH_10	dito	10 µM Tau + 2 µM HTRA1, 10 min, supernatant after acetone precipitation
ACE_0711-02_BH36	MS8_TH_10	dito	10 µM Tau + 2 µM HTRA1, 10 min, supernatant after acetone precipitation
ACE_0711-02_BH37	MS8_TH_2	dito	10 µM Tau + 2 µM HTRA1, 2 min, supernatant after acetone precipitation

ACE_0711-02_BH38	MS8_TH_2	dito	10 µM Tau + 2 µM HTRA1, 2 min, supernatant after acetone precipitation
ACE_0711-02_BH39	MS8_TH_2	dito	10 µM Tau + 2 µM HTRA1, 2 min, supernatant after acetone precipitation
ACE_0711-02_BH40	MS8_TH_2	dito	10 µM Tau + 2 µM HTRA1, 2 min, supernatant after acetone precipitation
ACE_0711-02_BH41	MS8_TH_120	dito	10 µM Tau + 2 µM HTRA1, 120 min, supernatant after acetone precipitation
ACE_0711-02_BH42	MS8_TH_120	dito	10 µM Tau + 2 µM HTRA1, 120 min, supernatant after acetone precipitation
ACE_0711-02_BH43	MS8_TH_120	dito	10 µM Tau + 2 µM HTRA1, 120 min, supernatant after acetone precipitation
ACE_0711-02_BH44	MS8_TH_120	dito	10 µM Tau + 2 µM HTRA1, 120 min, supernatant after acetone precipitation

LC_Settings

MS device	Orbitrap Elite
LC device	Evosep One
ion source	Thermo Nanospray Flex
**Analytical column**	EV1109 Analytical Column – 60 & 100 samples/day
column diameter	Length (L_C) = 8 cm; ID = 150; OD = PEEK; emitter EV-1086 Stainless steel emitter
stationary phase	Dr Maisch C18 AQ, 1.5 µm beads
particle diameter (d_p)	1.5 µm
Pore size	120 Å
Column ID	AC_EVO-006
Column oven	na
Column oven temp.	na
**solvents**	A: 0.1% FA in UPLC water<br>B: 0.1% FA in UPLC ACN
gradient	21 min gradient (60 SPD)

MS_Settings

Project	MS	general	MS1	MS2	MS2	MS3	Comments; special settings
ACE_0644	Elite	Tune v2.7.0.1112 SP2<br>Gradient: 21 min	Analyzer: FT<br>Res.: 60000<br>SR: 300 - 1500<br>AGC: 3×10^6<br>AcT: 50<br>RF: 30<br>SF: --<br>DDM: NS/15	Analyzer: IT<br>Res./ScR: - /rapid<br>SR: Auto<br>AGC: 1×10^4<br>AcT: 60<br>CS: +2 and higher<br>IsM: IT<br>IsW: 2.0<br>Frag.: CID<br>NCE: 35			classic orbitrap experiment: MS1 in Orbitrap at high resolution and data dependent MS2 in Iontrap at rapid scan rate. Dynamic exclusion enabled (exclude after n times=1; Exclusion duration (s)= 30; mass tolerance= ± 100 ppm), exclusion list size: 500

Note: **FT**= Fourier Transform (Orbitrap); **IT**= Iontrap; **Q**= Quadrupol; **Res.**= max. Resolution at 200 m/z (Lumos) or 400 m/z (Elite) [FWHM (full width at half maximum)]; **ScR**= scan rate for measurements in the IT; **SR**= scan range [m/z]; **AGC**= automatic gain control, max number of acquired ions per measurement; **AcT**= max. Ion acquisition time [ms]; **CS**= charge states used for fragmentation; **IsM**= Isolation mode (Q or IT), MS2 isolation and further is only done in IT; **IsW**= Isolation window [m/z], value followed by scan mode the isolation is based on (MS1, MS2 …) **Frag.**= Fragmentation method; **HCD**= Higher-energy collisional dissociation; **CID**= Collision-induced dissociation; **ETD**= Electron-transfer dissociation; **EThcD=** Electron-Transfer/Higher-Energy Collision Dissociation; **sHCD**= stepped HCD**; NCE**= normalized collision energy; **cycles**: number of MSn recorded or max cycle time; RF= RF Lens [%]; **SF**= Source Fragmentation [V]; **DDM**: Data dependent Mode (cycle time in seconds, CT/[s] or number of scans, NS); **NS**= Number of data dependent scans

Search Settings

Program & version	MaxQuant v2.0.2.0
Search engine	Andromeda
settings	Basically default; LFQ and MBR were turned on
Static modification	none
Digestion mode	unspecific
	Min. peptide length for unspecific search 6<br>Max. peptide length for unspecific search 36
Dynamic modification	Acetyl (N-term); Oxidation (M)
Modification included in quantification	Oxidation (M)
Databases	1. Contaminants<br>2. ACE_0699_UP000000625_83333.fasta
Annotation	

ACE_0711-04

File legend

ACE ID	Alternate ID	Organ/ cell line	Treatment/ experimental setup
ACE_0711-02_BH01	MS5_H_0	Human (overexpressed in *E. coli*)	2 µM HTRA1 Control, 0 sec, supernatant after acetone precipitation
ACE_0711-02_BH02	MS5_H_0	dito	2 µM HTRA1 Control, 0 sec, supernatant after acetone precipitation
ACE_0711-02_BH03	MS5_H_0	dito	2 µM HTRA1 Control, 0 sec, supernatant after acetone precipitation
ACE_0711-02_BH04	MS5_H_0	dito	2 µM HTRA1 Control, 0 sec, supernatant after acetone precipitation
ACE_0711-02_BH05	MS5_H_120	dito	2 µM HTRA1 Control, 120 min, supernatant after acetone precipitation
ACE_0711-02_BH06	MS5_H_120	dito	2 µM HTRA1 Control, 120 min, supernatant after acetone precipitation
ACE_0711-02_BH07	MS5_H_120	dito	2 µM HTRA1 Control, 120 min, supernatant after acetone precipitation
ACE_0711-02_BH08	MS5_H_120	dito	2 µM HTRA1 Control, 120 min, supernatant after acetone precipitation
ACE_0711-02_BH09	MS5_T_0	dito	10 µM Tau Control, 0 sec, supernatant after acetone precipitation
ACE_0711-02_BH10	MS5_T_0	dito	10 µM Tau Control, 0 sec, supernatant after acetone precipitation
ACE_0711-02_BH11	MS5_T_0	dito	10 µM Tau Control, 0 sec, supernatant after acetone precipitation
ACE_0711-02_BH12	MS5_T_0	dito	10 µM Tau Control, 0 sec, supernatant after acetone precipitation
ACE_0711-02_BH13	MS5_T_120	dito	10 µM Tau Control, 120 min, supernatant after acetone precipitation
ACE_0711-02_BH14	MS5_T_120	dito	10 µM Tau Control, 120 min, supernatant after acetone precipitation
ACE_0711-02_BH15	MS5_T_120	dito	10 µM Tau Control, 120 min, supernatant after acetone precipitation
ACE_0711-02_BH16	MS5_T_120	dito	10 µM Tau Control, 120 min, supernatant after acetone precipitation
ACE_0711-02_BH17	MS5_TH_0	dito	10 µM Tau + 2 µM HTRA1, 0 sec, supernatant after acetone precipitation
ACE_0711-02_BH18	MS5_TH_0	dito	10 µM Tau + 2 µM HTRA1, 0 sec, supernatant after acetone precipitation
ACE_0711-02_BH19	MS5_TH_0	dito	10 µM Tau + 2 µM HTRA1, 0 sec, supernatant after acetone precipitation
ACE_0711-02_BH20	MS5_TH_0	dito	10 µM Tau + 2 µM HTRA1, 0 sec, supernatant after acetone precipitation
ACE_0711-02_BH21	MS5_TH_15	dito	10 µM Tau + 2 µM HTRA1, 15 sec, supernatant after acetone precipitation
ACE_0711-02_BH22	MS5_TH_15	dito	10 µM Tau + 2 µM HTRA1, 15 sec, supernatant after acetone precipitation
ACE_0711-02_BH23	MS5_TH_15	dito	10 µM Tau + 2 µM HTRA1, 15 sec, supernatant after acetone precipitation
ACE_0711-02_BH24	MS5_TH_15	dito	10 µM Tau + 2 µM HTRA1, 15 sec, supernatant after acetone precipitation
ACE_0711-02_BH25	MS5_TH_1	dito	10 µM Tau + 2 µM HTRA1, 1 min, supernatant after acetone precipitation
ACE_0711-02_BH26	MS5_TH_1	dito	10 µM Tau + 2 µM HTRA1, 1 min, supernatant after acetone precipitation
ACE_0711-02_BH27	MS5_TH_1	dito	10 µM Tau + 2 µM HTRA1, 1 min, supernatant after acetone precipitation
ACE_0711-02_BH28	MS5_TH_1	dito	10 µM Tau + 2 µM HTRA1, 1 min, supernatant after acetone precipitation
ACE_0711-02_BH29	MS5_TH_2	dito	10 µM Tau + 2 µM HTRA1, 2 min, supernatant after acetone precipitation
ACE_0711-02_BH30	MS5_TH_2	dito	10 µM Tau + 2 µM HTRA1, 2 min, supernatant after acetone precipitation
ACE_0711-02_BH31	MS5_TH_2	dito	10 µM Tau + 2 µM HTRA1, 2 min, supernatant after acetone precipitation
ACE_0711-02_BH32	MS5_TH_2	dito	10 µM Tau + 2 µM HTRA1, 2 min, supernatant after acetone precipitation
ACE_0711-02_BH33	MS5_TH_5	dito	10 µM Tau + 2 µM HTRA1, 5 min, supernatant after acetone precipitation
ACE_0711-02_BH34	MS5_TH_5	dito	10 µM Tau + 2 µM HTRA1, 5 min, supernatant after acetone precipitation
ACE_0711-02_BH35	MS5_TH_5	dito	10 µM Tau + 2 µM HTRA1, 5 min, supernatant after acetone precipitation
ACE_0711-02_BH36	MS5_TH_5	dito	10 µM Tau + 2 µM HTRA1, 5 min, supernatant after acetone precipitation
ACE_0711-02_BH37	MS5_TH_10	dito	10 µM Tau + 2 µM HTRA1, 10 min, supernatant after acetone precipitation
ACE_0711-02_BH38	MS5_TH_10	dito	10 µM Tau + 2 µM HTRA1, 10 min, supernatant after acetone precipitation

ACE_0711-02_BH39	MS5_TH_10	dito	10 µM Tau + 2 µM HTRA1, 10 min, supernatant after acetone precipitation
ACE_0711-02_BH40	MS5_TH_10	dito	10 µM Tau + 2 µM HTRA1, 10 min, supernatant after acetone precipitation
ACE_0711-02_BH41	MS5_TH_120	dito	10 µM Tau + 2 µM HTRA1, 120 min, supernatant after acetone precipitation
ACE_0711-02_BH42	MS5_TH_120	dito	10 µM Tau + 2 µM HTRA1, 120 min, supernatant after acetone precipitation
ACE_0711-02_BH43	MS5_TH_120	dito	10 µM Tau + 2 µM HTRA1, 120 min, supernatant after acetone precipitation
ACE_0711-02_BH44	MS5_TH_120	dito	10 µM Tau + 2 µM HTRA1, 120 min, supernatant after acetone precipitation

LC_Settings

MS device	Orbitrap Elite
LC device	Evosep One
ion source	Thermo Nanospray Flex
**Analytical column**	EV1109 Analytical Column – 60 & 100 samples/day
column diameter	Length (L_C) = 8 cm; ID = 150; OD = PEEK; emitter EV-1086 Stainless steel emitter
stationary phase	Dr Maisch C18 AQ, 1.5 µm beads
particle diameter (d_p)	1.5 µm
Pore size	120 Å
Column ID	AC_EVO-006
Column oven	na
Column oven temp.	na
**solvents**	A: 0.1% FA in UPLC water<br>B: 0.1% FA in UPLC ACN
gradient	21 min gradient (60 SPD)

MS_Settings

**Project**	**MS**	**general**	**MS1**	**MS2**	**MS2**	**MS3**	**Comments; special settings**
ACE_0644	Elite	Tune v2.7.0.1112 SP2<br>Gradient: 21 min	Analyzer: FT<br>Res.: 60000<br>SR: 300 - 1500<br>AGC: 3×10^6<br>AcT: 50<br>RF: 30<br>SF: --<br>DDM: NS/15	Analyzer: IT<br>Res./ScR: -/rapid<br>SR: Auto<br>AGC: 1×10^4<br>AcT: 60<br>CS: +2 and higher<br>IsM: IT<br>IsW: 2.0<br>Frag.: CID<br>NCE: 35			classic orbitrap experiment: MS1 in Orbitrap at high resolution and data dependent MS2 in Iontrap at rapid scan rate. Dynamic exclusion enabled (exclude after n times=1; Exclusion duration (s)= 30; mass tolerance= ± 100 ppm), exclusion list size: 500

Note: **FT**= Fourier Transform (Orbitrap); **IT**= Iontrap; **Q**= Quadrupol; **Res.**= max. Resolution at 200 m/z (Lumos) or 400 m/z (Elite) [FWHM (full width at half maximum)]; **ScR**= scan rate for measurements in the IT; **SR**= scan range [m/z]; **AGC**= automatic gain control, max number of acquired ions per measurement; **AcT**= max. Ion acquisition time [ms]; **CS**= charge states used for fragmentation; **IsM**= Isolation mode (Q or IT), MS2 isolation and further is only done in IT; **IsW**= Isolation window [m/z], value followed by scan mode the isolation is based on (MS1, MS2 …) **Frag.**= Fragmentation method; **HCD**= Higher-energy collisional dissociation; **CID**= Collision-induced dissociation; **ETD**= Electron-transfer dissociation; **EThcD=** Electron-Transfer/Higher-Energy Collision Dissociation; **sHCD**= stepped HCD; **NCE**= normalized collision energy; **cycles**: number of MSn recorded or max cycle time; RF= RF Lens [%]; **SF**= Source Fragmentation [V]; **DDM**: Data dependent Mode (cycle time in seconds, CT/[s] or number of scans, NS); **NS**= Number of data dependent scans

Search Settings

Program & version	MaxQuant v2.0.2.0
Search engine	Andromeda
settings	Basically default; LFQ and MBR were turned on
Static modification	none
Digestion mode	unspecific
	Min. peptide length for unspecific search 6<br>Max. peptide length for unspecific search 36
Dynamic modification	Acetyl (N-term); Oxidation (M)
Modification included in quantification	Oxidation (M)
Databases	1. Contaminants<br>2. ACE_0699_UP000000625_83333.fasta
Annotation	

ACE_0809-03

File legend

ACE ID	Alternate ID	Organ/ cell line	Treatment/ experimental setup
ACE_0809-03_BH01	MS15_H_0	Human (overexpressed in *E. coli*)	2 µM HTRA1 Control, 0 sec, supernatant after acetone precipitation
ACE_0809-03_BH02	MS15_H_0	dito	2 µM HTRA1 Control, 0 sec, supernatant after acetone precipitation
ACE_0809-03_BH03	MS15_H_0	dito	2 µM HTRA1 Control, 0 sec, supernatant after acetone precipitation
ACE_0809-03_BH04	MS15_H_0	dito	2 µM HTRA1 Control, 0 sec, supernatant after acetone precipitation
ACE_0809-03_BH05	MS15_H_120	dito	2 µM HTRA1 Control, 120 min, supernatant after acetone precipitation
ACE_0809-03_BH06	MS15_H_120	dito	2 µM HTRA1 Control, 120 min, supernatant after acetone precipitation
ACE_0809-03_BH07	MS15_H_120	dito	2 µM HTRA1 Control, 120 min, supernatant after acetone precipitation
ACE_0809-03_BH08	MS15_H_120	dito	2 µM HTRA1 Control, 120 min, supernatant after acetone precipitation
ACE_0809-03_BH09	MS15_T_0	dito	10 µM Tau Control, 0 sec, supernatant after acetone precipitation
ACE_0809-03_BH10	MS15_T_0	dito	10 µM Tau Control, 0 sec, supernatant after acetone precipitation
ACE_0809-03_BH11	MS15_T_0	dito	10 µM Tau Control, 0 sec, supernatant after acetone precipitation
ACE_0809-03_BH12	MS15_T_0	dito	10 µM Tau Control, 0 sec, supernatant after acetone precipitation
ACE_0809-03_BH13	MS15_T_120	dito	10 µM Tau Control, 120 min, supernatant after acetone precipitation
ACE_0809-03_BH14	MS15_T_120	dito	10 µM Tau Control, 120 min, supernatant after acetone precipitation
ACE_0809-03_BH15	MS15_T_120	dito	10 µM Tau Control, 120 min, supernatant after acetone precipitation
ACE_0809-03_BH16	MS15_T_120	dito	10 µM Tau Control, 120 min, supernatant after acetone precipitation
ACE_0809-03_BH17	MS15_TH_0	dito	10 µM Tau + 2 µM HTRA1, 0 sec, supernatant after acetone precipitation
ACE_0809-03_BH18	MS15_TH_0	dito	10 µM Tau + 2 µM HTRA1, 0 sec, supernatant after acetone precipitation
ACE_0809-03_BH19	MS15_TH_0	dito	10 µM Tau + 2 µM HTRA1, 0 sec, supernatant after acetone precipitation
ACE_0809-03_BH20	MS15_TH_0	dito	10 µM Tau + 2 µM HTRA1, 0 sec, supernatant after acetone precipitation
ACE_0809-03_BH21	MS15_TH_15	dito	10 µM Tau + 2 µM HTRA1, 15 sec, supernatant after acetone precipitation
ACE_0809-03_BH22	MS15_TH_15	dito	10 µM Tau + 2 µM HTRA1, 15 sec, supernatant after acetone precipitation
ACE_0809-03_BH23	MS15_TH_15	dito	10 µM Tau + 2 µM HTRA1, 15 sec, supernatant after acetone precipitation
ACE_0809-03_BH24	MS15_TH_15	dito	10 µM Tau + 2 µM HTRA1, 15 sec, supernatant after acetone precipitation

ACE_0809-03_BH25	MS15_TH_1	dito	10 µM Tau + 2 µM HTRA1, 1 min, supernatant after acetone precipitation
ACE_0809-03_BH26	MS15_TH_1	dito	10 µM Tau + 2 µM HTRA1, 1 min, supernatant after acetone precipitation
ACE_0809-03_BH27	MS15_TH_1	dito	10 µM Tau + 2 µM HTRA1, 1 min, supernatant after acetone precipitation
ACE_0809-03_BH28	MS15_TH_1	dito	10 µM Tau + 2 µM HTRA1, 1 min, supernatant after acetone precipitation
ACE_0809-03_BH29	MS15_TH_5	dito	10 µM Tau + 2 µM HTRA1, 5 min, supernatant after acetone precipitation
ACE_0809-03_BH30	MS15_TH_5	dito	10 µM Tau + 2 µM HTRA1, 5 min, supernatant after acetone precipitation
ACE_0809-03_BH31	MS15_TH_5	dito	10 µM Tau + 2 µM HTRA1, 5 min, supernatant after acetone precipitation
ACE_0809-03_BH32	MS15_TH_5	dito	10 µM Tau + 2 µM HTRA1, 5 min, supernatant after acetone precipitation
ACE_0809-03_BH33	MS15_TH_10	dito	10 µM Tau + 2 µM HTRA1, 10 min, supernatant after acetone precipitation
ACE_0809-03_BH34	MS15_TH_10	dito	10 µM Tau + 2 µM HTRA1, 10 min, supernatant after acetone precipitation
ACE_0809-03_BH35	MS15_TH_10	dito	10 µM Tau + 2 µM HTRA1, 10 min, supernatant after acetone precipitation
ACE_0809-03_BH36	MS15_TH_10	dito	10 µM Tau + 2 µM HTRA1, 10 min, supernatant after acetone precipitation
ACE_0809-03_BH37	MS15_TH_2	dito	10 µM Tau + 2 µM HTRA1, 2 min, supernatant after acetone precipitation
ACE_0809-03_BH38	MS15_TH_2	dito	10 µM Tau + 2 µM HTRA1, 2 min, supernatant after acetone precipitation
ACE_0809-03_BH39	MS15_TH_2	dito	10 µM Tau + 2 µM HTRA1, 2 min, supernatant after acetone precipitation
ACE_0809-03_BH40	MS15_TH_2	dito	10 µM Tau + 2 µM HTRA1, 2 min, supernatant after acetone precipitation
ACE_0809-03_BH41	MS15_TH_120	dito	10 µM Tau + 2 µM HTRA1, 120 min, supernatant after acetone precipitation
ACE_0809-03_BH42	MS15_TH_120	dito	10 µM Tau + 2 µM HTRA1, 120 min, supernatant after acetone precipitation
ACE_0809-03_BH43	MS15_TH_120	dito	10 µM Tau + 2 µM HTRA1, 120 min, supernatant after acetone precipitation
ACE_0809-03_BH44	MS15_TH_120	dito	10 µM Tau + 2 µM HTRA1, 120 min, supernatant after acetone precipitation

LC_Settings

MS device	Orbitrap Fusion Lumos
LC device	Evosep One
ion source	Thermo Nanospray Flex
**Analytical column**	EV1064 Analytical Column – 60 & 100 samples/day
column diameter	Length (L_C) = 8 cm; ID = 100; OD = PEEK; emitter EV-1086 Stainless steel emitter
stationary phase	Dr Maisch C18 AQ, 3 µm beads
particle diameter (d_p)	3 µm
Pore size	120 Å
Column ID	AC_EVO-008
Column oven	na
Column oven temp.	na
**solvents**	A: 0.1% FA in UPLC water<br>B: 0.1% FA in UPLC ACN
gradient	21 min gradien (60 SPD)

MS_Settings

**Project**	**MS**	**general**	**MS1**	**MS2**	**MS2**	**MS3**	**Comments; special settings**
ACE_0809	Lumos	Tune v3.5.3881.18<br>Xcalibur v4.5.445.18<br>Gradient: 21 min	Analyzer: FT<br>Res.: 120000<br>SR: 375 - 1600<br>AGC: Standard<br>AcT: Auto<br>RF: 30<br>SF: --<br>DDM: CT/3sec	Analyzer: IT<br>Res./ScR: - /rapid<br>SR: Auto<br>AGC: Standard<br>AcT: Auto<br>CS: +2 to +6<br>IsM: Q<br>IsW: 1.6<br>Frag.: HCD<br>NCE: 32			classic orbitrap experiment: MS1 in Orbitrap at high resolution and data dependent MS2 in Iontrap at turbo scan rate. Dynamic exclusion enabled (exclude after n times=1; Exclusion duration (s)= 20; mass tolerance= ± 10ppm)<br>intensity threshold: 5 × 10³

Note: **FT**= Fourier Transform (Orbitrap); **IT**= Iontrap; **Q**= Quadrupol; **Res.**= max. Resolution at 200 m/z (Lumos) or 400 m/z (Elite) [FWHM (full width at half maximum)]; **ScR**= scan rate for measurements in the IT; **SR**= scan range [m/z]; **AGC**= automatic gain control, max number of acquired ions per measurement; **AcT**= max. Ion acquisition time [ms]; **CS**= charge states used for fragmentation; **IsM**= Isolation mode (Q or IT), MS2 isolation and further is only done in IT; **IsW**= Isolation window [m/z], value followed by scan mode the isolation is based on (MS1, MS2 …) **Frag.**= Fragmentation method; **HCD**= Higher-energy collisional dissociation; **CID**= Collision-induced dissociation; **ETD**= Electron-transfer dissociation**; EThcD=** Electron-Transfer/Higher-Energy Collision Dissociation; **sHCD**= stepped HCD**; NCE**= normalized collision energy; **cycles**: number of MSn recorded or max cycle time; RF= RF Lens [%]; **SF**= Source Fragmentation [V]; **DDM**: Data dependent Mode (cycle time in seconds, CT/[s] or number of scans, NS); **NS**= Number of data dependent scans

Search Settings

Program & version	MaxQuant v2.0.3.0
Search engine	Andromeda
settings	Basically default; LFQ and MBR were turned on
Static modification	none
Digestion mode	unspecific
	Min. peptide length for unspecific search 6<br>Max. peptide length for unspecific search 36
Dynamic modification	Acetyl (N-term); Oxidation (M); Phospho (STY)
Modification included in quantification	Oxidation (M);Acetyl (Protein N-term)
Databases	3. Contaminants<br>1. ACE_0809_SOI_v01.fasta
Annotation	

Supporting data 7. Example for how UMSAP calculates the relative frequency of cuts

In a MS experiment the following peptides were identified. They all share the same P1 site at V231

```
A -  RGHYV
B -   GHYV
C -       DFRTGH
D -       DFRTGHKL
E -       DFRTGHKLM
F -       DFRTGHKLMT
```

Average intensities. 0 values mean that the peptide was not detected in the given time point or the intensity values are not significantly different to the control experiments.

Time Point	A	B	C	D	E	F
5 min	2	0	0	3	3	10
15 min	4	1	0	0	6	5
30 min	8	3	5	0	0	1

The first non-zero average intensity along the time points is taken as reference for the calculation of the relative intensity.

	Relative Intensities						Relative frequency of cuts
Time-point	A	B	C	D	E	F	P1 (V231)
5 min	2/2 = 1	0/1 = 0	0/5 = 0	3/3 = 1	3/3 = 1	10/10 = 1	1+0+0+1+1+1 = 4
15 min	4/2 = 2	1/1 = 1	0/5 = 0	0/3 = 0	6/3 = 2	5/10 = 0.5	2+1+0+0+2+0.5 = 5.5
30 min	8/2 = 4	3/1 = 1	5/5 = 1	0/3 = 0	0/3 = 0	1/10 = 0.1	4+1+1+0+0+0.1 = 6.1

Table S1. P1 residues in tau with the highest relative number of cuts

Relative number of cuts at 120 min

Residue	Sol tau	Fibrillar tau	Fibrillar tau CN
V256	22	40	33
C291 (β)	24		
I308 (β)	42		
C322	32		
L376	27	59	20
I392	51	49	
L408	29	40	25
V411		25	

Class I P1 residues (> 20 cuts) detected in proteolysis experiments with various tau constructs and conformations at the 120 min timepoint. (β) indicates residues of the β-sheets of the fibrillar core.

Table S2. P1 residues in tau with the second highest relative number of cuts

Relative number of cuts at 120 min

Residue	Sol tau	Fibrillar tau	Fibrillar tau CN
V226		15	13
L266	11	15	12
I277(β)	13		
I278(β)	12	11	
V287(β)	13		10
S289(β)	10		
I308(β)	>20	17	15
I328	13		
L344	15	18	14
I360		17	17
I392	>20	>20	15
V398		12	
V411	16	>20	14
L428	13	15	14
V432	12	14	13

Class II P1 residues (10 - 19 cuts) detected in proteolysis experiments with various tau constructs and conformations at the 120 min timepoint. (β) indicates residues of the β-sheets of the fibrillar core.

Table S3. P1 residues that are differentially cleaved in soluble and fibrillar tau at the 120 min time point

Relative number of cuts at 120 min

Residue	Sol tau	Fibrillar tau	Fibrillar tau CN
V275	6	2	1
Q276	-	2	3
I277	13	7	6
I278(β)	12	11	2
N279(β)	-	-	1
K280(β)	7	-	3
L282(β)	6	-	-
L284(β)	2	-	1
S285(β)	2	2	-
V287(β)	13	9	10
S289(β)	10	4	2
C291(β)	24	2	1
V306(β)	2	-	-
Q307(β)	1	-	2
I308(β)	42	17	15
V309(β)	9	2	3
Y310(β)	2	-	1
V313(β)	8	-	-
K317(β)	1	2	-
V318(β)	9	4	7
N327(β)	1	-	-
I328(β)	13	1	-

Relative number of cuts at P1 residues indicated. No number corresponds to no cleavages detected. (β) indicates residues of the β-sheets of the fibrillar core.

Supporting Videos

Movie S1. Cyto tau fibrils (green), extracell HTRA1S328A (red)

Live-cell timelapse confocal spinning disk microscopy of cells containing cytosolic tau seeds (green), which were treated with extracellular proteolytically inactive HTRA1S328A (red). Uptake and colocalization of HTRA1 with tau seeds was followed over 30 min. Colocalized particles appear white. See text for details.

Movie S2. Cyto HTRA1S328A (red), extracell tau fibrils (green)

Live-cell timelapse confocal spinning disk microscopy of cells containing cytosolic HTRA1S328A (red), which were treated with extracellular tau seeds (green). Uptake and colocalization of tau seeds with proteolytically inactive HTRA1S328A was followed over 30 min. Colocalized particles appear white. See text for details.

Movie S3. Tau + HTRA1

Live-cell timelapse confocal spinning disk microscopy of cells containing cytosolic tau seeds (green), which were treated with extracellular proteolytically active HTRA1 (red). The level of tau seeds was followed over 30 min. See text for details.

Movie S4. PBS-Control only Tau

Control experiment for Movie S3. Live-cell timelapse confocal spinning disk microscopy of cells containing cytosolic tau seeds (green), which were treated with PBS. The level of tau seeds was followed over 30 min. See text for details.
